# Magnetism, Mössbauer Spectroscopy, and Proton Conductivity of Coordination Polymers Based on Phosphonate and Phosphinate Linkers

**DOI:** 10.1021/acs.jpcc.5c04260

**Published:** 2025-08-20

**Authors:** Soňa Ondrušová, Daniel Bůžek, Jan Hynek, Tomáš Plecháček, Lenka Kubíčková, Miroslav Veverka, Tomáš Kmječ, Matyáš Rozprým, Jaroslav Kohout, Jan Demel, Matouš Kloda

**Affiliations:** † 112895 Institute of Inorganic Chemistry of the Czech Academy of Sciences, Husinec-Řež 1001, 250 68 Řež, Czech Republic; ‡ Department of Inorganic Chemistry, Faculty of Science, 37740 Charles University, Hlavova 2030, Prague 12840, Czech Republic; § Center of Materials and Nanotechnologies, Faculty of Chemical Technology, 48252 University of Pardubice, Cs. Legii 565, 530 02 Pardubice, Czech Republic; ∥ Faculty of Mathematics and Physics, 138735 Charles University, V Holešovičkách 2, 180 00 Prague, Czech Republic; ⊥ Institute of Physics of the Czech Academy of Sciences, Cukrovarnická 10/112, 162 00 Prague, Czech Republic

## Abstract

Coordination polymers (CPs) are versatile materials formed by metal ions and organic ligands, offering a broad range of structural and functional possibilities. Phosphonates and phosphinates are particularly attractive ligands for CPs due to their multiple binding sites, varied coordination geometries, and ability to form robust network structures. Phosphonates, considered harder ligands, form strong bonds with hard metals such as Fe^3+^, while phosphinates offer additional versatility due to the varied pendant groups on phosphorus. This study presents a series of six new coordination polymers, ICR-20 and ICR-21, incorporating Fe^2+^, Co^2+^, and Ni^2+^ metal centers, using phosphinate (H_2_PBP­(Me)) or phosphinate–phosphonate (H_3_PPP­(Me)) ligands in combination with 4,4′-bipyridine. The materials are isoreticular despite the incorporation of different functional groups, demonstrating the interchangeability of the phosphinate and phosphonate groups in their design. These polymers were characterized structurally and investigated for their magnetic properties. The combination of local insights from Mössbauer spectroscopy and bulk magnetic data provides complex information on crystal field parameters and magnetic interactions in Fe-based polymers. Additionally, their proton conductivity was evaluated, showing promising results.

## Graphical abstract

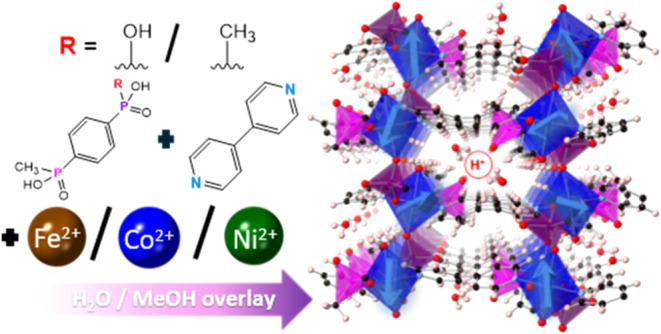

## Introduction

Coordination polymers (CPs) are a class of materials formed by the assembly of metal ions and organic ligands into extended networks. The vast diversity of available ligands, metal centers, and their coordination modes results in a great variability in the properties and architectures of these materials. This inherent versatility allows CPs to be tailored for a wide range of applications, including catalysis,^1,2^ drug delivery,^3,4^ sensing,^5,6^ and magnetic applications.^7–9^

Phosphonates with the general formula RPO_3_H_2_ offer multiple binding sites and varied coordination geometries,^10^ enabling the formation of complex and robust network structures. According to Pearson’s theory of hard and soft acids and bases,^11^ phosphonates are considered harder ligands than carboxylates, which results in the formation of stronger bonds with hard metals such as Fe^3+^.^12^ Phosphinates with the general formula R^1^R^2^PO_2_H bring additional versatility to the pendant group on phosphorus, formally replacing one of the phosphonate −OH groups. It has been demonstrated that the isoreticular design of coordination polymers is possible not only with phosphinates containing different pendant groups,^13,14^ but can be extended by a partial or full replacement of phosphinates by phosphonates as well.^15^ Despite these benefits, relatively little focus is given to phosphinate coordination polymers compared to phosphonates or carboxylates.^16^

Magnetic properties of coordination polymers have attracted attention for their potential applications in diverse fields such as data storage^17,18^ and spintronics.^19–22^ It has been demonstrated that M–O–P–O–M bridges, a common structural motif in phosphinate coordination polymers, can mediate magnetic exchange.^23,24^ Furthermore, the strength of the magnetic coupling depends on the bonding parameters and can be influenced by ancillary ligands.^25^

Proton conductivity of coordination polymers is attracting attention due to possible use in proton-exchange membrane fuel cells (PEMFCs).^26,27^ Proton conduction is often facilitated by channels in a porous structure; however, extended hydrogen-bonded networks can provide conduction pathways even in structures with no detectable porosity.^28–30^ Phosphonates in particular attract attention as building blocks for proton conductive coordination polymers due to the robust nature of the coordination polymers and presence of excessive −OH groups, part of which can remain uncoordinated and participate in hydrogen bonding.^31–33^

In the present study, we developed a series of coordination polymers, ICR-20 and ICR-21, incorporating Fe^2+^, Co^2+^, and Ni^2+^ metal centers with phosphinate (H_2_PBP­(Me)) – phenylene-1,4-bis­(methylphosphinic acid) or mixed phosphinate–phosphonate (H_3_PPP­(Me)) – (4-[hydroxy­(methyl)­phosphoryl]­phenylphosphonic acid) ligands in combination with 4,4′-bipyridine as a coligand. These CPs were fully characterized, and their magnetic properties and proton conductivity were evaluated through extensive measurements.

## Experimental Section

The phosphinate ligands were synthesized according to the procedures described in earlier articles.^15,34^ The following chemicals used for the preparation of the coordination polymers are commercially available and were used as purchased: 4,4′-bipyridine, Ni­(NO_3_)_2_·6H_2_O, FeSO_4_·7H_2_O (both Merck), Co­(NO_3_)_2_·6H_2_O (Lachema, Czech Republic), acetone, EtOH (99%), and MeOH (all Lach:ner, Czech Republic).

Single-crystal X-ray diffraction analyses for all crystalline products were performed using a Rigaku XtaLAB Synergy S diffractometer equipped with a Cu (Cu/Kα radiation; λ = 1.54184 Å) microfocus X-ray source and a Hybrid Pixel Array Detector (HyPix-6000HE). The Ni-ICR-20 sample was kept at 95 K, and all other samples were kept at 100 K during the data collection using an Oxford Cryosystems (Cryostream 800) cooling device. CrysAlis Pro software was used for the data collection, cell refinement, data reduction, and absorption correction.^35^ Data were corrected for absorption effects using an empirical absorption correction (spherical harmonics), implemented in the SCALE3 ABSPACK scaling algorithm, and a numerical absorption correction based on Gaussian or analytical integration over a multifaceted crystal model. The structures were solved with the ShelXT^36^ structure solution program using Intrinsic Phasing and refined with the ShelXL refinement package^37^ using least-squares minimization as implemented in Olex2.^38^ Anisotropic displacement parameters were refined for all non-H atoms. The hydrogen atoms were localized on a difference Fourier map or calculated in idealized positions. The structures of Fe-ICR-20 and Co-ICR-20 were refined as 2-fold and 3-fold nonmerohedral twins, respectively.

Powder X-ray diffraction data (PXRD) were recorded using a PANalytical X’Pert PRO diffractometer in the Bragg–Brentano reflection geometry, equipped with a Cu anode (40 kV, 30 mA) (in the case of Ni samples) or Co anode (in the case of Fe and Co samples) and a linear PIXcel detector. FTIR spectra were collected with a Nicolet NEXUS 670-FT spectrometer in the ATR configuration. Thermal analyses (TG/DTA/MS) were carried out on a Setaram SETSYS Evolution-16-MS instrument coupled to a mass spectrometer. The measurements were performed in synthetic air (flow rate 30 mL min^–1^) from 30 to 750 °C with a heating rate of 5 °C min^–1^. CHN elemental analysis was performed by a standard combustion technique with the Thermo Scientific FlashSmartTM 2000Elemental analyzer. The adsorption of water vapor was measured by using a Belsorp MAX II instrument. The measurement was carried out at 298 K. Before the measurement, the sample was degassed at 100 °C for 16 h under a dynamic vacuum.

### Preparation of Fe-ICR-20

27.8 mg of FeSO_4_·7H_2_O (0.1 mmol) was dissolved in 1 mL of H_2_O and overlayered by a solution of 23.4 mg H_2_PBP­(Me) (0.1 mmol) and 15.6 mg of 4,4′-bipyridine (0.1 mmol) in 1 mL of MeOH, 0.1 mL of 1 M solution of NaOH, and 0.4 mL of H_2_O. The mixture was then left undisturbed for 1 week. Afterward, red crystals were collected, manually separated from white powder (ICR-2), washed with EtOH, and air-dried.

### Preparation of Co-ICR-20

29.1 mg of Co­(NO_3_)_2_·6H_2_O (0.1 mmol) were dissolved in 1 mL of H_2_O and overlayered by a solution of 23.4 mg H_2_PBP­(Me) (0.1 mmol) and 15.6 mg of 4,4′-bipyridine (0.1 mmol) in 1 mL of MeOH, 0.1 mL of 1 M solution of NaOH, and 0.4 mL of H_2_O. The mixture was then left undisturbed for 1 week. The resulting pink crystals were collected, washed with EtOH, and air-dried.

### Preparation of Ni-ICR-20

29.1 mg of Ni­(NO_3_)_2_·6H_2_O (0.1 mmol), 15.6 mg of 4,4′-bipyridine (0.1 mmol), and 23.4 mg H_2_PBP­(Me) were dissolved in 2 mL of H_2_O and overlaid by 4 mL of acetone. The mixture was then left undisturbed for 3 days. The resulting light green powder was washed with acetone and air-dried.

### Preparation of Fe-ICR-21

27.8 mg of FeSO_4_·7H_2_O (0.1 mmol) was dissolved in 1 mL of H_2_O and overlayered by a solution of 23.6 mg H_2_PPP­(Me) (0.1 mmol) and 15.6 mg of 4,4′-bipyridine (0.1 mmol) in 1 mL of MeOH, 0.1 mL of 1 M solution of NaOH, and 0.4 mL of H_2_O. The mixture was then left undisturbed for 1 week. The resulting red crystals were collected, manually separated from white powder (ICR-12 ^15^), washed with EtOH, and air-dried.

### Preparation of Co-ICR-21

29.1 mg of Co­(NO_3_)_2_·6H_2_O (0.1 mmol) was dissolved in 1 mL of H_2_O and overlayered by solution of 23.6 mg H_2_PPP­(Me) (0.1 mmol) and 15.6 mg of 4,4′-bipyridine (0.1 mmol) in 1 mL of MeOH, 0.1 mL of 1 M solution of NaOH and 0.4 mL of H_2_O. The mixture was then left undisturbed for 1 week. The resulting pink crystals were collected, washed with EtOH, and air-dried.

### Preparation of Ni-ICR-21

29.1 mg of Ni­(NO_3_)_2_·6H_2_O (0.1 mmol) was dissolved in 1 mL of H_2_O and overlayered by solution of 23.6 mg H_2_PPP­(Me) (0.1 mmol) and 15.6 mg of 4,4′-bipyridine (0.1 mmol) in 1 mL of MeOH, 0.1 mL of 1 M solution of NaOH, and 0.4 mL of H_2_O. The mixture was then left undisturbed for 1 week. The resulting green crystals were collected, washed with EtOH, and air-dried.

### Magnetic Properties

Magnetic measurements were performed by using a Quantum Design (QD) Physical Property Measurement System (PPMS 9) with a vibrating sample magnetometer. Powder samples were mounted in the polypropylene VSM powder sample holders by QD. Magnetization isotherms, i.e., *M*(*H*) loops, were measured at the range of intensity of applied magnetic field ± 7 T for selected temperatures ranging from 2 to 300 K. The DC magnetic susceptibility measurements were carried out in various applied fields for the temperature range of 2–300 K (temperature sweep 1–2 K/min) at zero-field-cooled (ZFC), field-cooled cooling (FCC) and field-cooled warming (FCW) regimes. The intensity of the applied magnetic field varied from 0.1 to 7 T, and the susceptibility was approximated by χ = *M*/*H*. The data were corrected for the diamagnetic contribution of the sample holder, while the approximate diamagnetic susceptibility resulting from the remaining diamagnetism of the samples was included in the fit as a part of the temperature-independent χ_TI_ (eq 6 in the Supporting Information; the approximate correction by using Pascal constants would reach −(2.5−2.6)·10^–4^ cm^3^ mol^–1^, though it was not included due to the complexity of ligands). A small thermal hysteresis was observed in the susceptibility data measured during warming and cooling, accentuated in the χ*T* representation. This was identified as a measurement artifact, so the susceptibility data presented were obtained by averaging the ZFC and FCC curves. These average curves were also used in the Curie–Weiss fits. As the data were measured with a high density of points, roughly 10–20 points were skipped in visualization (empty points in Figures 5 and 6). The corrected magnetization and average susceptibility curves were fitted simultaneously in PHI v3.1.6,^40^ using a single-ion approximation. To obtain an estimate of the exchange interaction between the metal ions in the chain, we also considered two interacting magnetic centers (fits with three centers provided similar results). The spin-only approximation was employed, where higher states admixing to the ground state result in a small orbital contribution manifested by an anisotropic g-factor deviating from 2. Only the second-order crystal field parameters were considered in the fit.

### Mössbauer Spectroscopy

The transmission Mössbauer spectra of the Fe-ICR-20 and Fe-ICR-21 samples were collected with a conventional constant-acceleration spectrometer (WissEL, Ortenberg, Germany) equipped with a ^57^Co/Rh source at room temperature. The velocity calibration of the spectrometer and determination of isomer shifts are given with respect to the room-temperature ^57^Fe Mössbauer spectrum of an α-Fe foil. The spectra were recorded at a 12 mm s^–1^ velocity sweep. The Mössbauer spectroscopy experiments at low temperatures in an external magnetic field were performed in an SVT-400 bath cryostat (Janis Research, Woburn, MA). The direction of the external magnetic field is perpendicular to the γ-ray direction. For low-temperature measurements, the sample was wrapped in an aluminum foil, which ensures good thermal contact between the sample and the thermometer. As the foil contains a small amount of Fe, it contributes to the spectra with a small doublet with *IS* ∼ 0.3 mm s^–1^ and *QS ∼* 0.4 mm s^–1^ (D_3_ in Fe-ICR-20 with integral intensity *I* ∼ 2%). All of the recorded ^57^Fe Mössbauer spectra were evaluated using the current version of Confit^41^ and MossWinn^42^ fitting software. The in-field spectra were fitted by using the slow-relaxation Paramagnetic Hyperfine Structure model (4–20 K), implemented in MossWinn. The spectra at 4.2 K at various applied fields were fitted simultaneously, sharing field-independent parameters. Similarly, the simultaneous fit, sharing temperature-independent parameters, was applied to spectra at 6 T and different temperatures. At higher temperatures (30–100 K), the Mixed Q + M Static Hamiltonian model for powder samples was employed, where the reference coordinate system corresponds to the eigensystem of the EFG tensor.

### Proton Conductivity

Samples for the conductivity measurements were prepared by pressing powdered materials into round pellets with a thickness *L* of approximately 1 mm using a pressure of 92 MPa, to which 0.2 cm^2^ Au-coated stainless steel electrodes were mechanically pressed. The conductivity of the samples was measured with a Metrohm Autolab PGStat12 instrument in a frequency range from 0.1 Hz to 1 MHz with a signal amplitude of 200 mV. The impedance data in a complex impedance plot were analyzed by an equivalent circuit approach using ZSimpWin software.^43^ The chosen equivalent electrical circuit used for fitting consisted of a parallel arrangement of the resistance *R*_1_, *R*_2_, and a constant phase element (CPE), as defined by Barsoukov and Macdonald.^44^ The fit provides the value of the resistance *R*. For the calculation of the proton conductivity σ of the samples, we used the relationship σ = *L*/*RA*, where *A* is the area of the electrodes and *L* stands for the distance between them.

## Results and Discussion

The coordination polymers were prepared at room temperature by using a layering process. The selected metal salt was dissolved in water, while H_2_PBP­(Me) or H_3_PPP­(Me) with 4,4′-bipyridine was dissolved in a mixture of water, MeOH, and NaOH. The solution containing the ligands was carefully overlaid on top of the metal salt solution. In all cases, equimolar amounts of NaOH and the ligand were used. Notably, when the NaOH to ligand ratio was increased to 3:1, i.e., equimolar amount of NaOH and acidic hydrogens, a different phase was obtained with H_3_PPP­(Me) and Fe^2+^ and Co^2+^ as the metal centers. This phase, denoted ICR-22, was not further studied; details are provided in the SI Section 6. For Ni-ICR-20, all components were mixed in water and overlaid with acetone, as detailed in the Experimental Section. In the case of Fe-ICR-20 and Fe-ICR-21, partial oxidation of Fe^2+^ to Fe^3+^ occurred, resulting in the formation of previously reported ICR-2 and ICR-12 phases (Figure S8) in the form of a white powder, which was manually separated.

The presented coordination polymers, ICR-20 and ICR-21, are isoreticular and crystallize in the monoclinic *P2*_1_ */n* space group with empirical formula M­(PBP­(Me))­(bipy)·2H_2_O for ICR-20 and M­(HPPP­(Me))­(bipy)·2H_2_O for ICR-21. The structures are similar to the bisphosphonate coordination polymers reported by Rautenberg et al., which have been studied for their proton conductivity.^31^ The lattice parameters for all measured structures are summed up in Table 1, and full crystallographic information is shown in Table S2.

**Table 1:** Lattice Parameters of ICR-20 and ICR-21

	*a* (Å)	*b* (Å)	*c* (Å)	β (°)
Fe-ICR-20	11.0668(5)	9.6700(5)	19.1301(9)	94.133(4)
Co-ICR-20	11.0771(6)	9.6169(7)	19.008(1)	93.839(4)
Ni-ICR-20	11.0682(2)	9.5592(1)	18.8123(2)	93.502(1)
Fe-ICR-21	10.9579(1)	9.4636(1)	19.0326(2)	95.508(1)
Co-ICR-21	11.0000(3)	9.3583(2)	18.8935(4)	95.061(2)

Metal cations in all structures adopt a slightly distorted octahedral geometry with oxygen atoms in equatorial positions and nitrogen atoms in axial positions (bond lengths and angles for coordinated atoms are shown in Tables S3 and S4). Three coordinated oxygen atoms come from two phosphinate or phosphonate groups, one coordinated in bridging geometry and one coordinated by only one oxygen atom, and the remaining oxygen atom comes from one coordinated water molecule. The octahedra are linked by the phosphinate or phosphonate groups into zigzag chains propagating along the *b* axis with a *M*–*M* distance of 5.402–5.592 Å. Together with the ligand backbones, the chains form sheets in the *ab* plane, which are linked by the bipyridine molecules into a three-dimensional (3D) network. The structure contains voids occupied by water molecules; however, no porosity was detected in the adsorption measurements. The structures of Co-ICR-20 and Co-ICR-21 are shown in Figures 1 and 2, respectively.

**Figure 1:**
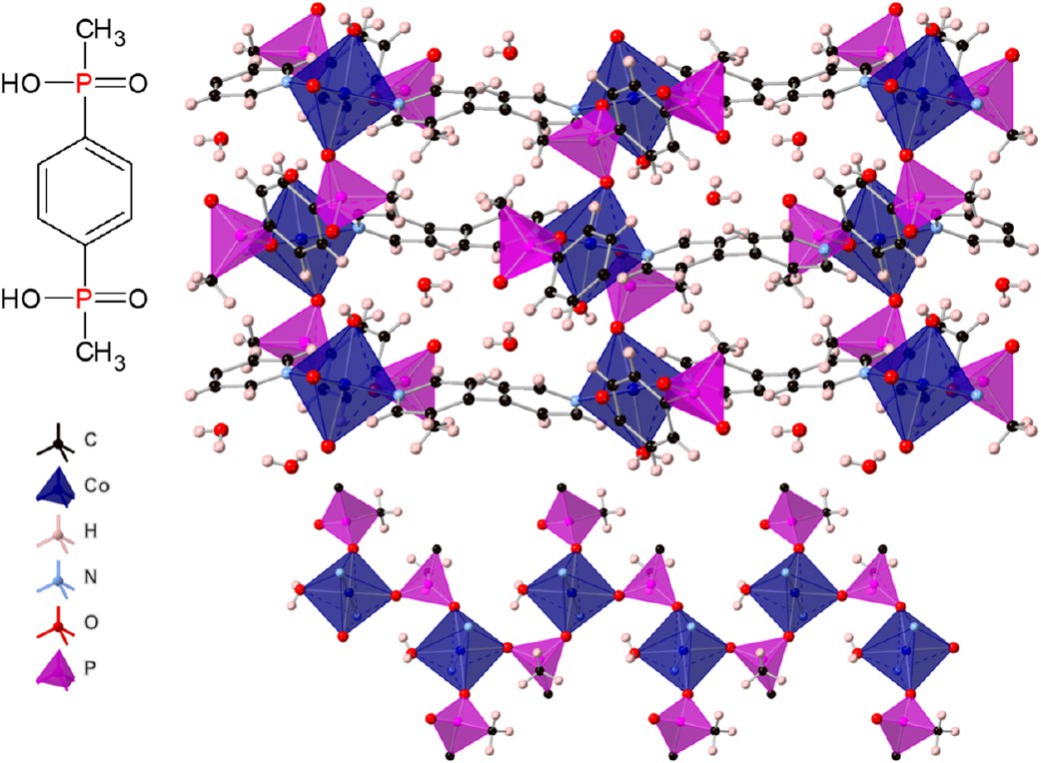
View of the coordination network of Co-ICR-20 (top) and the chain arrangement of phosphinate groups and metal centers (bottom).

**Figure 2:**
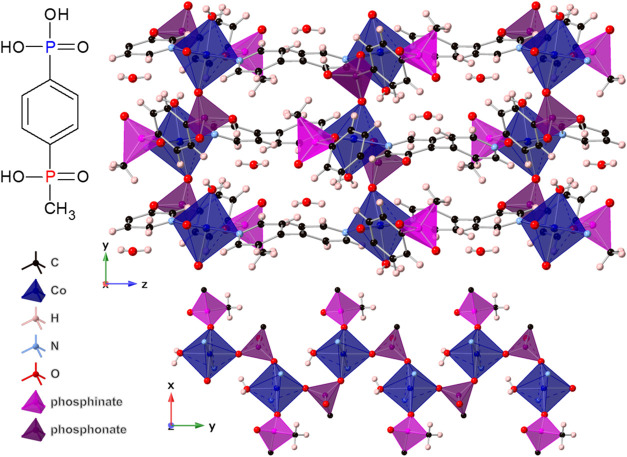
View of the coordination network of Co-ICR-21 (top) and the chain arrangement of phosphonate groups and metal centers (bottom).

Powder X-ray diffraction (PXRD) patterns are in good agreement with theoretical patterns calculated from the crystal structures, which confirms the phase purity of the prepared coordination polymers (Figures S1–S5). The only exception in this regard is Ni-ICR-20, where a small amount of another phase was detected. Very small amounts of impurity were also detected in Fe-ICR-21. The PXRD pattern of Ni-ICR-21, for which single crystals suitable for structure determination could not be obtained, is in good agreement with the patterns simulated for the analogous materials containing Fe or Co as the metal centers (Figure S6). Therefore, it can be concluded that the obtained product is isostructural with the other ICR-21 phases despite the absence of single-crystal data.

FTIR spectra of all ICR-20 and ICR-21 coordination polymers are very similar. All spectra contain multiple strong bands in the P=O stretching region (1000–1200 cm^–1^), which correspond to the presence of two inequivalent phosphinate or phosphonate groups in different coordination modes. The differences between ICR-20 and ICR-21 caused by the presence of −CH_3_ groups instead of −OH are consistent across all metal ions. Notable differences can be seen in the region below 600 cm^–1^, although the interpretation of those features is not straightforward. The peak at 750 cm^–1^ in the spectra of ICR-20 and at 910 cm^–1^ in ICR-21 can be attributed to P–C and P–OH stretching vibration, respectively.^45^ Broad band over 3000 cm^–1^ confirms the presence of hydrogen–bonded water molecules in the structure. Details of the spectra highlighting the differences between ICR-20 and ICR-21 are shown in Figure 3; full FTIR spectra of all products are shown in Figures S10–S15.

**Figure 3:**
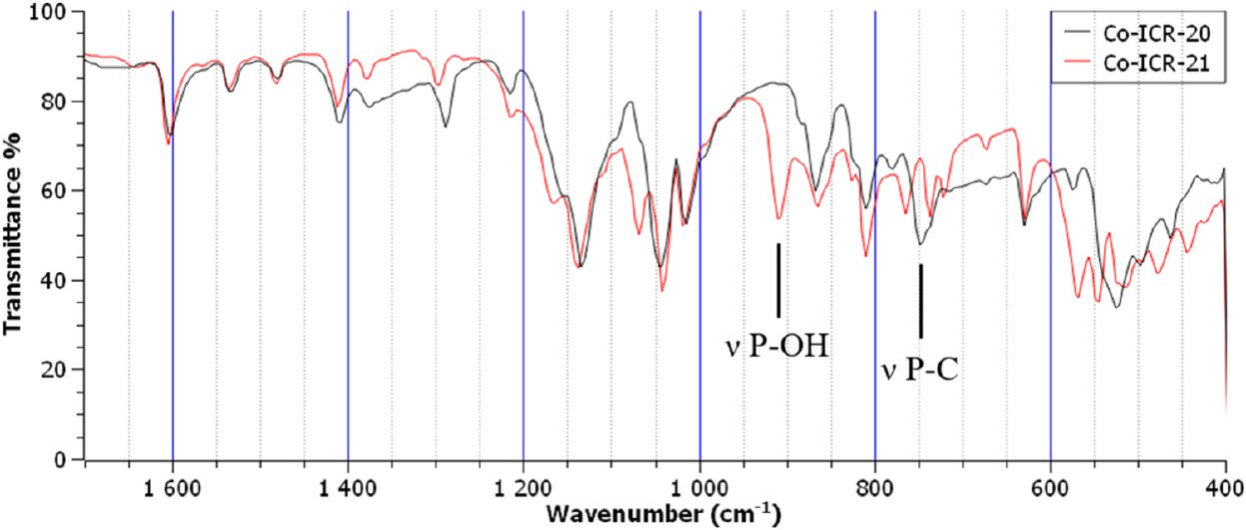
FTIR spectra of Co-ICR-20 and Co-ICR-21.

Thermogravimetric analyses (Figures S16 and S21) show that all coordination polymers release water between 93 and 136 °C for ICR-20 and between 118 and 173 °C for ICR-21. Coordination polymers containing phosphonate groups retain water more strongly, which is consistent with the presence of additional −OH groups in phosphonate ligands that facilitate hydrogen bonding. Further increase of the temperature leads to the thermolysis of the ligands, as indicated by the release of CO_2_. The thermal stability of the investigated coordination polymers increases in the order Fe < Co < Ni in both series, with decomposition temperatures ranging from 242 to 353 °C for ICR-20 and from 207 to 355 °C for ICR-21. The observed increase in thermal stability from Fe to Ni aligns with the trends reported in the literature.^31,46^ These results indicate that in our series, purely phosphinate-based materials exhibit greater thermal stability than those partially containing phosphonate groups. This is in line with the results obtained for similar MOFs composed exclusively of phosphonate ligands. Although the measurement conditions differ (ICR samples were not measured under inert conditions), the trend of increasing thermal stability with higher phosphinate content remains evident.^31^ The same trend is also observed in previously published isoreticular MOFs (ICR-2, ICR-12, and ICR-13), where an increasing phosphonate content results in reduced thermal stability.^15,34^

To better understand the dehydration/rehydration process, we measured the adsorption of water vapor in Fe-ICR-21 (Figure 4) at 298 K. The isotherm shows a steady increase in adsorbed amount reaching up to 4 mmol/g, which corresponds to two water molecules per formula unit and fits well with the 8% mass decrease detected by thermogravimetric analysis. The desorption branch does not follow the adsorption, indicating that only partial dehydration can be achieved without heating. The PXRD pattern measured after water adsorption (Figure S9) confirms the stability of Fe-ICR-21 during the dehydration/rehydration cycle.

**Figure 4:**
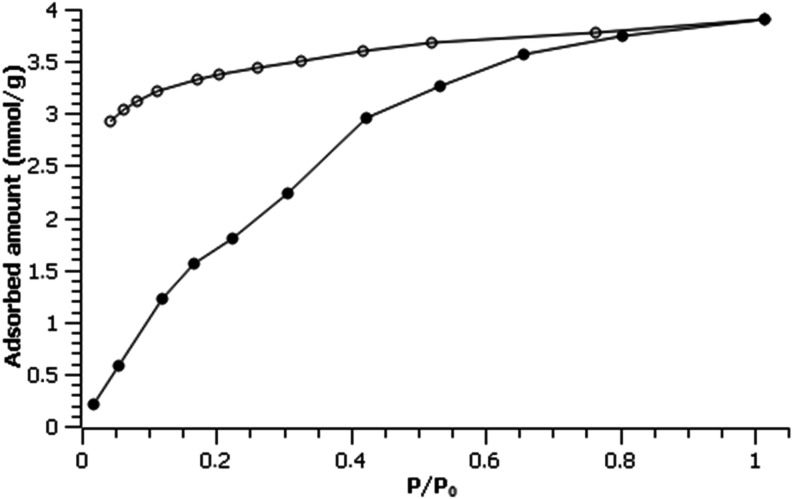
Water adsorption (full points) and desorption (empty points) isotherm for Fe-ICR-21 at 298 K.

To gain better insight into the structure and morphology, SEM images of all presented coordination polymers were taken (Figure 5). The images show the microcrystalline character of the materials with a rather irregular shape of the crystallites. Uniform crystal morphology was observed for the majority of products; however, in the case of Co-ICR-20, the SEM images show two distinct types of crystals, although the respective PXRD pattern (Figure S2) reveals the presence of only a single crystalline phase corresponding to the resolved crystal structure. In the SEM image of Ni-ICR-20, the impurity detected by PXRD can be seen.

**Figure 5:**
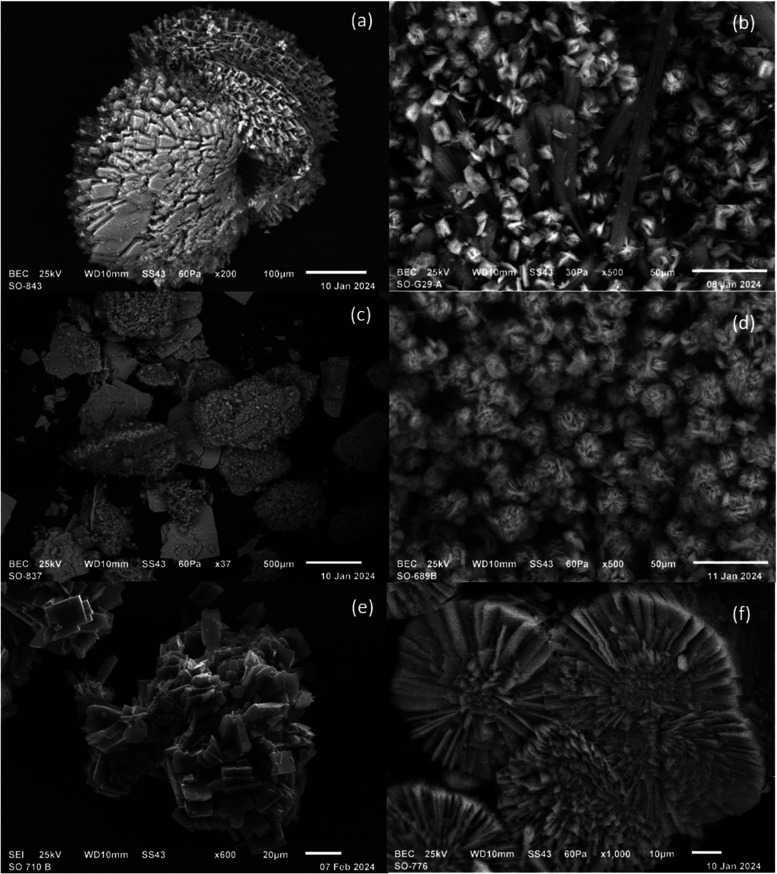
SEM images of Fe-ICR-20 (a), Co-ICR-20 (b), Ni-ICR-20 (c), Fe-ICR-21 (d), Co-ICR-21 (e), and Ni-ICR-21 (f).

### Magnetic Properties

Since it was possible to obtain isostructural coordination polymers containing different metal centers, we could make an interesting study comparing the magnetic properties of Ni^2+^ (3d^8^, *S* = 1), Co^2+^ (3d^7^, *S* = 3/2), and Fe^2+^ (3d^6^, *S* = 2) ions in an analogical coordination environment of uniform metal-phosphonate or phosphinate chains. The χ*T* plots at various applied fields and magnetization curves at selected temperatures are depicted in Figures 6 and 7. No long-range order was found down to 2 K.

**Figure 6:**
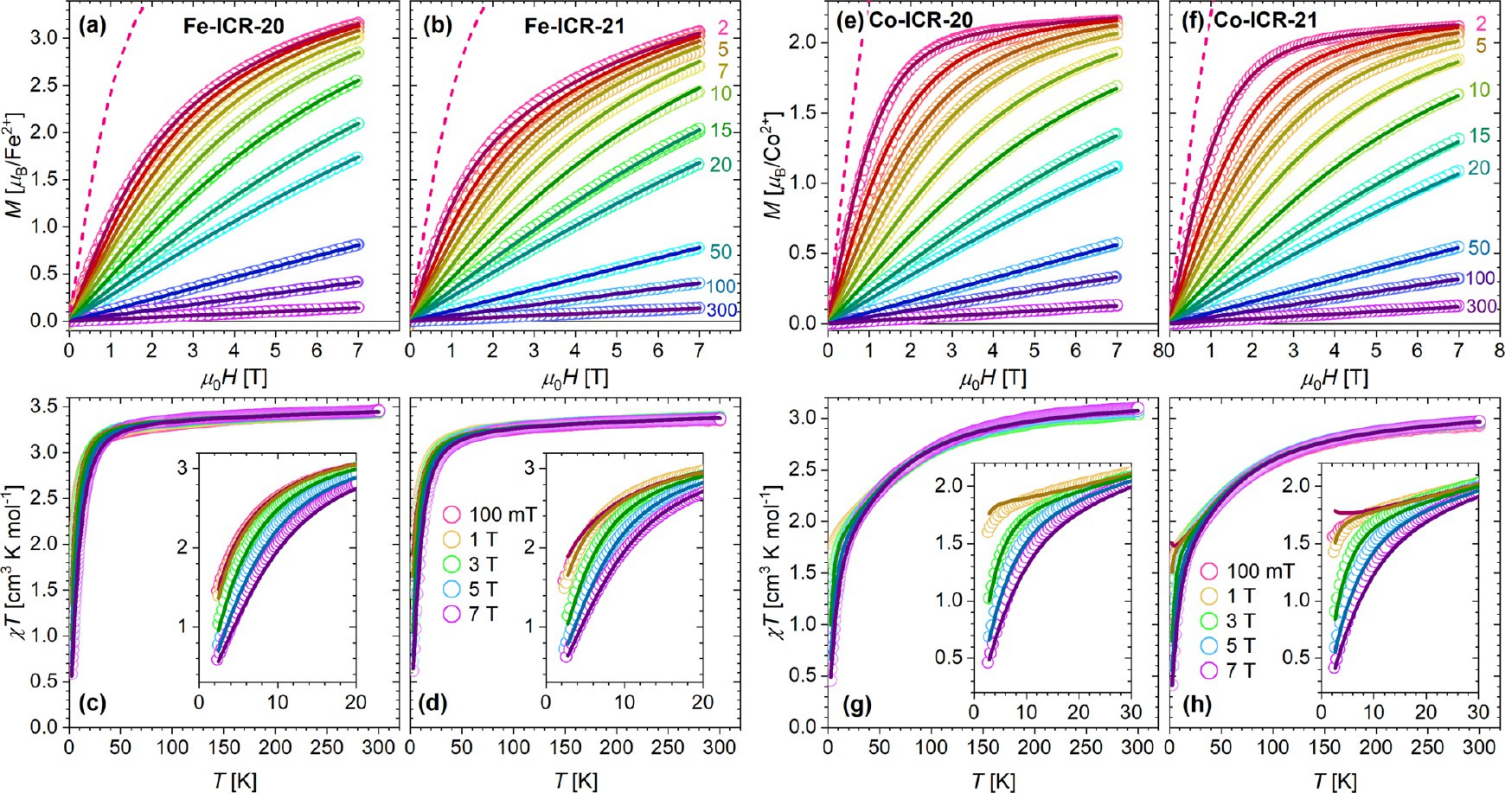
Field-dependent magnetization curves at various temperatures (a, b and e, f for Fe- and Co-based coordination polymers, respectively) and the product of DC susceptibility and temperature, χ*T*, at various fields (c, d and g, h for Fe- and Co-based coordination polymers, respectively); details at low temperatures are shown in the insets. Data are illustrated as empty points, and the lines were obtained from the simultaneous fit in PHI of the magnetization and χ*T* curves (see parameters in Table 2). Paramagnetic Brillouin curves at 2 K, calculated for the respective spins and *g*_eff_ from Table 2, are provided as dashed lines for comparison with measured magnetization curves. The numbers next to the curves in parts (b) and (f) mark the corresponding temperatures (labels for 3 and 4 K are not shown).

**Figure 7:**
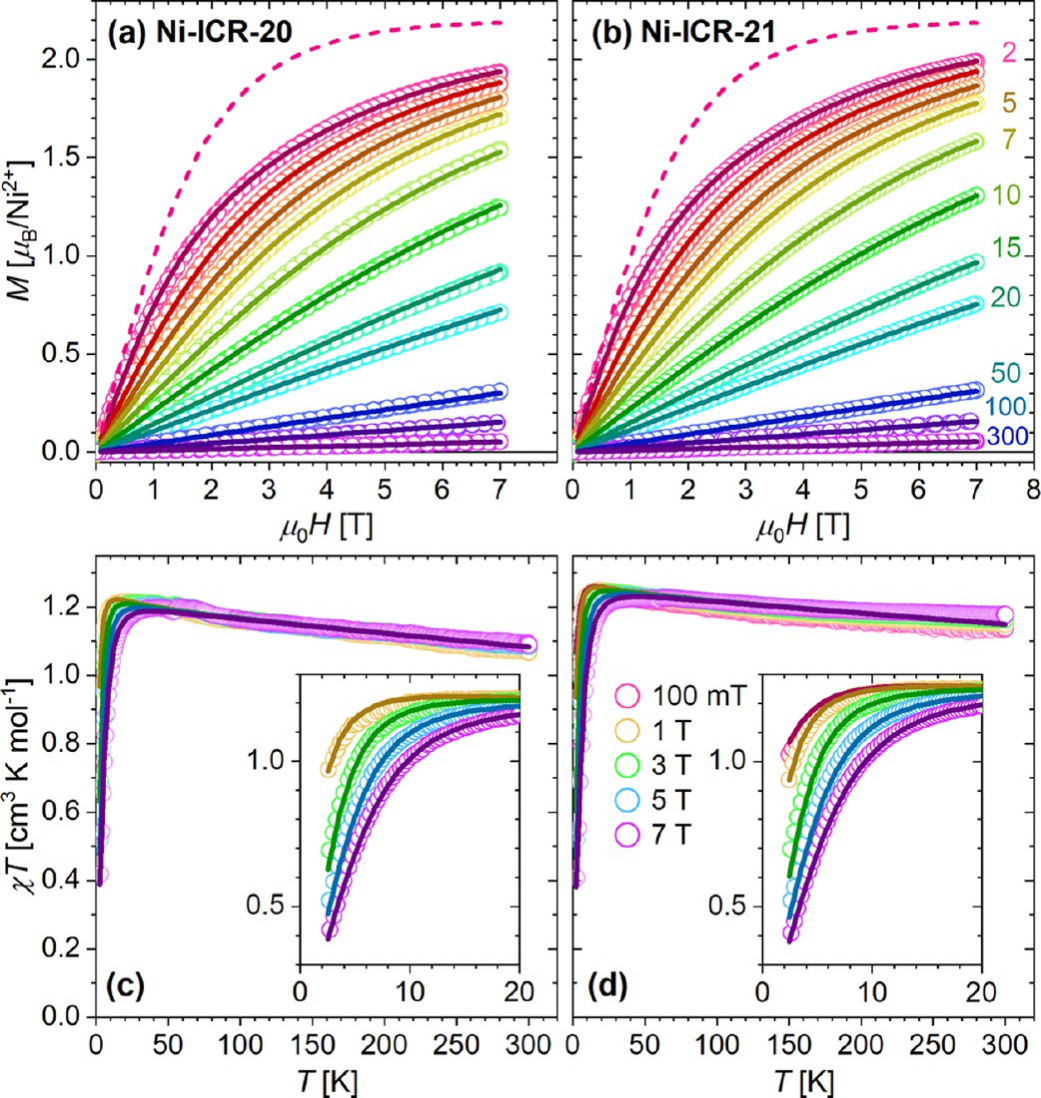
Field-dependent magnetization curves at various temperatures (a, b) and the product of DC susceptibility and temperature, χ*T*, at various fields (c, d) for Ni-based coordination polymers; details at low temperatures are shown in the insets. The numbers next to the magnetization curves in part (b) mark the corresponding temperatures. Paramagnetic Brillouin curves at 2 K, calculated for the respective spins and *g*_eff_ from Table 2, are provided as dashed lines for comparison. The data are shown as empty points; the lines were obtained from the simultaneous fit in PHI of the magnetization and χ*T* curves (see parameters in Table 2).

The χ*T* plots demonstrate a significant decrease down to low temperatures, which can be attributed to the combined effect of the exchange interactions and zero-field splitting (ZFS) of the individual ions. Actually, the temperature at which χ*T* departs from the constant – paramagnetic – value provides a rough estimate of the magnitude of the leading ZFS parameter.^47^ For a basic estimate of the magnetic parameters, we fitted the reciprocal DC susceptibility (see section 6.1 in the Supporting Information) with the Curie–Weiss law (1) in the fully paramagnetic state at higher temperatures of 100–295 K (150–295 K for Co^2+^ coordination polymers).

1 $$\chi^{-1}\left(T\right)={\left(\frac{C}{T-\theta_{\mathrm{CW}}}+\chi_{\mathrm{TI}}\right)}^{-1}$$

Here, *C* is the Curie constant, θ_CW_ is the Curie–Weiss temperature, and χ_TI_ is the temperature-independent susceptibility combining the remaining diamagnetic contribution and temperature-independent paramagnetism. The effective moment per metal ion, expressed as the number of Bohr magnetons μ_B_, can be calculated as $\mu_{\mathrm{eff}}=\sqrt{3k_{B}C/\left(N_{A}\mu_{B}^{2}\right)}$ , with *k*_B_ and *N*_A_ representing the Boltzmann constant and Avogadro number. Alternatively, the effective *g*-factor, which points out the departure from the spin-only behavior, can be expressed as $g_{\mathrm{eff}}=\mu_{\mathrm{eff}}/\sqrt{S\left(S+1\right)}$ , considering the spin *S* of the ions. The results from the Curie–Weiss fits are summarized in Table S5 in the SI, while μ_eff_ and *g*_eff_ are also provided in Table 2.

**Table 2:** Parameters of the Spin Hamiltonian and Other Magnetic Properties of ICR-20 and ICR-21, Obtained from the Simultaneous Fit of *M*(*H*) and χ*T*(*T*) Curves in PHI with a Single-Ion Model and from the Curie–Weiss Fit (CW, Equation 1)­^a^

	*D* [cm^–1^]	|*E*|/*D*	*g*_1_	*g*_2_	*g*_3_	*g*_iso_	*zJ* [cm^–1^]	μ_eff,CW_ [μ_B_]	*g*_eff,CW_	*M*(2 K, 7 T) [μ_B_]
Fe-ICR-20	5.9	0.18	1.54	2.05	2.61	2.07	–5	5.23(2)	2.14(1)	3.16
Co-ICR-20	62	0.28	2.44	2.44	2.85	2.57	63	5.4(1)	2.76(6)	2.15
Ni-ICR-20	–8.5	0.07	2.04	2.04	2.47	2.19	70	3.16(2)	2.24(1)	1.94
Fe-ICR-21	5.0	0.28	1.79	2.03	2.42	2.08	19	5.18(1)	2.12(1)	3.07
Co-ICR-21	60	0.29	2.40	2.40	2.70	2.50	24	5.11(3)	2.64(2)	2.11
Ni-ICR-21	–8.0	0.09	2.03	2.03	2.58	2.21	9	3.10(1)	2.19(1)	1.99

a The parameter designations: *D* and |*E*|/*D* – axial and rhombic ZFS parameters, *g*_i_ – principal values of the *g*-tensor (numbered in ascending order), *g*_iso_ = (*g*_1_ + *g*_2_ *+ g*_3_)/3, *zJ* – mean-field interaction with other magnetic ions; μ_eff,CW_ and *g*_eff,CW_ – effective moment and g-factor obtained from the Curie–Weiss fit, average values summarized over fits in applied fields 1–7 T, with the sample standard deviation in the brackets. *M*(2 K,7 T) – experimental magnetization at 2 K and 7 T.

The effective moment falls within the usually observed range for the respective ions in the high-spin state and approximately octahedral coordination, i.e., 5.1–5.7 μ_B_ for Fe^2+^, 2.9–3.3 μ_B_ for Ni^2+^, and is slightly larger than the typical range of 4.3–5.2 μ_B_ for Co^2+^.^48^ The enhanced μ_eff_, as well as *g*_eff_ > 2, suggest an orbital contribution arising from admixed excited states (cf. the theoretical spin-only moment μ_s‑o_ of 4.90, 2.83, and 3.87 μ_B_ for *S* = 2, 1, and 3/2, respectively). Note the particularly large orbital magnetic moment 1.2–1.4 μ_B_ in the Co^2+^ coordination polymers, considering the distorted octahedral coordination.

The magnetization curves exhibited no hysteresis at temperatures down to 2 K (for full curves and additional temperatures, see Figures S23, S24, and S25 in the Supporting Information). The moment at 2 K and 7 T is provided in Table 2. The calculated Brillouin curves, corresponding to isolated metal ions in the high-spin state, emphasize the reduction of magnetic moment per metal atom at low temperatures due to single-ion anisotropy, especially in the case of Co^2+^ coordination polymers. At higher temperatures, where χ*T* reaches the plateau indicating purely paramagnetic behavior (Figures 6 and 7), magnetization follows Brillouin curves with respective *g*_eff_.

In both ICR-20 and ICR-21 structures, the chains form rather isolated one-dimensional magnetic units, in which each metal ion interacts with its two neighbors through phosphonate or phosphinate groups (see Figures 1 and 2). Due to the minimal overlap between the p-orbital tails of oxygen in neighboring metal coordination spheres, the through-space interaction is very weak. However, vacant 3d-orbitals of the P atom can participate limitedly in spin density transfer,^49^ enabling the supersuperexchange interaction through the M–O–P–O–M pathway. The distances between the second nearest neighbors within the chain and between metal atoms in the closest chains are comparable, extending to roughly 9.5 Å, and the corresponding dipole–dipole interactions are even one or more orders of magnitude weaker than the supersuperexchange.

Considering the weak interactions between the metal centers, magnetic properties are dominated by the single-ion anisotropy of the individual metal ions. Therefore, it is possible to model the system as composed of individual magnetic centers instead of a one-dimensional (1D) chain and include the interaction with other magnetic ions through the mean-field interaction. Both magnetization and susceptibility data sets were fitted simultaneously for each sample using the PHI software,^40^ combining the effects of the crystal field, mean-field, and the Zeeman interaction, see Figures 6 and 7. The resulting parameters are summarized in Table 2, and the respective Hamiltonian is discussed in the SI in more detail. Based on the fitted parameters obtained from various initial conditions, the uncertainty in these parameters is conservatively estimated to be approximately 5%. The large fitted data sets enabled us to achieve low correlation among the fitted parameters in general due to their differing temperature and field dependencies. The largest correlation was observed between individual principal components of the *g*-tensor; their relative order is therefore challenging to determine unequivocally, although their anisotropy and average values remain meaningful. To reduce this correlation, two components were typically linked. However, this approach resulted in a significantly poorer fit for Fe-based CPs.

The ZFS in both Fe-ICR-20 and Fe-ICR-21 leads to a *m_S_* = 0 ground state and easy-plane-like anisotropy. In the case of Co^2+^ coordination polymers, the states form *m* = ± 1/2 and ± 3/2 Kramers doublets. The large splitting between them manifests by a magnetization plateau at ∼2.1 μ_B_/Co^2+^ above roughly 3 T at 2 K,^50^ and complicates the unambiguous determination of the ground state from the magnetic data themselves. However, a slightly better fit was obtained with D > 0, leading to the ground Kramers doublet *m* = ± 1/2, i.e., easy-plane-like anisotropy. Actually, easy-plane anisotropy was also observed based on high-field EPR for Co^2+^ in a similar coordination environment CoO_4_N_2_ in [Co­(2,6-dfba)_2_(bpp)_2_(H_2_O)_2_]_*n*_ and [Co­(2,6-dfba)_2_(bpe)_2_(H_2_O)_2_]_*n*_ (2,6-Hdfba = 2,6-difluorobenzoic acid, bpp = 1,3-bis­(4-pyridyl)­propane and bpe = 1,2-bis­(4-pyridyl)­ethylene; *D* = 53.19 and 65.67 cm^–1^, |*E*|/*D* ∼ 0.21 and 0.064, respectively).^51^ Finally, D < 0 in the case of Ni-ICR-20 and Ni-ICR-21 is related to a mixed *m*_S_ = ± 1 ground state and eas*y*-axis anisotropy, most probably along the approximate N1–Ni–N2 direction, with *D* and *g*_iso_ values comparable, e.g., to Ni^2+^ in NiO_4_N_2_ polyhedra in Ni­(H_2_O)_2_(acetate)_2_(4-picoline)_2_ (*g* = 2.20(1), *D* = −3.96(2) cm^–1^ and *E/D* = −0.23).^47^ The shape of the coordination sphere of Ni^2+^ corresponds to an almost regular octahedron (see bond lengths for Ni-ICR-20 in Table S3), which leads to the small rhombicity |*E*|/*D*. The average value of *g*-factor *g*_iso_ agrees well with *g*_eff,CW_ from the Curie–Weiss fit for all materials. The parameter *zJ* suggests an insignificant, mostly ferromagnetic mean-field interaction with more distant metal atoms.

An analogous fit was attempted for exchange-coupled dimers (or trimers) to obtain a rough estimate of the supersuperexchange interaction strength in the chain. The fitted values of the exchange interaction constant *J* typically fell within the range ∼0.03–0.1 cm^–1^, being antiferromagnetic for both Fe- and Co-based CPs and ferromagnetic for Ni-based materials. Interestingly, introducing a weak antiferromagnetic interaction is necessary to reproduce a correct slope of the χ*T* fit for Co-ICR-21 at the lowest field. Nevertheless, *zJ* and possible *J* represent minor corrections, and their impact is obscured by the dominant effect of the single-ion anisotropy.

### ^57^Fe Mössbauer Spectroscopy

Ferrous ions, with their even number of electrons, are not subject to Kramers’ theorem and often exhibit a singlet ground state, as also demonstrated above for Fe-ICR-20 and Fe-ICR-21. This significantly complicates electron spin resonance analysis, making Mössbauer spectroscopy an indispensable tool for probing such systems.

Although the unpaired electrons in a paramagnetic ion usually give rise to a large magnetic hyperfine field at the nucleus, the Mössbauer spectra of most paramagnetic materials consist of doublets or singlets because the paramagnetic relaxation time is so short that the nucleus only experiences the average value of the magnetic hyperfine field (the thermal average of the total spin ⟨*S*⟩ is zero). At room temperature, the ^57^Fe Mössbauer spectra of Fe-ICR-20 and Fe-ICR-21 consist of two doublets *D*_1_ and *D*_2_ with close values of isomer shift (*IS*) of ∼1.2 mm s^–1^ but with markedly different quadrupole splitting (*QS*) of ∼2.5 mm s^–1^ and ∼2.20 mm s^–1^, respectively (see Figure 8a,d and Table 3). These parameters are characteristic of the high-spin Fe^2+^ (*S* = 2) ions in the paramagnetic state.^52–54^

**Figure 8:**
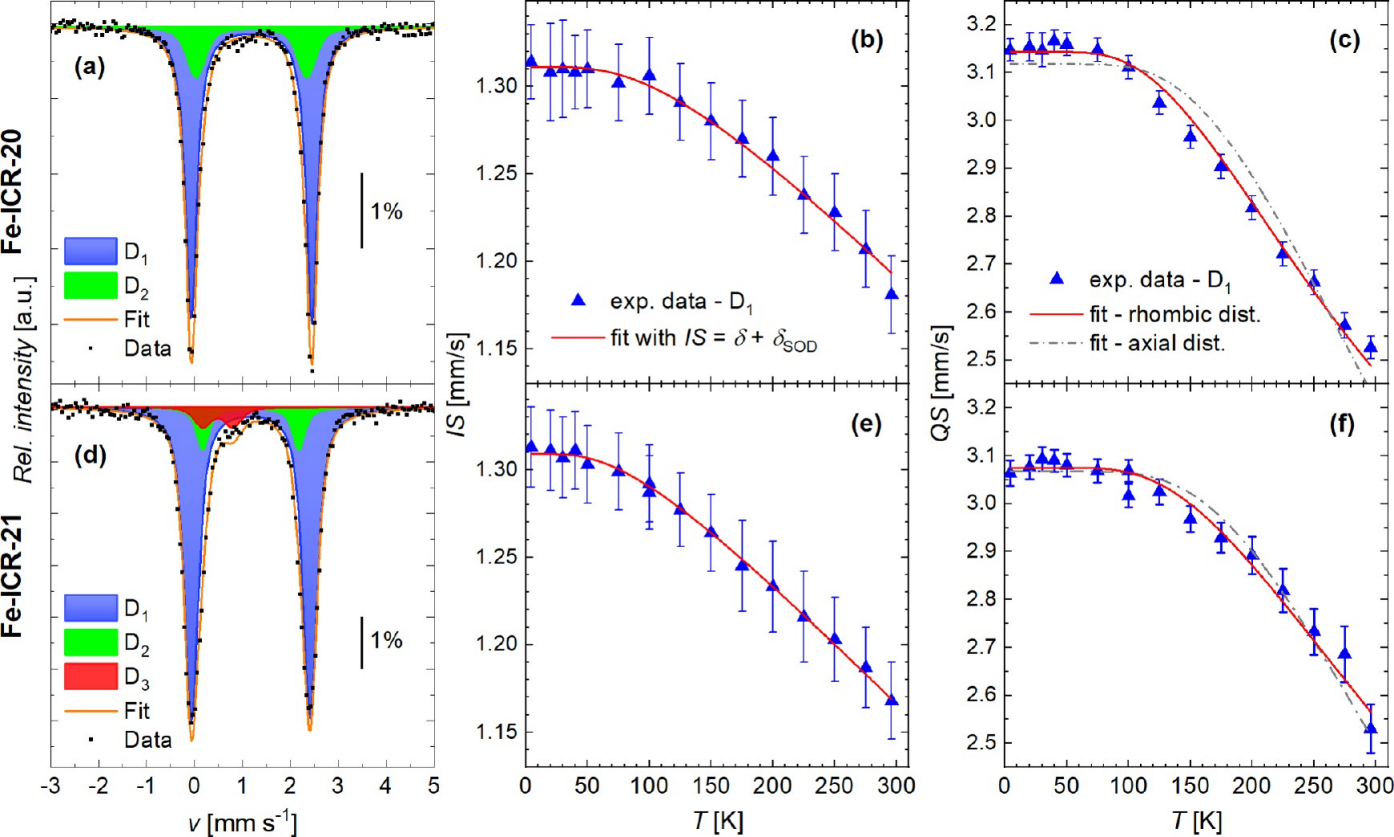
Room-temperature Mössbauer spectra and temperature dependence of *IS* and *QS* of Fe-ICR-20 (a–c) and Fe-ICR-21­(d–f). The spectra in parts (a, d) were acquired in a zero applied field at 295 K, and the vertical line shows the effect of the spectra. In parts (b, e), IS was fitted with eq (7,8) in the SI, while in parts (c, f), both fits of *QS*, considering axial or combined axial and rhombic distortions (see SI), are shown for comparison. The corresponding spectra were measured in a zero external magnetic field; only the temperature dependence of the main component (*D*_1_) is shown.

**Table 3:**
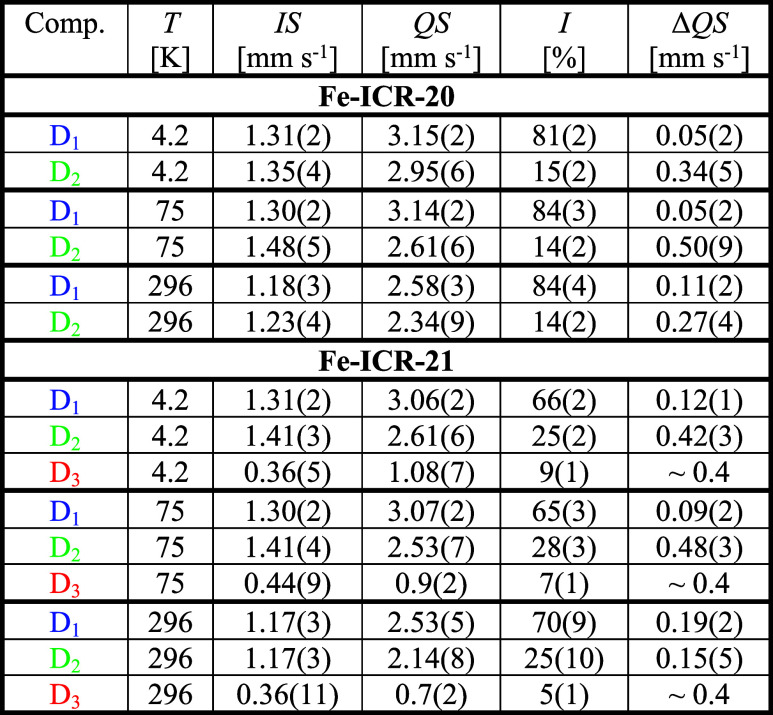
Hyperfine Parameters Determined from the ^57^Fe Mössbauer Spectra of the Fe-ICR-20 and Fe-ICR-21 Samples^a^

a The parameter designations: *IS* – isomer shift, *QS* – quadrupole splitting, *I* – intensity (relative area), Δ*QS* – distribution width of quadrupole splitting, and the line width was fixed to 0.27 mm s^–1^.

The first doublet *D*_1_ with integral intensity of ∼84% (Fe-ICR-20) and ∼70% (Fe-ICR-21) corresponds to Fe^2+^ ions in the distorted octahedral surrounding FeO_4_N_2_ (see Figures 1,2). The second doublet *D*_2_ with an intensity of ∼14% and ∼25%, respectively, and the smaller *QS* can be attributed to Fe^2+^ ions which are affected by the missing water molecules in the voids of the crystal structure,^55^ and possibly even in the coordination sphere. This assignment is based on the comparison of data collected at ambient conditions and after exposure to vacuum at room temperature (see Section 8.1, Figures S32 – S34 in the Supporting Information for more details). Upon evacuation, the intensity of *D*_2_ substantially increased and then dropped to the original value after exposure to air. The release of water under vacuum was less significant for Fe-ICR-21, in accordance with the thermogravimetric analysis. Importantly, the good agreement between room-temperature spectra before and after all of the measurements demonstrates the reversibility of the (de)­hydration process. This is in agreement with the results of the water adsorption experiment.

The D_3_ doublet in the Fe-ICR-21 sample with *IS* ∼ 0.36 mm s^–1^, *QS ∼* 0.7 mm s^–1^, and integral intensity *I* ∼ 5% probably originates from larger disturbances in the immediate vicinity of the Fe^2+^ ion or the minor impurity detected by PXRD; the value of the isomer shift and quadrupole splitting may correspond to Fe^3+^ or Fe^2+^ in the lower spin state *S* < 2. This component, missing in Fe-ICR-20, slightly increases after evacuation, at the expense of the intensity of *D*_1_, and may hint at certain aging of the evacuated sample.

In a zero external magnetic field, the shape of the Mössbauer spectra does not change with increasing temperature, while both *QS* and *IS* decrease upon heating (see Figure 8 and Table 3).

Isomer shift decreases due to the second-order Doppler effect (SOD), caused by the thermal motion of the emitting/absorbing nuclei. The temperature dependence of the experimental isomer shift can be described by *IS*(*T*) = δ + δ_SOD_(*T*), where δ and δ_SOD_ are the chemical isomer shift (see eq (21) in the SI) and the second-order Doppler shift. Using the Debye model to describe SOD, one can determine the Debye (Mössbauer) temperature θ_D_ specific for the ^57^Fe nuclei in their environment (see Section 7.2 in the SI).^53^ The fits of the temperature-dependent *IS* provide δ = 1.43(1) mm s^–1^, θ_D_ = 450(20) K, and δ = 1.40(1) mm s^–1^, θ_D_ = 323(9) K for Fe-ICR-20 and Fe-ICR-21, respectively, see Figure 8b,e. The value of the isomer shift at liquid-helium temperature *IS* = 1.31 mm s^–1^ is in good agreement with other compounds containing octahedrally coordinated Fe^2+^ ions in the high-spin state with rather high ionic character of the bonding, e.g., with *IS* = 1.35 mm s^–1^ for triphylite LiFePO_4_ ^54^ or 1.31 and 1.34 mm s^–1^ for vivianite Fe_3_(PO_4_)·8H_2_O.^56^

The decrease in *QS* with rising temperature reflects temperature-dependent populations of the split *d*_ε_ (*t*_2g_ set) energy levels. The fit of the temperature dependence of *QS*, based on a simple model proposed by Ingalls, neglecting the spin–orbit coupling,^57^ is shown in Figure 8c,f (for the formulas and more details, see Section 8.3 in the SI). The fits confirmed the necessity to consider both axial and rhombic distortion of the Fe^2+^ octahedral coordination sphere, which completely lifts the degeneracy of the *d*_ε_ levels (in contrast to a purely axial distortion, which splits the *d*_ε_ levels to a doubly degenerate level and a nondegenerate one). The corresponding energy differences Δ*E*_1_ and Δ*E*_2_ between the lowest level and the higher ones can be estimated from the model, and the fits of *QS*(*T*) provide Δ*E*_1_ ∼ 370 cm^–1^, Δ*E*_2_ ∼ 1350 cm^–1^ and Δ*E*_1_ ∼ 430 cm^–1^, Δ*E*_2_ ∼ 1210 cm^–1^ for Fe-ICR-20 and Fe-ICR-21, respectively (Δ*E*_2_ represents a lower estimate above which the shape of the fitted curve does not change). These experimental estimates are in good agreement, for example, with the values Δ*E*_1_∼ 360 cm^–1^, Δ*E*_2_ ∼ 1680 cm^–1^ reported by Ingalls for FeSO_4_.^57^ The higher Δ*E*_1_ of Fe-ICR-21 (and a slower decrease of *QS* with rising temperature) are consistent with the larger deformation of the coordination environment of Fe and higher rhombicity |*E*|/*D*, as observed for all of the ICR-21 CPs.

When the paramagnetic relaxation time τ is on the same order as the time scale of Mössbauer spectroscopy τ_M_ ∼ 140 ns, the spectra broaden, and for τ ≫ τ_M_ (e.g., in the case of a very slow spin–spin relaxation), the magnetic hyperfine interaction gives rise to magnetically split spectra. In special cases, for example, in low-temperature studies of magnetically dilute samples, the magnetic hyperfine structure is revealed only upon application of the magnetic field, while the zero-field spectra are split merely by the electric quadrupole interaction. The magnetic hyperfine structure is highly sensitive to the applied field, and the changes in the internal field manifested in the spectra can exceed the applied field by an order of magnitude. Both Fe-ICR-20 and Fe-ICR-21 exhibit this behavior at low temperatures; see Figures 9 and 10 for the comparison of spectra acquired at *B*_ext_ = 0 and 6 T at selected temperatures and Section 8.6 in the SI for spectra collected at various applied fields at 4.2 K. A similar effect was observed, e.g., in Fe^2+^-doped carbonates (ACO_3_ with A = Ca, Cd),^58^ anapaite Ca_2_Fe­(PO_4_)_2_·4H_2_O containing [Fe­(H_2_O)_4_(PO_4_)_2_]^2+^ cations,^59^ or hexafluorosilicate FeSiF_6_·6H_2_O with [Fe­(H_2_O)_6_]^2+^ cations.^60^

**Figure 9:**
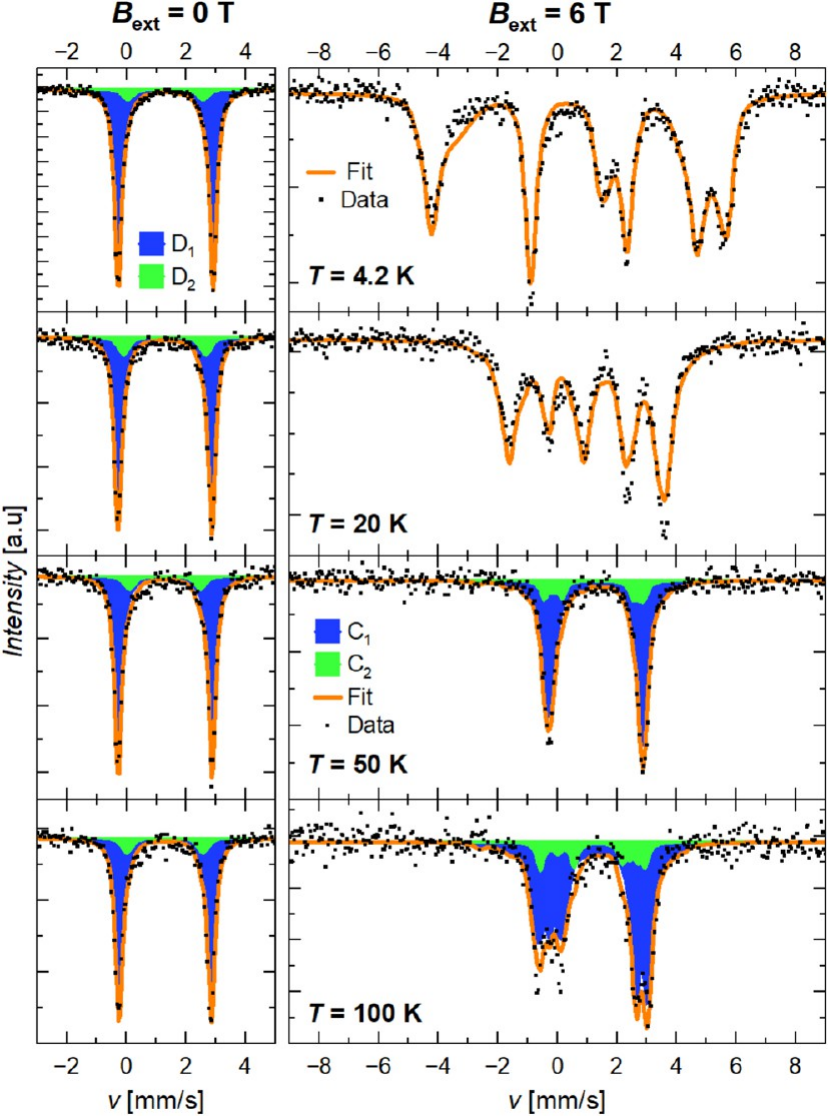
Mössbauer spectra of Fe-ICR-20 at selected temperatures in a zero external magnetic field (left) and in the field *B*_ext_ = 6 T (right). The in-field spectra at 4.2 and 20 K were fitted with the PHS model with a single component, while those at 50 and 100 K were fitted with the mixed-interaction Static Hamiltonian model. The major ticks at the *y*-axis correspond to 2% (0.5% in the 100 K/6 T spectrum). The *C_i_* components in the in-field spectra correspond to the *D_i_* doublets in the zero field.

**Figure 10:**
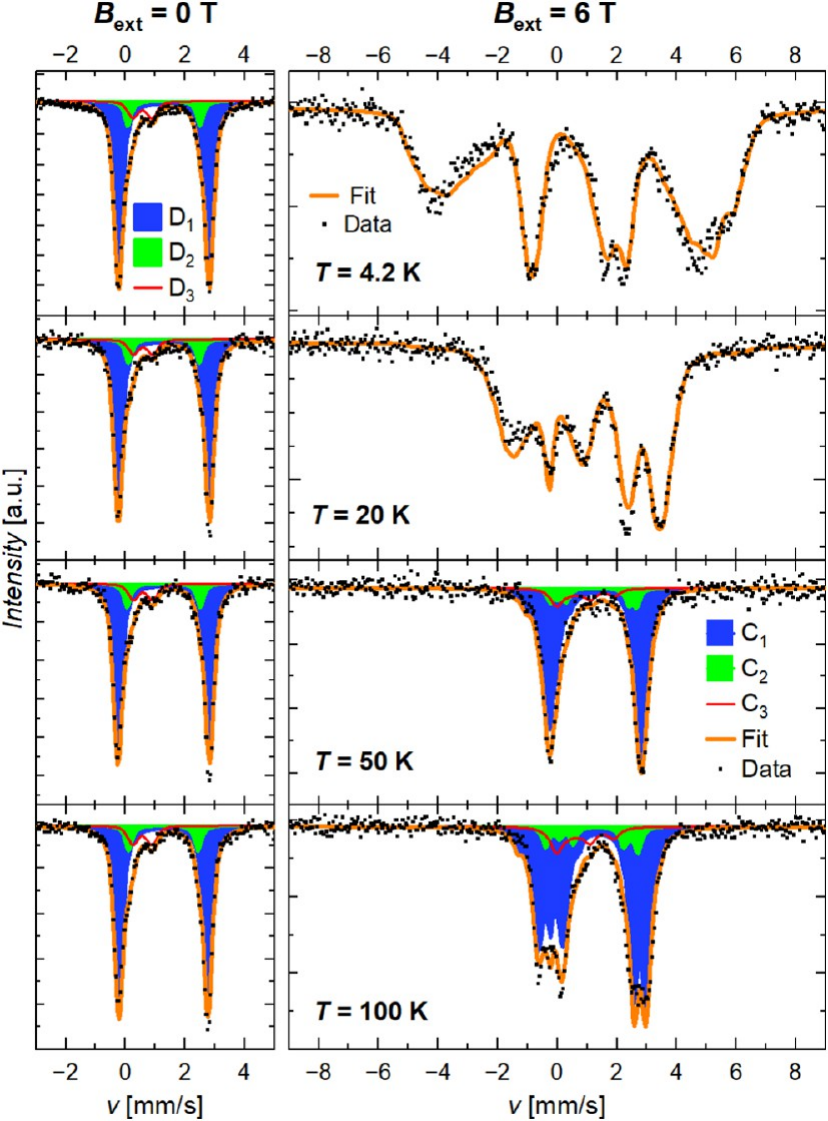
Mössbauer spectra of Fe-ICR-21 at selected temperatures in a zero external magnetic field (left) and in the field *B*_ext_ = 6 T (right). The in-field spectra at 4.2 and 20 K were fitted with the PHS model, while those at 50 and 100 K were fitted with the mixed-interaction Static Hamiltonian model. The major ticks at the *y*-axis correspond to 2%. The *C_i_* components in the in-field spectra correspond to the *D_i_* doublets in the zero field.

While the zero-field spectra can be analyzed with a simple model with doublet components (see above), the shape of the in-field spectra crucially depends on the detailed form of the electronic wave functions, which are determined by the spin Hamiltonian.^42,53,61–65^ Among others, the spin Hamiltonian involves an interaction with the crystal field. The electronic spin Hamiltonian is extended by an appropriate nuclear Hamiltonian including the electric quadrupole interaction, characterizing the interaction of the nuclear quadrupole moment with the electric-field gradient (EFG) tensor V̿ , and magnetic hyperfine coupling, connecting the electronic and nuclear spins through the hyperfine coupling tensor A̿ ; see Section 8.4 in the SI for a more detailed overview. Employing an effective Hamiltonian approach, we can estimate ZFS parameters, as well as principal components of the V̿ and A̿ tensors.

The in-field spectra at temperatures up to 20 K were analyzed in the Paramagnetic Hyperfine Structure (PHS) model implemented in MossWinn,^61^ which assumes a slow electronic relaxation and decoupled electronic and nuclear states. Only a single component was considered, and the obtained parameters were ascribed to the main component (“*D*_1_”). For Fe-ICR-20, the simultaneous fit of the spectra at 4.2–20 K acquired at *B*_ext_ = 6 T provided the ZFS parameters *D* = 6.04(1) cm^–1^, *E*/*D* = 0.20(1), and principal g-values of 2.07, 2.13, and 2.15 for *g*_*xx*_, *g*_*yy*_, and *g*_*zz*_, respectively. Analogically, *D* = 4.88(4) cm^–1^, *E*/*D* = 0.28(1), and principal *g*-values of 1.85, 1.85, and 2.46 were obtained for Fe-ICR-21 (here, *g*_*xx*_ = *g*_*yy*_ to stabilize the fit). These values agree remarkably well with those obtained from the fit of the magnetic data. Other parameters are summarized in Section 8.4 in the SI.

The PHS model is limited to low temperatures (*k*_B_ *T* ≲ 2*D*),^63^ where ZFS-split levels have significant thermal population differences. Therefore, the powder-averaged static Hamiltonian model with mixed magnetic hyperfine and (dominating) electric quadrupole interactions was used to analyze the spectra at 6 T at higher temperatures up to 100 K (see Section 8.5 in the SI). To account for the random orientation of the magnetic anisotropy axes with respect to the applied field, the magnetic hyperfine field was further approximated with a distribution. Although this model does not involve ZFS, it still offers valuable insights into the system. The obtained parameters are listed in Table S7.

The strong EFG – as evidenced by the large principal value *V*_*zz*_ of ∼18.8 and 18.3·10^21^ V m^–2^ for Fe-ICR-20 and Fe-ICR-21 and large low-temperature *QS* – is comparable to, e.g., *V*_*zz*_ = 16.7(1)·10^21^ V m^–2^ and *QS* = 3.05(2) mm s^–1^ for LiFePO_4_ with the distorted octahedral environment.^54^ *V*_*zz*_ is positive for both CPs, suggesting an oblate charge distribution around the ^57^Fe nuclei consistent with the easy-plane-like anisotropy detected by the magnetic measurements. The nonzero asymmetry parameter η ∼ 0.26, indicative of a deviation from local axial symmetry, aligns with the structural and magnetic data.

Figure 11 summarizes the temperature dependence of the mean effective magnetic hyperfine field, *B*_eff_ = | ***B***_**hf**_ + ***B***_**ext**_| (i.e., including the intrinsic hyperfine field ***B***_**hf**_ and the applied field of *B*_ext_ = 6 T), of both Fe-ICR-20 and Fe-ICR-21. For the PHS model, the values were calculated as the absolute value of the average of the principal values of the A̿ tensor. The temperature dependence suggests that the hyperfine field ***B***_**hf**_ at the ^57^Fe nuclei is directed opposite to the magnetic moment of the Fe^2+^ ions, and the applied field is compensated at around 50–60 K. At the same time, the large *B*_eff_ ≫ *B*_ext_ can be understood as a local manifestation of the large low-temperature susceptibility of these paramagnetic compounds. The increase in *B*_eff_ at higher temperatures then points to the reversed direction of the vector sum, where ***B***_**hf**_ diminishes with rising temperature (in accordance with the rapidly decreasing magnetic susceptibility) and ***B***_**ext**_ dominates. The anisotropy of the hyperfine field (see *A*_*ii*_ in Table S7) arises from the anisotropy of both the dipolar interaction and the partially restored orbital angular momentum due to spin–orbit coupling (the anisotropic g-tensor).^60^ Slightly lower ⟨*B*_eff_⟩ of Fe-ICR-20 at low temperatures can be ascribed to a more covalent character of bonding,^66^ or possibly to a larger hyperfine field contribution of the orbital moment (cf. larger *g*_eff_, μ_eff_), which adds with an opposite sign to the dominating (negative) Fermi contact interaction.

**Figure 11:**
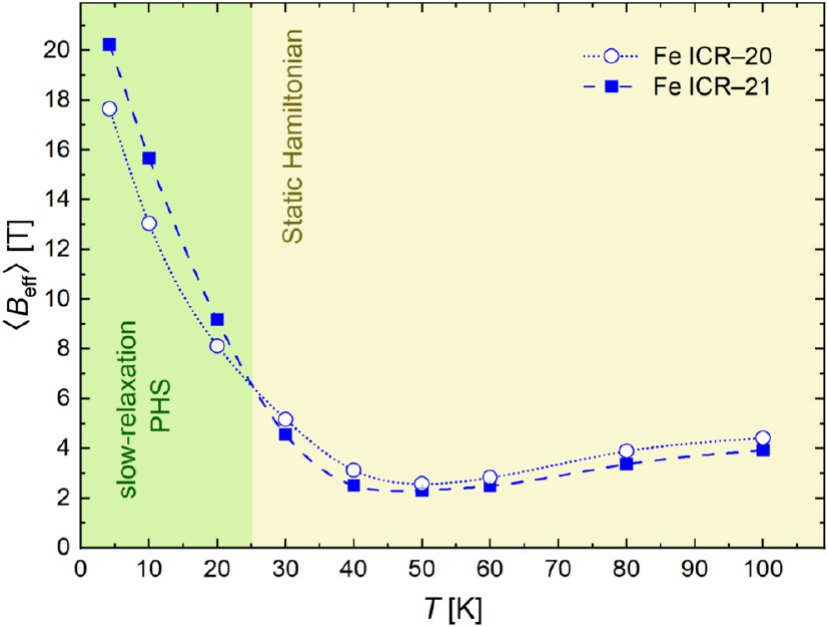
Temperature dependence of the mean effective hyperfine magnetic field at the ^57^Fe nuclei of the main component (*C*_1_) of Fe-ICR-20 and Fe-ICR-21. The corresponding Mössbauer spectra were measured in an external magnetic field *B*_ext_ = 6 T. Measurement errors are smaller than the experimental points and are therefore provided only in Table S7.

### Proton Conductivity

Although the presented materials, ICR-20 and ICR-21, do not reveal any detectable permanent porosity, the respective crystal structures (Figures 1 and 2) contain voids occupied by water molecules. Half of the phosphinate groups in ICR-20 and all phosphonate and phosphinate groups in ICR-21 contain an uncoordinated oxygen atom that is oriented toward the void space and can form strong hydrogen bonds with these molecules. For this reason, ICR-20 and ICR-21 are expected to be proton conductive. In this respect, we have investigated the properties of two selected materials (Co-ICR-20 and Co-ICR-21), and the isostructural frameworks with different metals were not measured because we expect the central atom to have only a negligible effect on the proton conductivity of the material. The proton conductivity was measured at the relative humidity (RH) of 75 and 92% and in the temperature range of 296–304 K. Since the obtained Nyquist plots (Figures S40, S41, S43, and S44) showing the dependence of the imaginary component of impedance (*Z*″) on the real component of impedance (*Z*′) are composed of two semicircles, they were fitted by ((R_1_Q_1_)­(R_2_Q_2_)­Q_3_) equivalent circuit (Figure S39). The proton conductivity values were calculated from both semicircles, and the semicircle that provides increasing conductivity values with higher temperature was attributed to the proton conductivity of the material. The second contribution is probably caused by processes taking place at the grain boundaries. In the temperature range of 296–304 K, the dependence of lnσ on 1/*T* is almost linear (Figure 12), and the activation energy values of 0.54–1.04 eV calculated from the respective Arrhenius plots suggest the vehicle mechanism of proton conduction. At higher temperatures, the proton conductivity displayed a decreasing trend, which is a feature that has been reported for other MOF-based materials, and it is attributed to the loss of proton carriers.^67^ For that reason, the measurements were performed only up to 304 K. The PXRD patterns recorded before and after the proton conductivity measurements (Figures S42 and S45) show that the structure of the materials was preserved.

**Figure 12:**
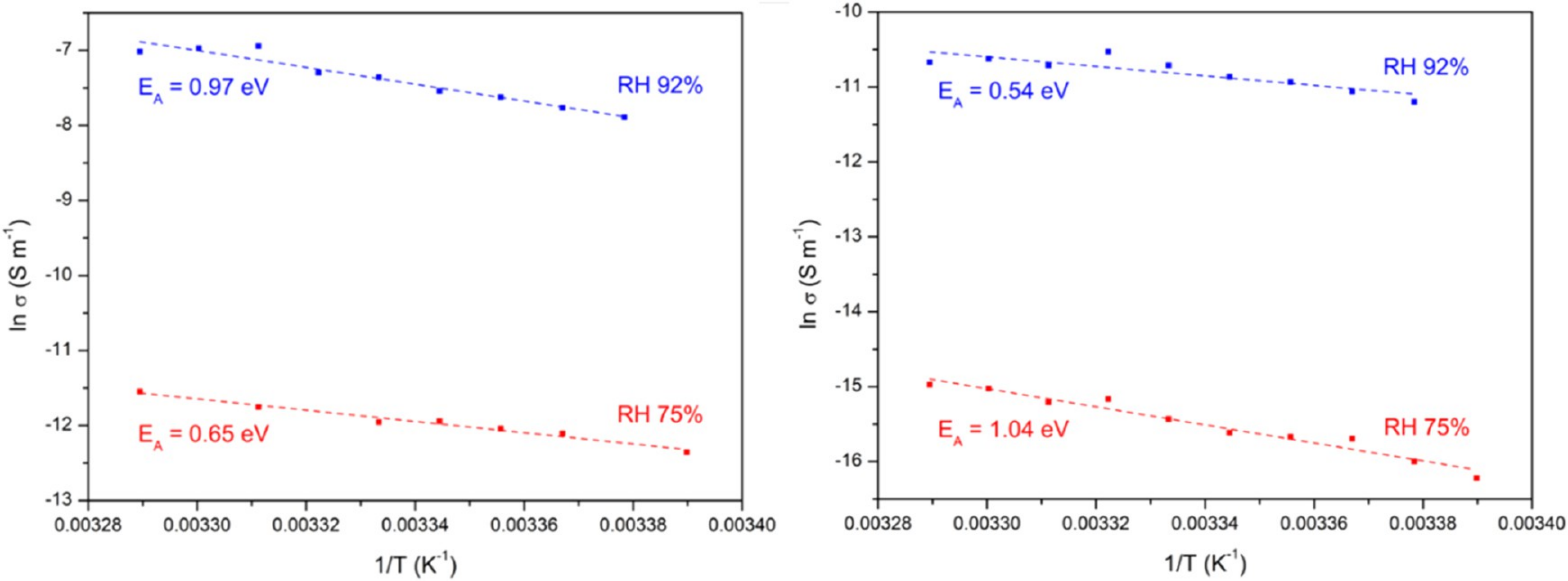
Arrhenius plots for ICR-20 (left) and ICR-21 (right) at different humidities. The estimated uncertainty of the measurement is 10%.

The proton conductivity of Co-ICR-20 at a relative humidity of 75% is on the order of 10^–8^ S cm^–1^ (Table 4). At 92% relative humidity, it increases by 2 orders of magnitude, achieving almost 10^–5^ S cm^–1^ at 302 K. Surprisingly, Co-ICR-21, which is supposed to be more hydrophilic due to the presence of more uncoordinated – OH groups, demonstrates lower values of proton conductivity than Co-ICR-20. This might be due to the particular conduction mechanism suggested for ICR-20 and ICR-21 based on the activation energy. Vehicle mechanism depends on the movement of carrier molecules, in this case water, which can be hindered by strong hydrogen bonds formed with uncoordinated −OH groups. Increasing relative humidity from 75 to 92% improves the proton conductivity by 2 orders of magnitude, similar to Co-ICR-20.

**Table 4:** AC Conductivities of the Co-ICR-20 and Co-ICR-21 Samples Measured at Several Temperatures and Relative Humidities (RHs) of 75 and 92%; the Estimated Uncertainty of the Measurement is 10%

	σ [S cm^–1^]			
	Co-ICR-20		Co-ICR-21	
*T* [K]	RH 75%	RH 92%	RH 75%	RH 92%
295	4.3·10^–8^	-	9.0·10^–10^	-
296	-	3.8·10^–6^	1.1·10^–9^	1.4·10^–7^
297	5.5·10^–8^	4.3·10^–6^	1.5·10^–9^	1.6·10^–7^
298	5.9·10^–8^	4.9·10^–6^	1.6·10^–9^	1.8·10^–7^
299	6.5·10^–8^	5.3·10^–6^	1.7·10^–9^	1.9·10^–7^
300	6.4·10^–8^	6.4·10^–6^	2.0·10^–9^	2.2·10^–7^
301	-	6.8·10^–6^	2.6·10^–9^	2.7·10^–7^
302	7.9·10^–8^	9.7·10^–6^	2.5·10^–9^	2.2·10^–7^
303	-	9.4·10^–6^	3.0·10^–9^	2.4·10^–7^
304	9.7·10^–8^	9.0·10^–6^	3.1·10^–9^	2.3·10^–7^

## Conclusions

We synthesized and characterized two coordination polymers, ICR-20 using a bisphosphinate ligand and ICR-21 using a phosphonate-phosphinate ligand, each with Fe, Co, or Ni as the metal center. This provides the second example of isoreticular design between phosphonates and phosphinates, following phosphonate analogues of a phosphinate MOF previously reported by our group.^15^ The coordination polymers were further studied for their magnetic properties and proton conductivity.

Due to a rather large distance between the metal centers, both inter- and intrachain magnetic interactions are weak, and no long-range magnetic order is established down to 2 K. Magnetic properties are dominated by a strong single-ion anisotropy, which reduces the effective magnetic moment of the ions at low temperatures and leads to an easy-plane magnetic anisotropy for Fe^2+^ and Co^2+^ and an eas*y*-axis anisotropy for the Ni^2+^ coordination polymers. Despite the highly asymmetrical coordination sphere, the observed magnetic moments in all samples indicate a significant orbital contribution, similar in magnitude to that expected for a regular octahedral environment and especially pronounced in the Co^2+^ polymers. In accordance with higher asymmetry of the coordination sphere, the metal centers in ICR-21 typically display a smaller axial zero-field splitting parameter and a higher degree of rhombicity, as confirmed also by ^57^Fe Mössbauer spectroscopy. Zero-field Mössbauer spectra of the Fe^2+^ coordination polymers consisted of two doublets, with the minor one attributed to a missing water molecule near the Fe center. In contrast, low-temperature spectra obtained under an applied field exhibited a more complex paramagnetic hyperfine structure with a highly anisotropic hyperfine coupling tensor, supporting the findings from the magnetic data.

The prepared coordination polymers reveal proton conductivity up to ∼10^–5^ S·cm^–1^ for Co-ICR-20 at 302 K and a relative humidity of 92%. Interestingly, the performance of Co-ICR-20 is better than that of Co-ICR-21, although the structure contains fewer −OH groups. This can be explained by the vehicle conduction mechanism, which depends on the movement of water molecules and can be hindered by strong hydrogen bonds formed with the uncoordinated −OH groups. The achieved proton conductivity is comparable to the majority of reported nonporous phosphonate coordination polymers.^32^

## Supplementary Material



## Notes

PXRD patterns, FTIR spectra, TG analyses, and CHN analyses of the presented coordination polymers; details of the structure determination and lists of selected bond lengths and angles. Brief description and a picture of the ICR-22 phase; details concerning the measurements of magnetic properties and Mössbauer spectra and the accompanying calculations; and Nyquist plots and other details concerning the proton conductivity measurements (PDF)
