# Supplementary material for: Magnetism, Mössbauer Spectroscopy, and Proton Conductivity of Coordination Polymers Based on Phosphonate and Phosphinate Linkers
Source: J Phys Chem C Nanomater Interfaces. 2025 Aug 20;129(35):15850–64. doi: 10.1021/acs.jpcc.5c04260 (PMC12415879; doi:10.1021/acs.jpcc.5c04260)
Supplement: Supplementary file 1 [file jp5c04260_si_001.pdf]

Magnetism, Mössbauer Spectroscopy and Proton Conductivity of Coordination Polymers Based on Phosphonate and Phosphinate Linkers.

Supporting Information

Soňa Ondrušová[a,b], Daniel Bůžek[a], Jan Hynek[a], Tomáš Plecháček[c], Lenka Kubíčková[d,e]

Miroslav Veverka[d], Tomáš Kmječ[d], Matyáš Rozprým[d], Jaroslav Kohout[d], Jan Demel[a], Matouš Kloda[a*]*

[a] Institute of Inorganic Chemistry of the Czech Academy of Sciences, Husinec-Řež 1001, 250 68, Řež, Czech Republic; E-mail: kloda@iic.cas.cz

[b] Department of Inorganic Chemistry, Faculty of Science, Charles University, Hlavova 2030, Prague, 12840, Czech Republic

[c] Center of Materials and Nanotechnologies, Faculty of Chemical Technology, University of Pardubice, Cs. Legii 565, 530 02 Pardubice, Czech Republic

[d] Faculty of Mathematics and Physics, Charles University, V Holešovičkách 2, 180 00 Prague, Czech Republic, E-mail: jaroslav.kohout@matfyz.cuni.cz

[e] Institute of Physics of the Czech Academy of Sciences, Cukrovarnická 10/112, 162 00 Prague, Czech Republic

1. PXRD patterns

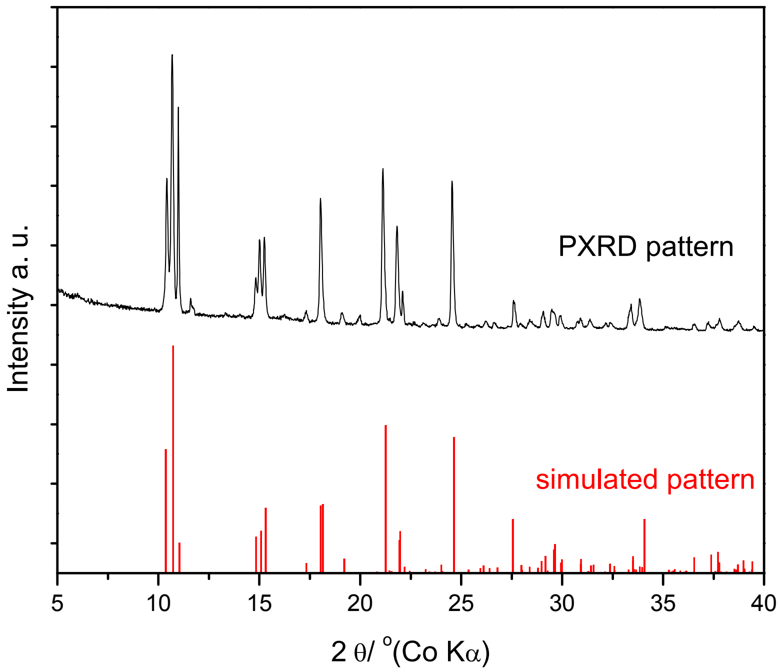

Figure S1. Comparison of measured PXRD pattern of Fe-ICR-20 (black) and simulated pattern from Fe-ICR-20 single crystal measurement (red).

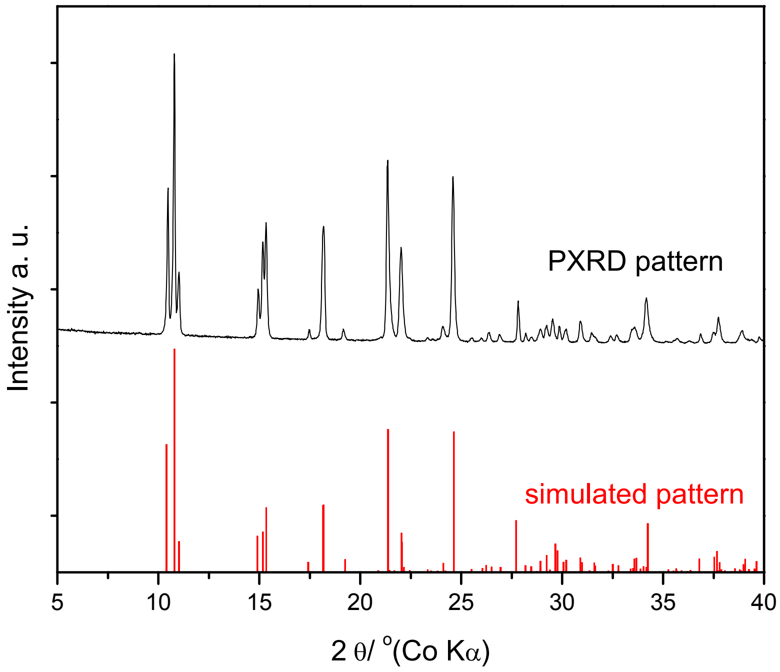

Figure S2. Comparison of measured PXRD pattern of Co-ICR-20 (black) and simulated pattern from Co-ICR-20 single crystal measurement (red).

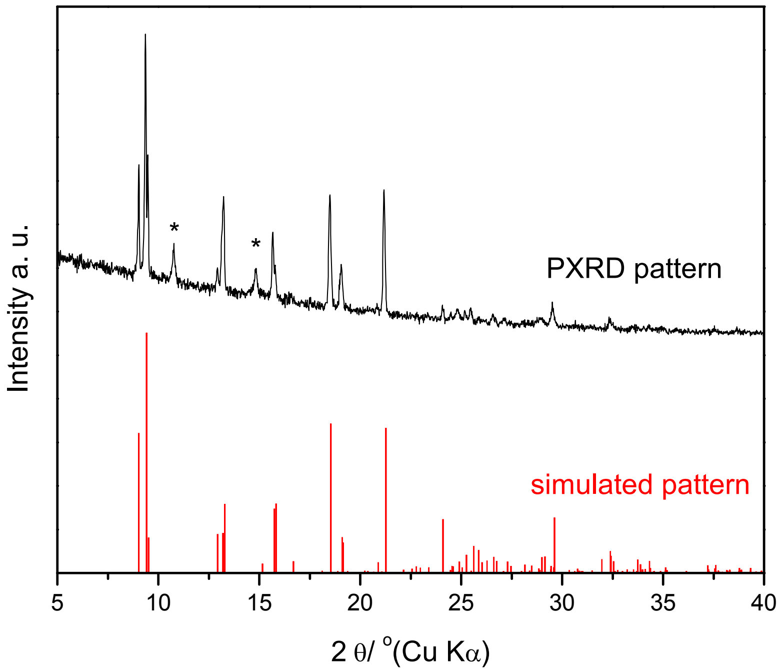

Figure S3. Comparison of measured PXRD pattern of Ni-ICR-20 (black) and simulated pattern from Ni-ICR-20 single crystal measurement (red).

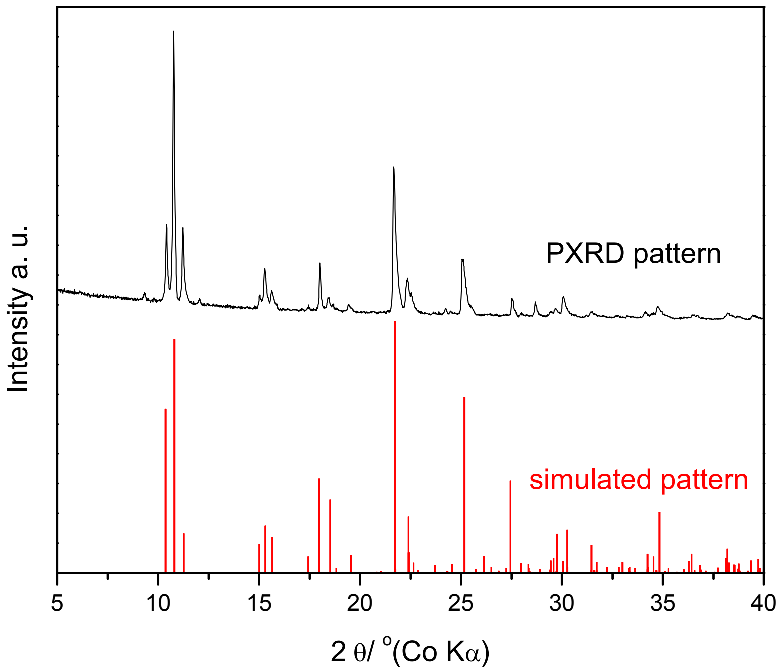

Figure S4. Comparison of measured PXRD pattern of Fe-ICR-21 (black) and simulated pattern from Fe-ICR-21 single crystal measurement (red).

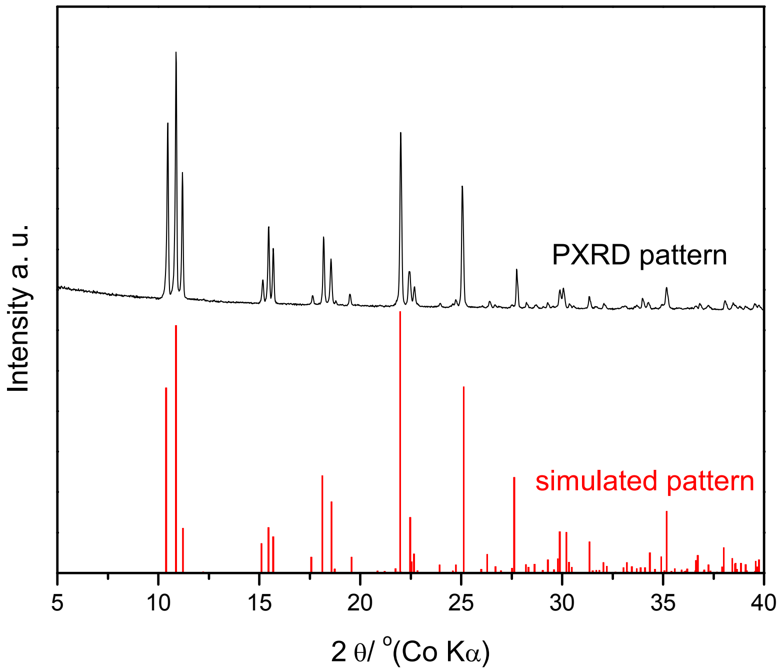

Figure S5. Comparison of measured PXRD pattern of Co-ICR-21 (black) and simulated pattern from Co-ICR-21 single crystal measurement (red).

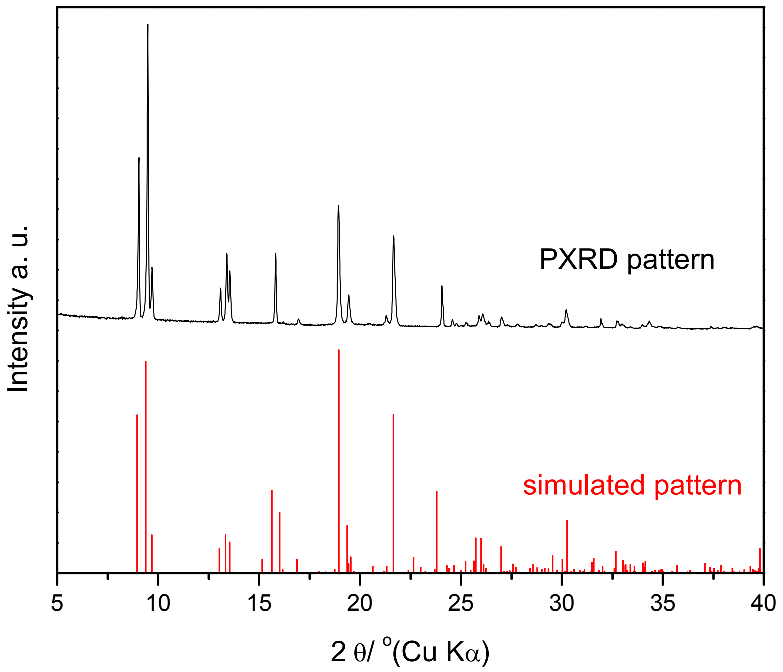

Figure S6. Comparison of measured PXRD pattern of Ni-ICR-21 (black) and simulated pattern from Co-ICR-21 single crystal measurement (red).

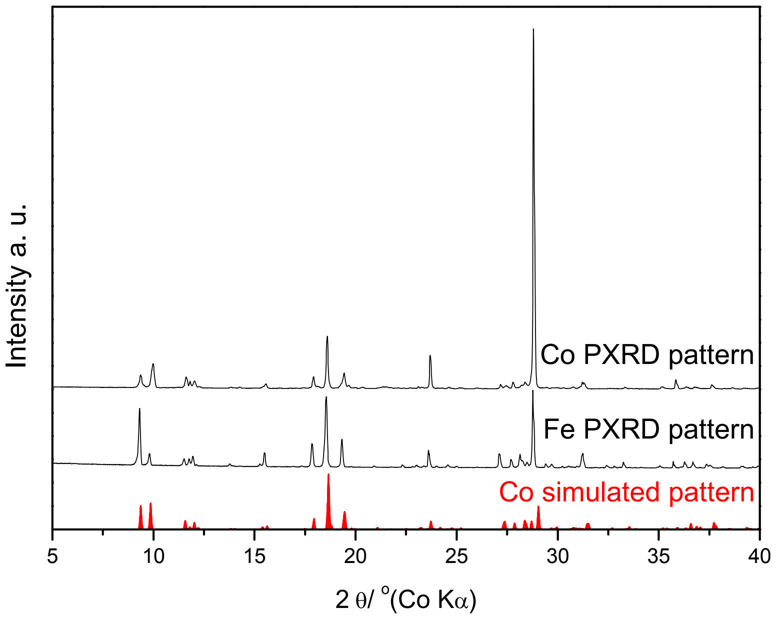

Figure S7. Comparison of measured PXRD pattern of Co and Fe phase formed at higher NaOH content (black) with simulated pattern from single crystal measurement (red).

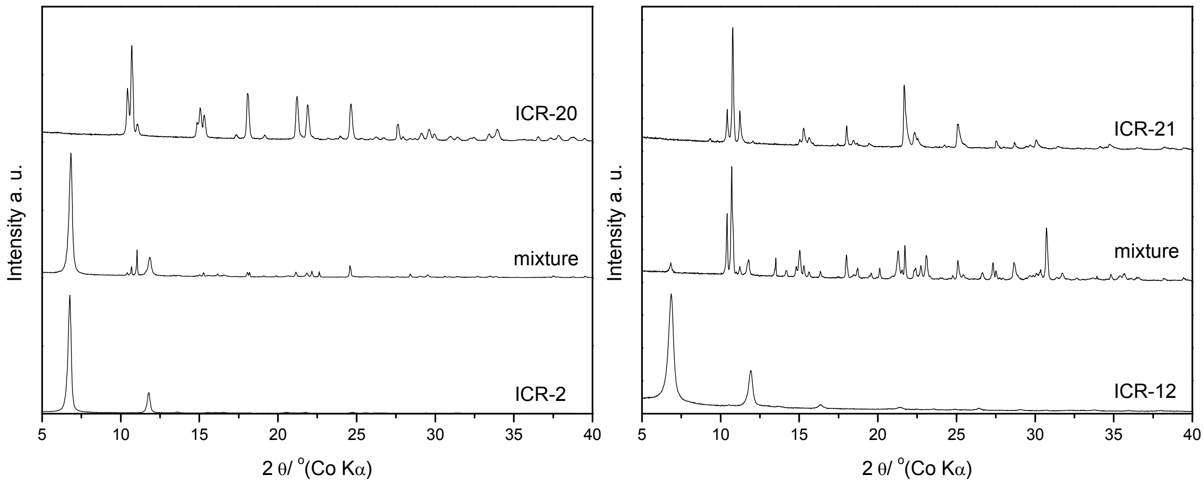

Figure S8. Comparison of PXRD patterns of pure ICR-20 and ICR-21 (top), as-prepared mixtures (middle) and respective Fe^{3+} phases (bottom).

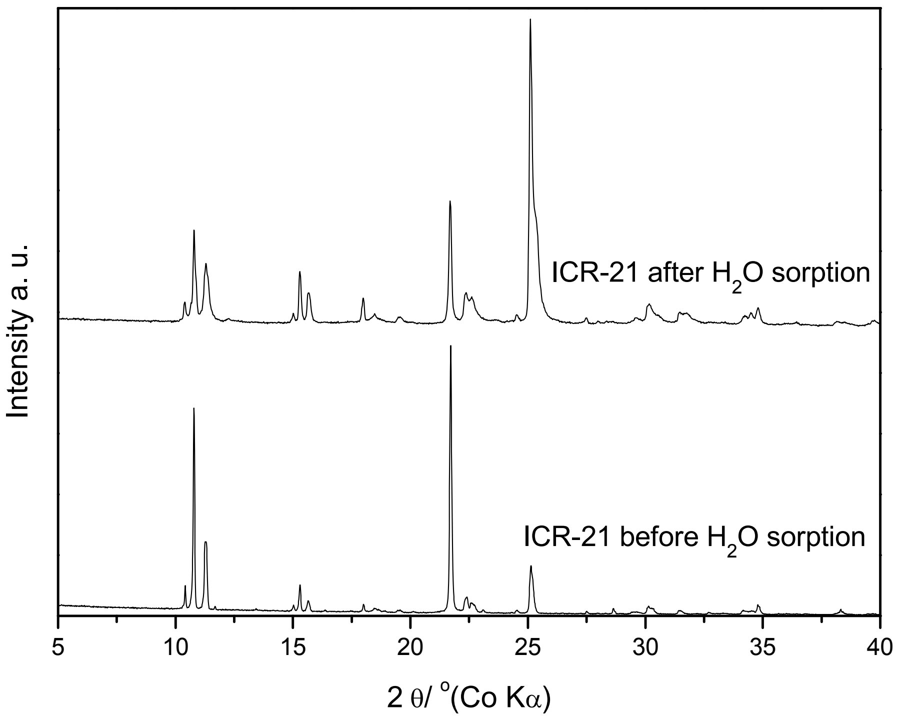

Figure S9. Comparison of PXRD patterns of ICR-21 before and after water adsorption.

2. FTIR spectra

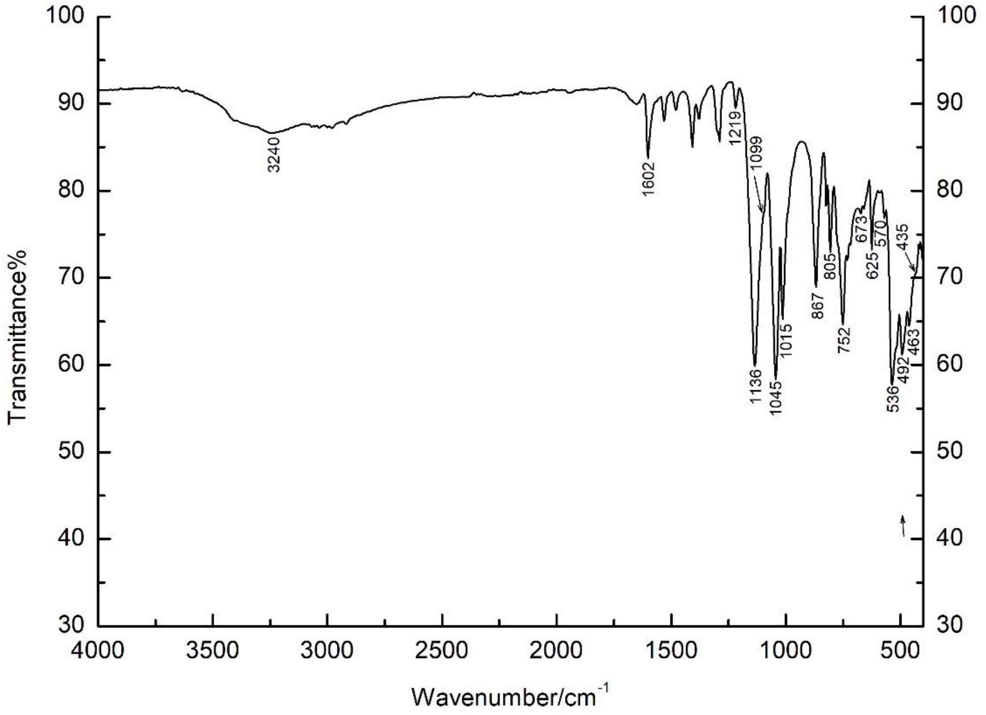

Figure S10. FTIR spectrum of coordination polymer Fe-ICR-20.

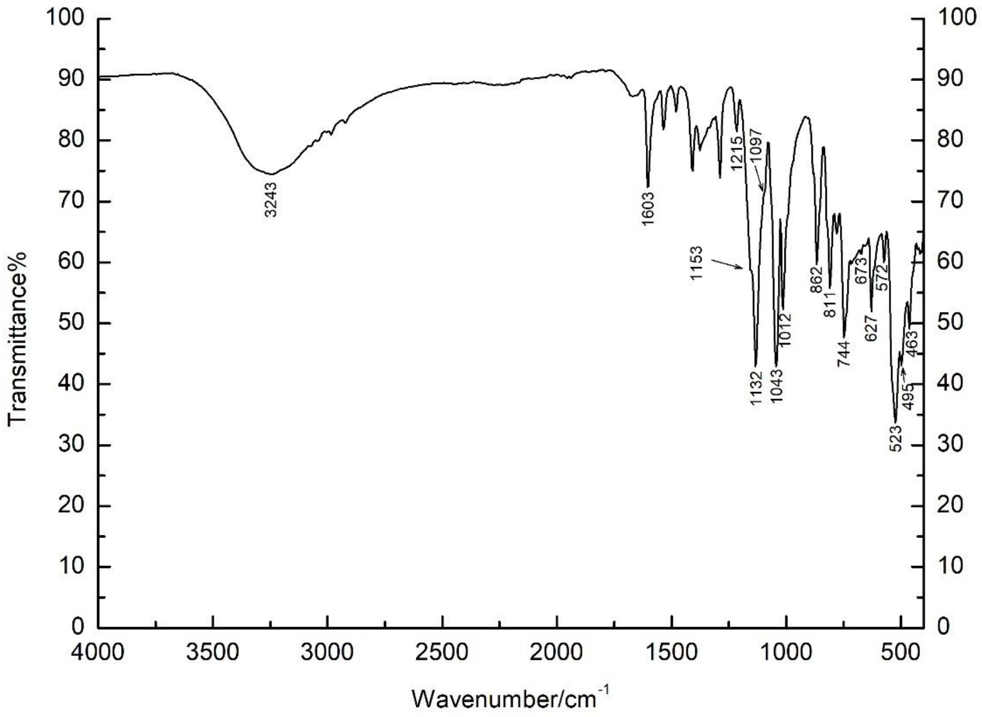

Figure S11. FTIR spectrum of coordination polymer Co-ICR-20.

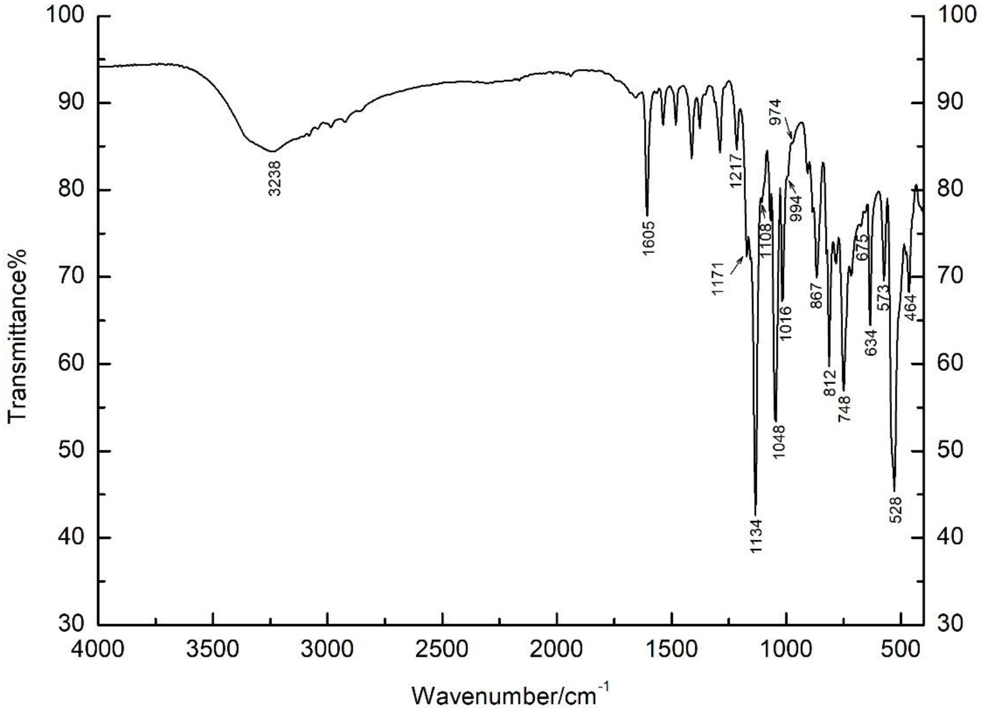

Figure S12. FTIR spectrum of coordination polymer Ni-ICR-20.

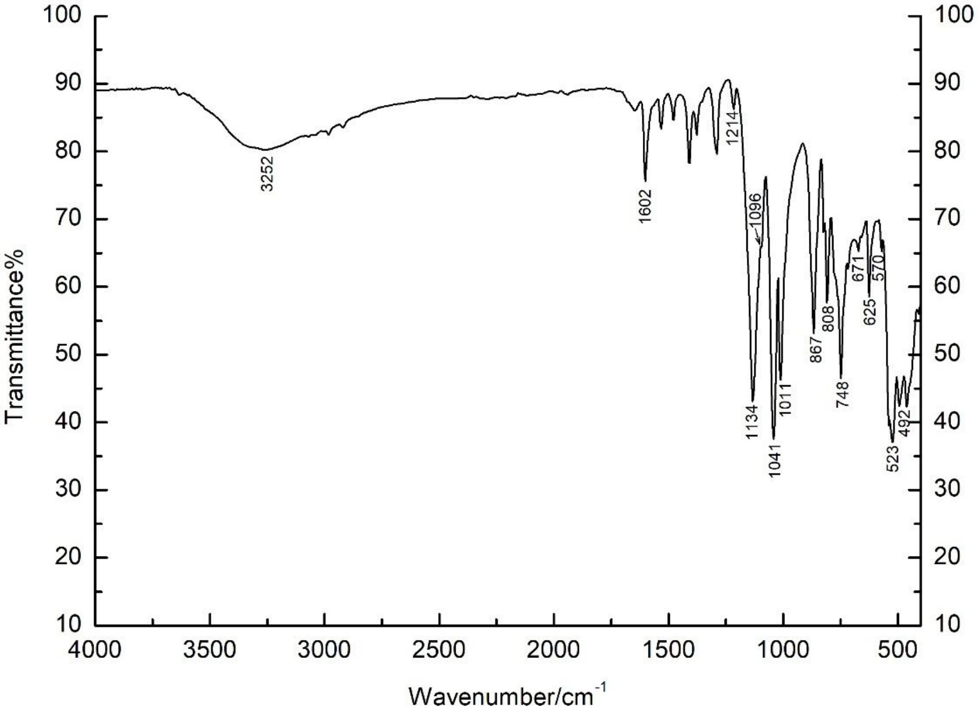

Figure S13. FTIR spectrum of coordination polymer Fe-ICR-21.

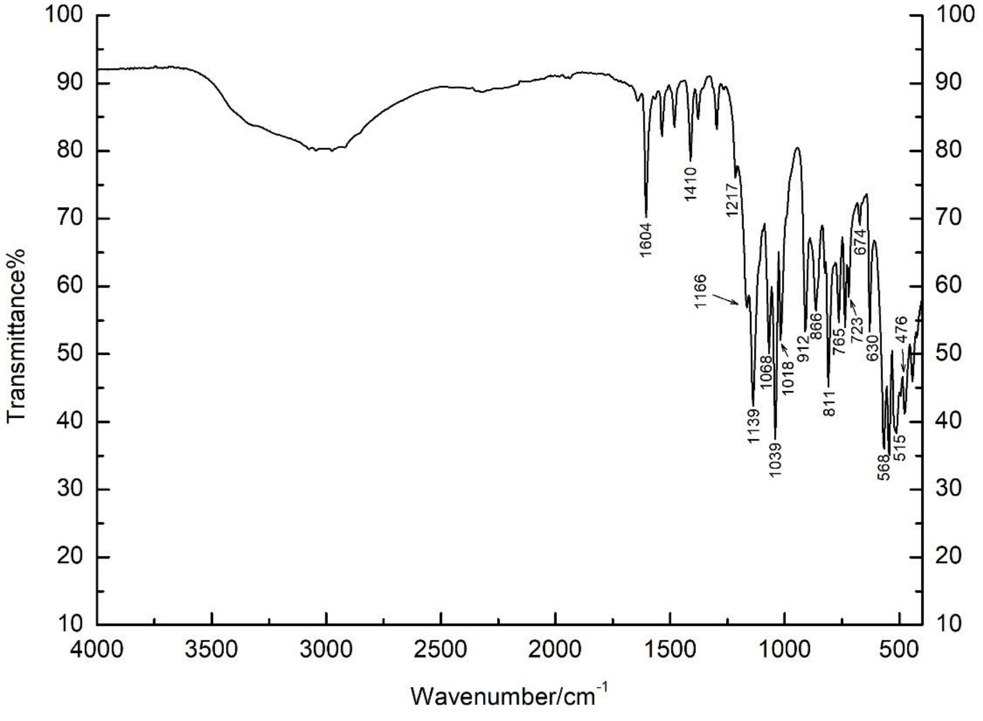

Figure S14. FTIR spectrum of coordination polymer Co-ICR-21.

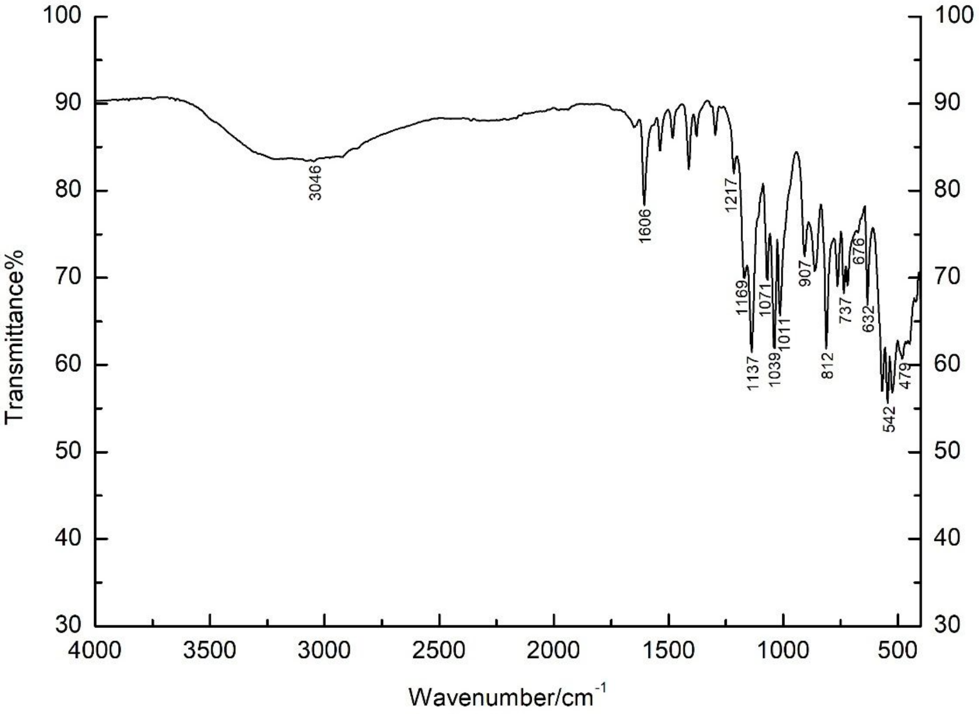

Figure S15. FTIR spectrum of coordination polymer Ni-ICR-21.

3. TG analysis

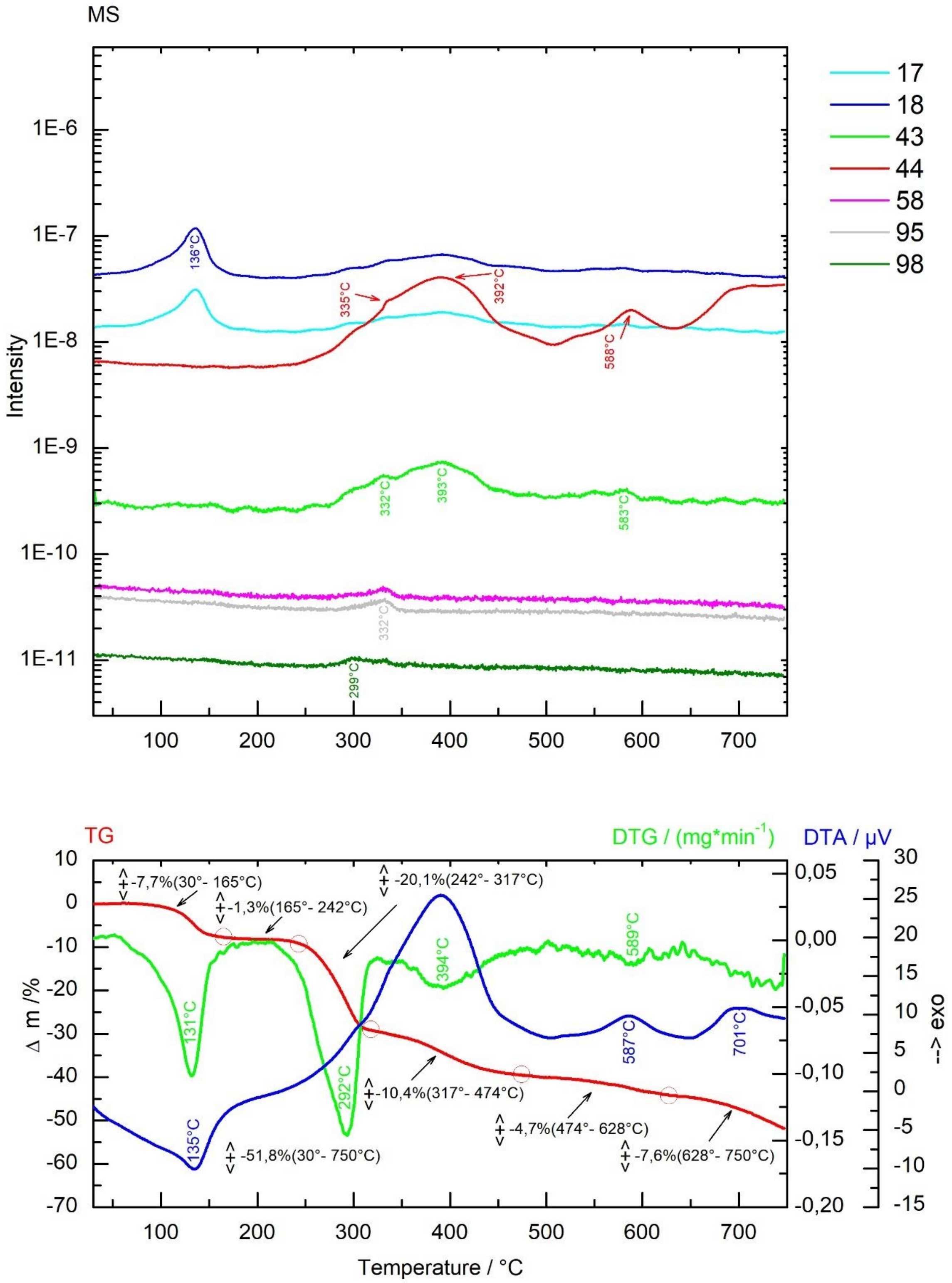

Figure S16. TGA/DTA curves and the evolution of gases for Fe-ICR-20 on air; m/z = 17 - OH, m/z = 18 - H_2O, m/z = 44 - CO_2, m/z = 43, 58 and 95 - acetone, and m/z = 98 - H_3PO_4.

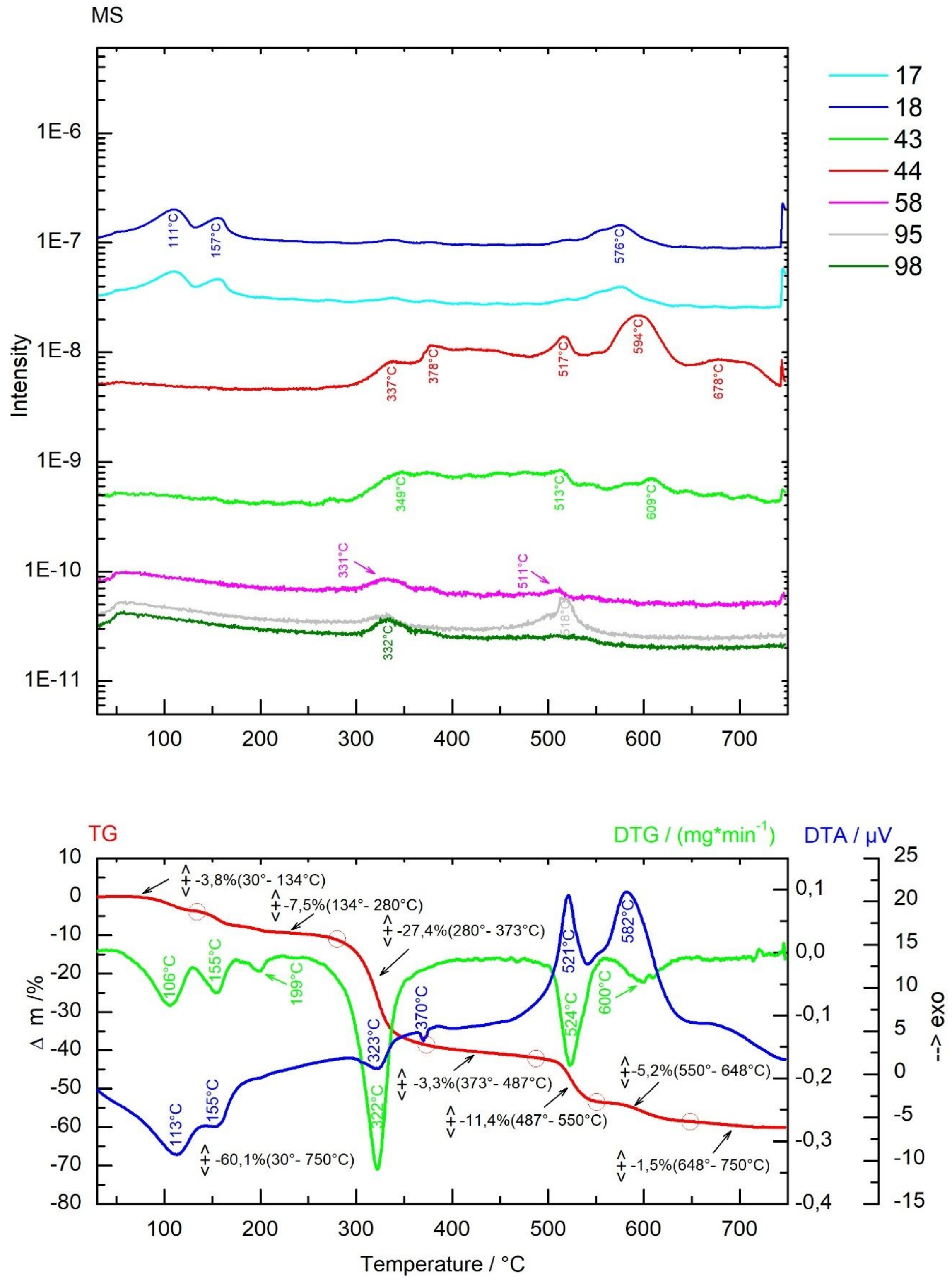

Figure S17. TGA/DTA curves and the evolution of gases for Co-ICR-20 on air; m/z = 17 - OH, m/z = 18 - H_2O, m/z = 44 - CO_2, m/z = 43, 58 and 95 - acetone, and m/z = 98 - H_3PO_4.

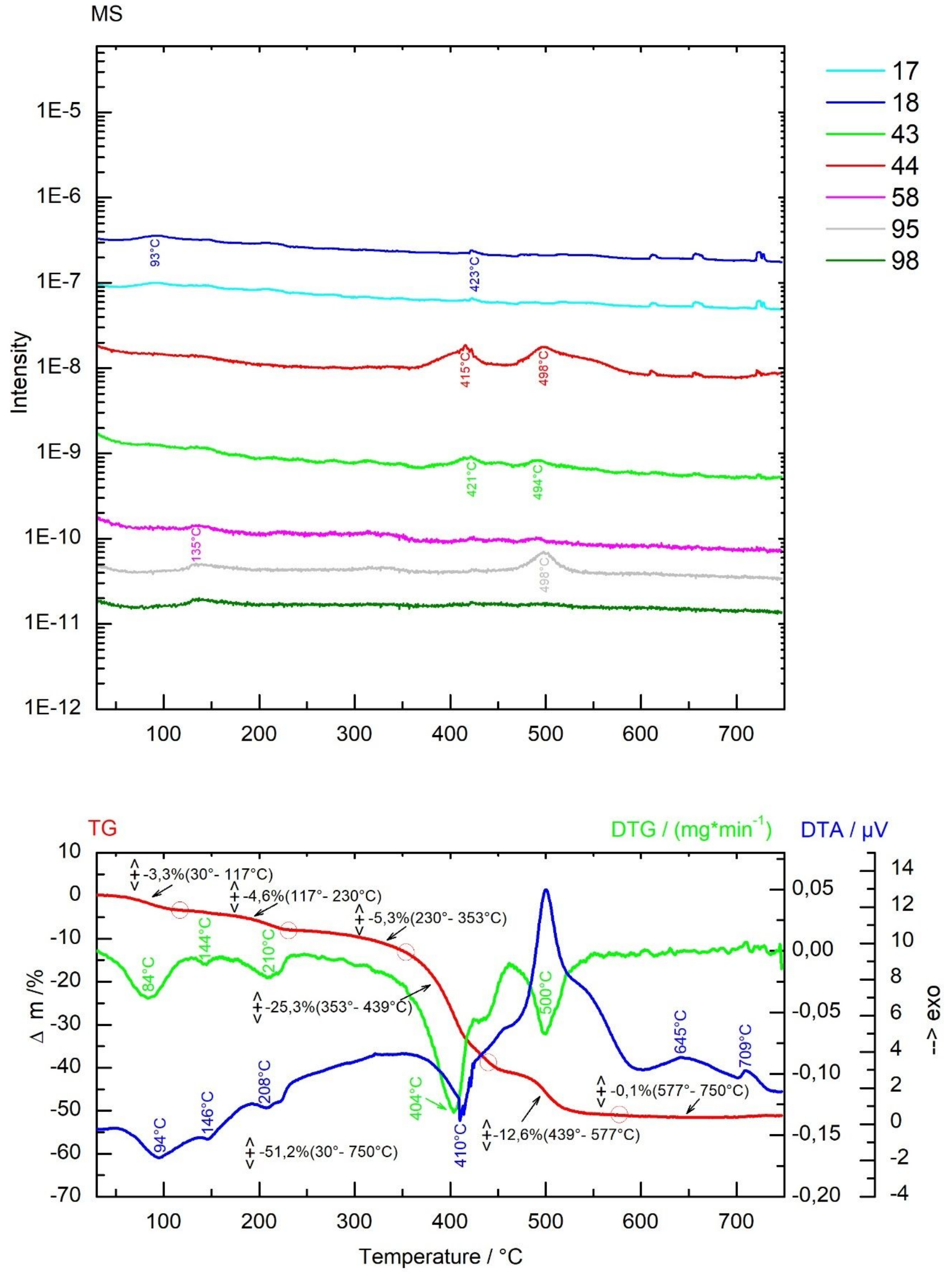

Figure S18. TGA/DTA curves and the evolution of gases for Ni-ICR-20 on air; m/z = 17 - OH, m/z = 18 - H_2O, m/z = 44 - CO_2, m/z = 43, 58 and 95 - acetone, and m/z = 98 - H_3PO_4.

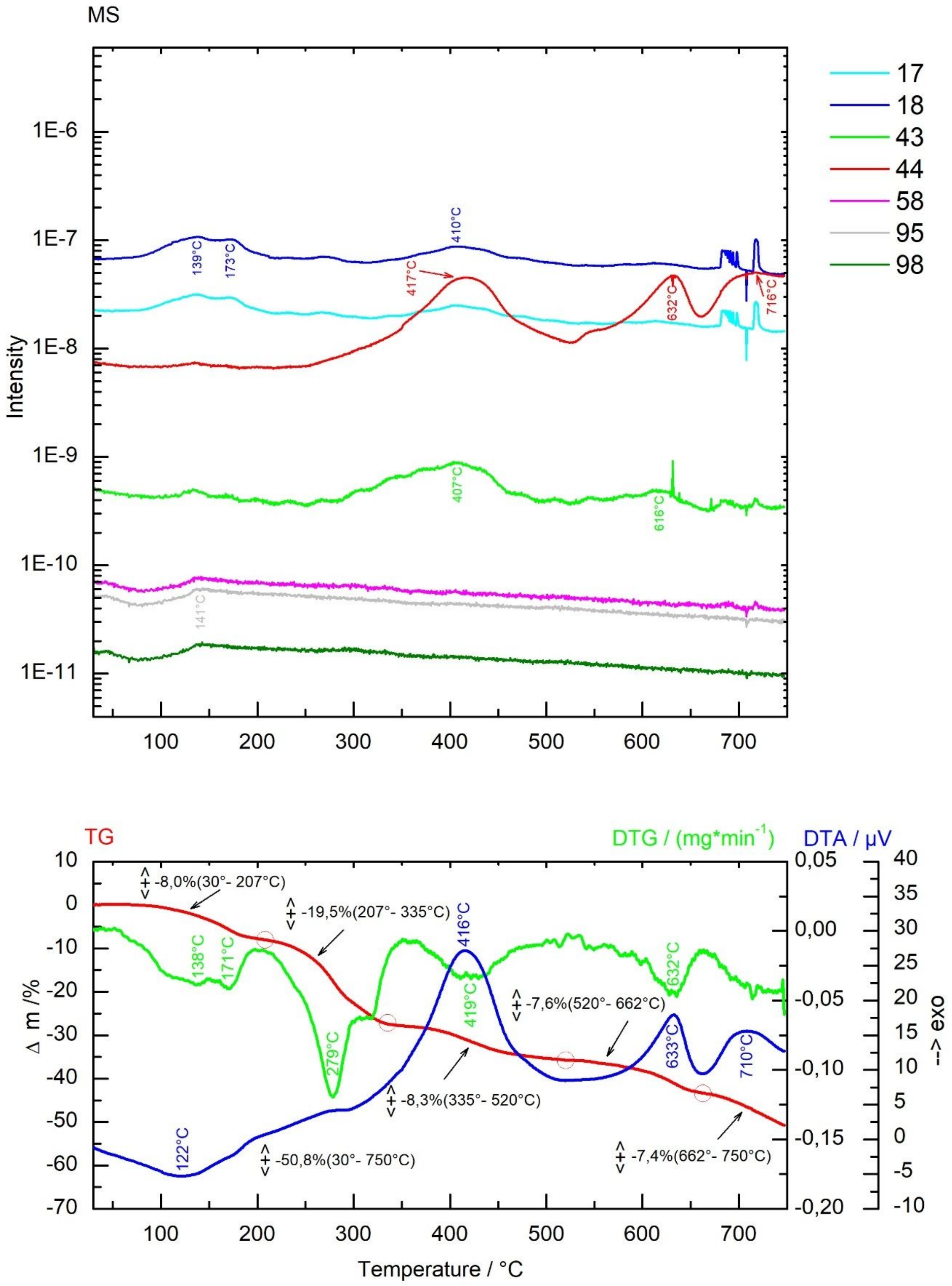

Figure S19. TGA/DTA curves and the evolution of gases for Fe-ICR-21 on air; m/z = 17 - OH, m/z = 18 - H_2O, m/z = 44 - CO_2, m/z = 43, 58 and 95 - acetone, and m/z = 98 - H_3PO_4.

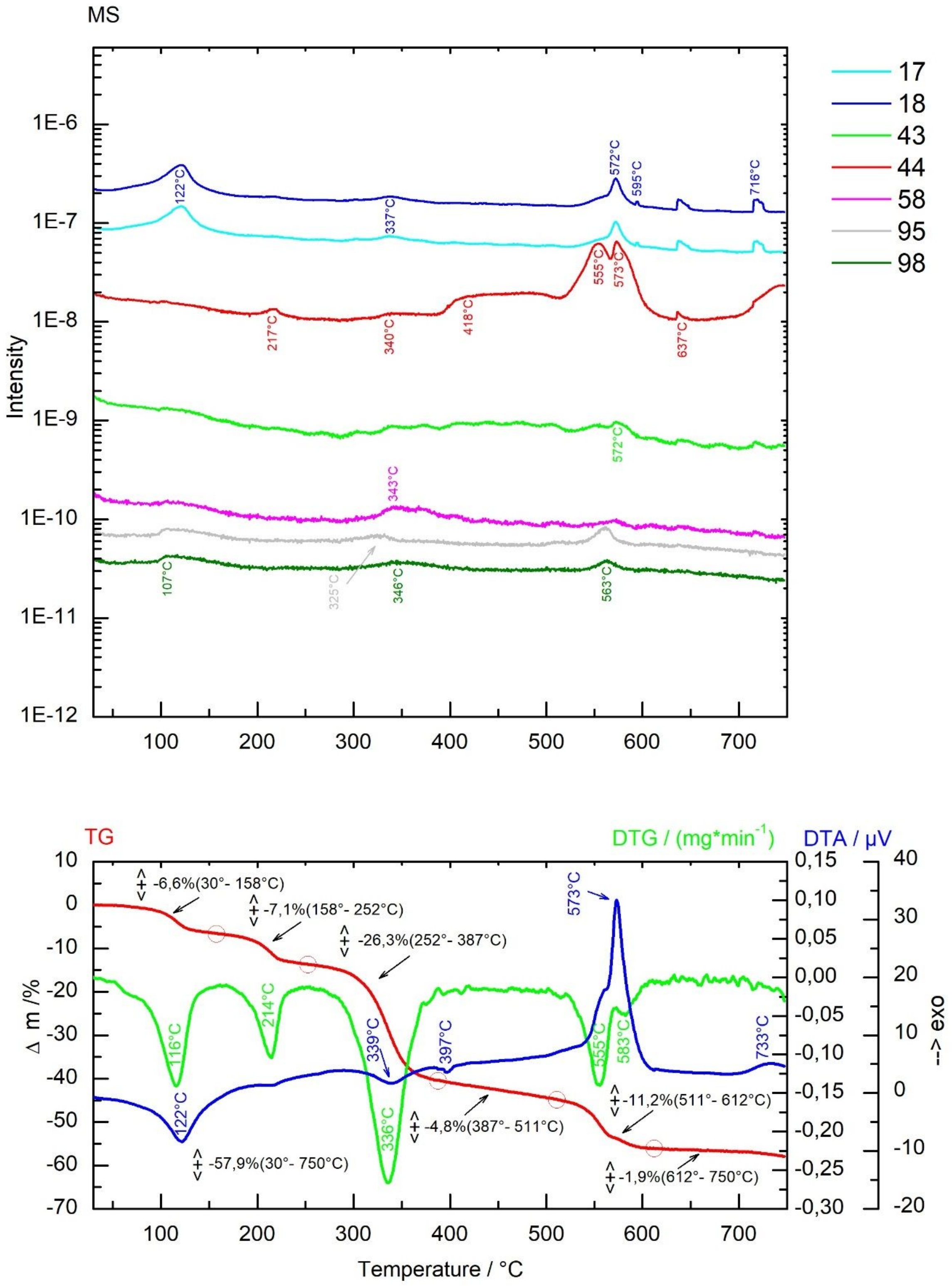

Figure S20. TGA/DTA curves and the evolution of gases for Co-ICR-21 on air; m/z = 17 - OH, m/z = 18 - H_2O, m/z = 44 - CO_2, m/z = 43, 58 and 95 - acetone, and m/z = 98 - H_3PO_4.

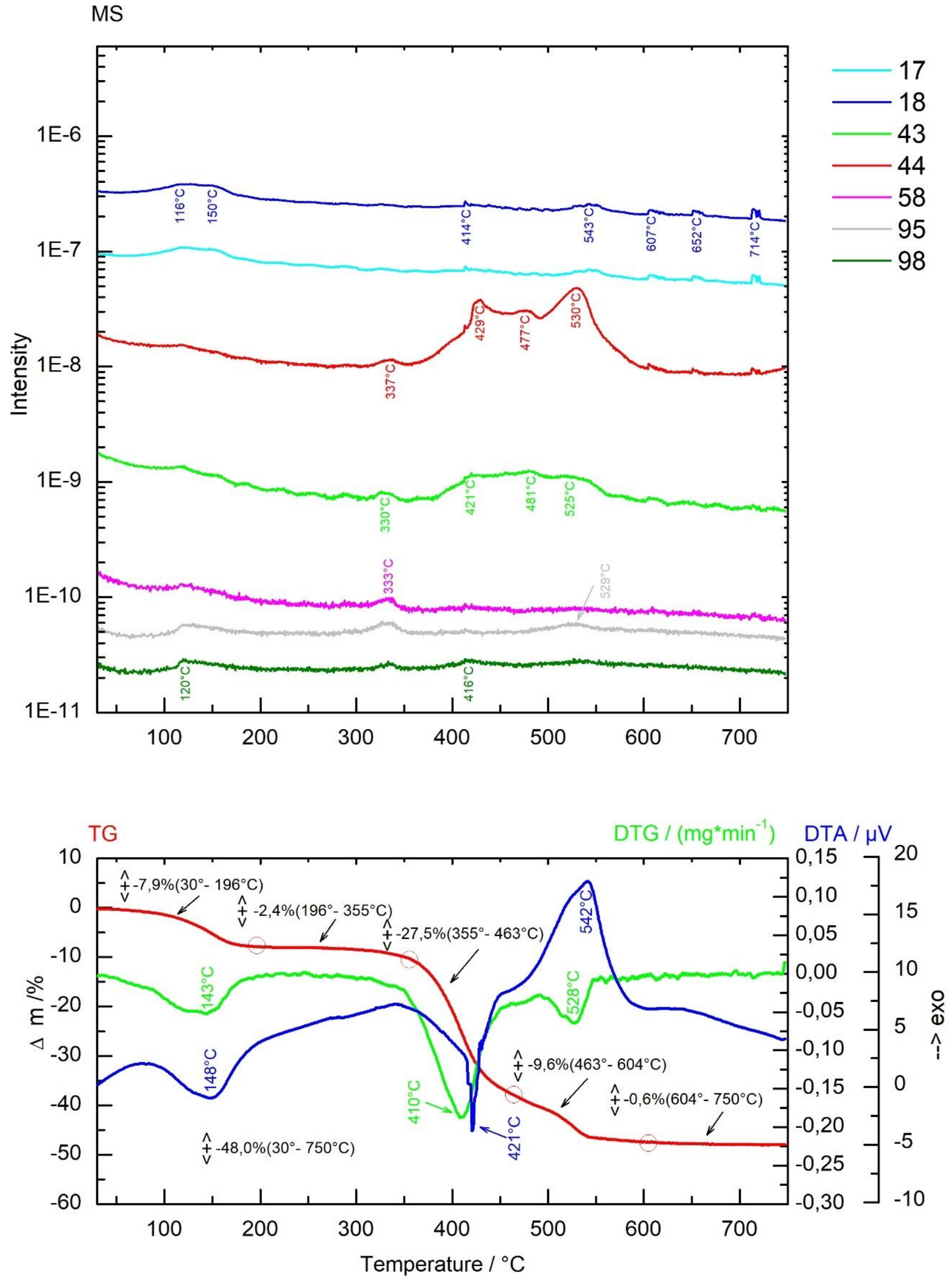

Figure S21. TGA/DTA curves and the evolution of gases for Ni-ICR-21 on air; m/z = 17 - OH, m/z = 18 - H_2O, m/z = 44 - CO_2, m/z = 43, 58 and 95 - acetone, and m/z = 98 - H_3PO_4.

4. CHN analysis

Table S1: CHN elemental analysis of ICR-20 – ICR-21.

	C (calcul.)	C (meas.)	N (calcul.)	N (meas.)	H (calcul.)	H (meas.)
Fe-ICR-20 ($C_{18}H_{22}FeN_2O_6P_2$)	45.02	44.93	5.83	6.05	4.62	4.57
Fe-ICR-21 ($C_{17}H_{20}FeN_2O_7P_2$)	42.35	42.81	5.81	5.91	4.18	4.03
Co-ICR-20 ($C_{18}H_{22}CoN_2O_6P_2$)	44.74	44.43	5.80	5.75	4.59	4.54
Co-ICR-21 ($C_{17}H_{20}CoN_2O_7P_2$)	42.08	42.02	5.77	5.8	4.15	4.27
Ni-ICR-20 ($C_{18}H_{22}NiN_2O_6P_2$)	44.76	45.62	5.80	6.17	4.59	4.67
Ni-ICR-21 ($C_{17}H_{20}NiN_2O_7P_2$)	42.10	42.17	5.78	5.68	4.16	4.15

5. Crystallographic parameters

Table S2: Crystallographic data for ICR-20 and ICR-21.

Compound	**Fe-ICR-20**	**Co-ICR-20**	**Ni-ICR-20**	**Fe-ICR-21**	**Co-ICR-21**
CCDC No.	2408442	2408443	2408445	2408446	2408450
Empirical formula	$C_{18}H_{22}FeP_2N_2O_6$	$C_{18}H_{22}CoP_2N_2O_6$	$C_{18}H_{22}NiP_2N_2O_6$	$C_{17}H_{20}FeP_2N_2O_7$	$C_{17}H_{20}CoP_2N_2O_7$
Formula weight	480.16	483.24	483.04	482.14	485.22
Crystal system	monoclinic				
Space group	$P2_1/n$				
a/Å	11.0668(5)	11.0771(6)	11.0682(2)	10.9579(1)	11.0000(3)
b/Å	9.6700(5)	9.6169(7)	9.5592(1)	9.4636(1)	9.3583(2)
c/Å	19.1301(9)	19.008(1)	18.8123(2)	19.0326(2)	18.8935(4)
α/°	90	90	90	90	90
β/°	94.133(4)	93.839(4)	93.502(1)	95.508(1)	95.061(2)
γ/°	90	90	90	90	90
Volume/Å^3	2041.9(2)	2020.3(2)	1986.68(5)	1964.59(3)	1937.34(8)
Z	4	4	4	4	4
ρ_{calc} g/cm^3	1.562	1.589	1.615	1.630	1.664
μ/mm^{-1}	7.750	8.506	3.286	8.098	8.914
F(000)	992.0	996.0	1000.0	992.0	996.0
Crystal size/mm	0.176×0.058 ×0.026	0.085×0.031 ×0.021	0.301×0.066 ×0.049	0.147×0.05 ×0.016	0.176×0.058 ×0.026
2Θ range for data collection/°	9.27 to 155.828	9.326 to 136.464	9.036 to 149.526	8.958 to 136.48	8.974 to 160.666
Reflections collected	10895	17191	30147	20819	19527
Independent reflections	3874	3621	4060	3603	4121
Data/restraints/ parameters	3874/0/268	3621/0/268	4060/8/278	3603/4/278	4121/7/276
Goodness-of-fit on F^2	0.989	0.973	1.049	1.031	1.080
R [I>=2σ(I)]	R_1 = 0.0670, wR_2 = 0.1868	R_1 = 0.0682, wR_2 = 0.1557	R_1 = 0.0390, wR_2 = 0.1020	R_1 = 0.0417, wR_2 = 0.1094	R_1 = 0.0567, wR_2 = 0.1569
R_w (all data)	R_1 = 0.1012, wR_2 = 0.2161	R_1 = 0.1270, wR_2 = 0.1848	R_1 = 0.0400, wR_2 = 0.1025	R_1 = 0.0438, wR_2 = 0.1111	R_1 = 0.0631, wR_2 = 0.1616

Largest diff. peak/hole e Å^{-3}	0.81/-0.81	0.53/-0.58	0.68/-0.55	1.74/-0.44	1.14/-0.76

Table S3: Bond lengths in the metal coordination sphere of ICR-20 and ICR-21.

	Fe-ICR-20	Co-ICR-20	Ni-ICR-20	Fe-ICR-21	Co-ICR-21
M-O1	2.150(4)	2.090(5)	2.079(2)	2.114(2)	2.067(2)
M-O2	2.067(4)	2.074(4)	2.052(2)	2.084(2)	2.093(3)
M-O3	2.069(4)	2.098(4)	2.082(2)	2.043(2)	2.060(3)
M-O1W	2.183(4)	2.123(5)	2.081(2)	2.159(2)	2.101(3)
M-N1	2.197(5)	2.162(5)	2.094(2)	2.217(2)	2.176(3)
M-N2	2.199(5)	2.152(5)	2.090(2)	2.203(2)	2.161(3)

Table S4: Bond angles in the metal coordination sphere of ICR-20 and ICR-21.

	Fe-ICR-20	Co-ICR-20	Ni-ICR-20	Fe-ICR-21	Co-ICR-21
N1-M-N2	174.5(2)	175.4(2)	175.87(9)	174.21(8)	175.0(1)
O1-M-O1W	173.1(2)	173.9(2)	174.36(8)	175.05(7)	175.5(1)
O2-M-O3	178.5(2)	178.2(2)	178.69(8)	176.76(7)	176.76(9)
N1-M-O1	87.5(2)	87.6(2)	87.55(8)	88.99(8)	88.8(1)
N1-M-O2	87.1(2)	87.1(2)	87.39(8)	86.84(8)	86.8(1)
N1-M-O3	92.6(2)	92.0(2)	91.33(8)	92.69(8)	92.2(1)
N1-M-O1W	86.3(2)	86.9(2)	87.26(9)	86.07(8)	86.7(1)
N2-M-O1	91.7(2)	92.4(2)	91.63(8)	92.32(8)	92.6(1)
N2-M-O2	87.5(2)	88.3(2)	88.53(8)	87.53(8)	88.4(1)
N2-M-O3	92.9(2)	92.6(2)	92.76(8)	92.85(8)	92.5(1)
N2-M-O1W	94.3(2)	93.0(2)	93.40(9)	92.63(8)	91.9(1)
O1-M-O2	86.3(2)	87.7(2)	86.98(8)	89.25(7)	89.43(9)
O1-M-O3	95.2(2)	93.9(2)	93.18(8)	93.95(7)	93.6(1)
O2-M-O1W	90.4(2)	89.6(2)	90.59(8)	90.84(7)	90.33(9)
O3-M-O1W	88.1(2)	88.8(2)	89.14(8)	85.92(7)	86.5(1)

6. ICR-22

The coordination polymer ICR-22 crystallizes in the monoclinic $P2_1/n$ space group. The structure (Figure S22) contains two independent metal centres, marked M1 and M2. In this case, the usual M-O-P-O-M infinite chains are not formed and instead two M1 centres and one M2 centre are connected by phosphonate groups into trimers while the phosphinate groups remain uncoordinated. The structure is further interlinked by the bipyridine molecules into a 3D network.

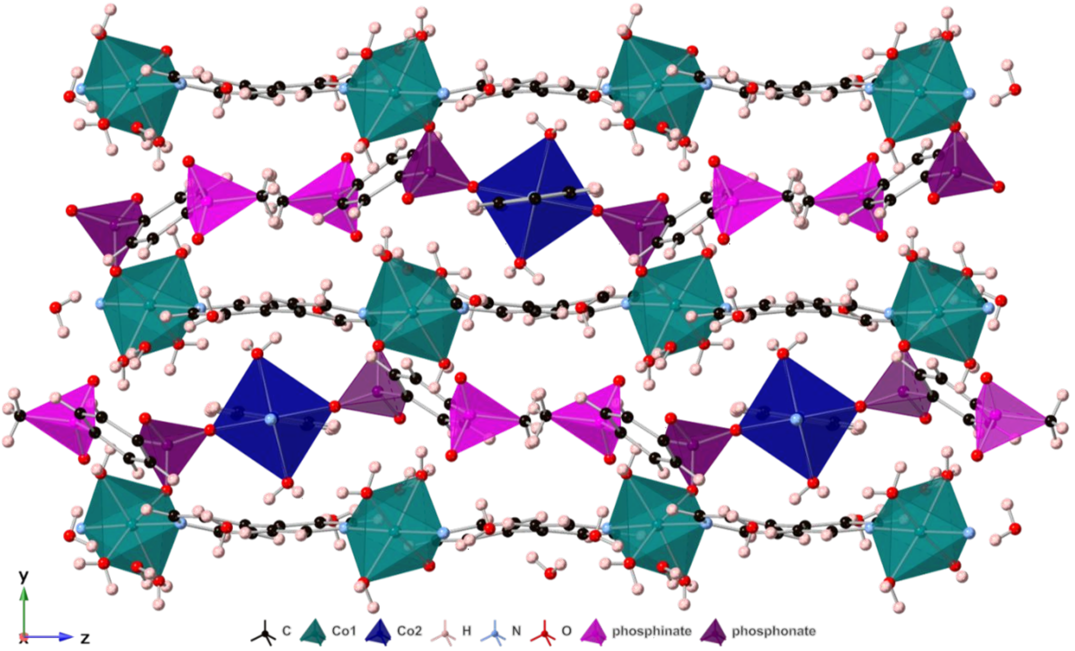

Figure S22. The structure of ICR-22

Table S5. Crystallographic data for ICR-22.

Compound	**Fe-ICR-22**	**Co-ICR-22**
CCDC No.	2448058	2448042
Empirical formula	$C_{44}H_{66}Fe_3N_6O_{24}P_4$	$C_{44}H_{66}Co_3N_6O_{24}P_4$
Formula weight	1354.45	1363.69
Crystal system	monoclinic	
Space group	$P2_1/n$	
a/Å	11.6373(1)	11.5550(1)
b/Å	14.2969(1)	14.2796(2)
c/Å	17.5096(2)	17.4241(2)
α/°	90	90
β/°	101.472(1)	101.102(1)
γ/°	90	90
Volume/Å^3	94.133(4)	93.839(4)
Z	90	90
ρ_{calc} g/cm^3	1.576	1.605
μ/mm^{-1}	7.836	8.653
F(000)	1404.0	1410.0
Crystal size/mm	0.556 × 0.205 × 0.053	0.345 × 0.076 × 0.07
2Θ range for data collection/°	8.05 to 133.148	8.066 to 160.78
Reflections collected	35748	29529
Independent reflections	5031	6071
Data/restraints/parameters	5031/22/416	6071/22/407
Goodness-of-fit on F^2	1.071	1.092
R [I>=2σ(I)]	R_1 = 0.0445, wR_2 = 0.1222	R_1 = 0.0390, wR_2 = 0.1083
R_w (all data)	R_1 = 0.0452, wR_2 = 0.1229	R_1 = 0.0403, wR_2 = 0.1091

Largest diff. peak/hole e Å^{-3}	1.14/-0.87	0.82/-0.69

7. Magnetic properties

The susceptibility and magnetization data of ICR-20 and ICR-21 were rather similar. Experimental data of the respective samples at selected temperatures and fields are compared in Fig. S22, S23 and S24. Magnetization data are accompanied with simulated Brillouin curves at the same temperatures, using the average g_{iso} value from the fits in PHI.

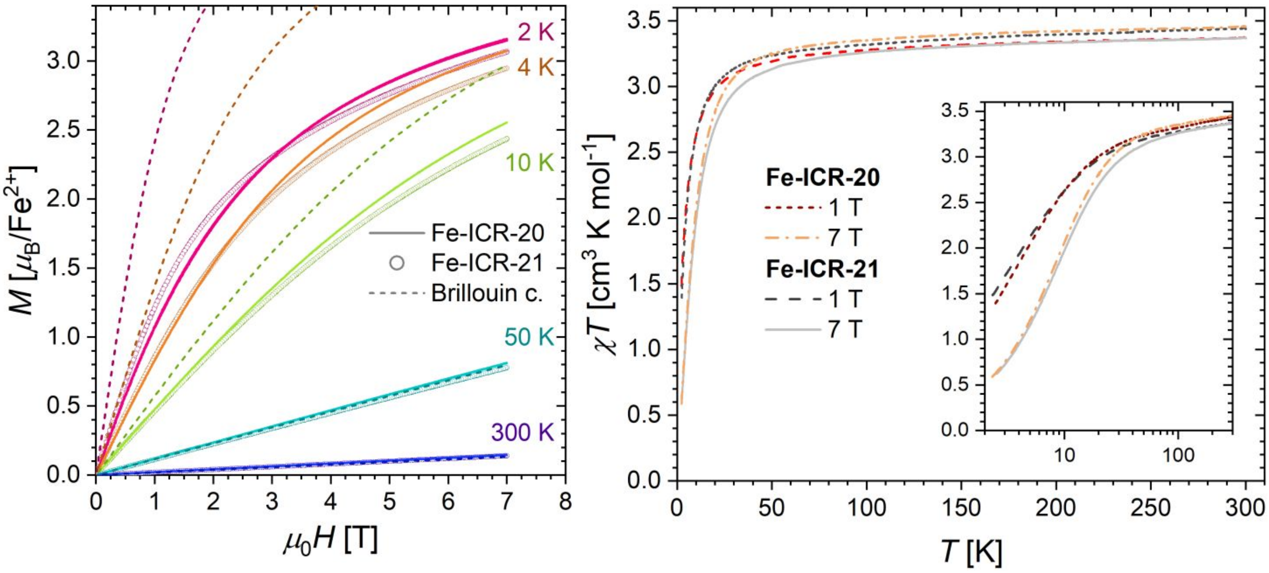

Figure S23. Magnetization curves with simulated Brillouin curves (for $g = 2.075$) at selected temperatures (left) and χT at selected applied fields of Fe-ICR-20 and Fe-ICR-21.

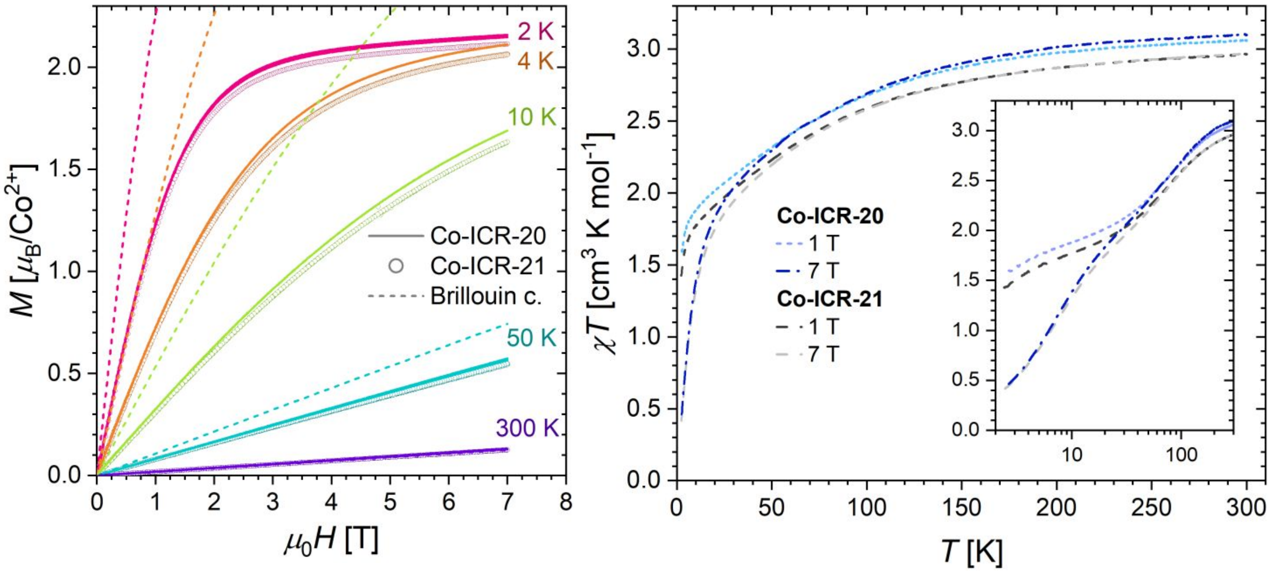

Figure S24. Magnetization curves with simulated Brillouin curves (for $g = 2.535$) at selected temperatures (left) and χT at selected applied fields of Co-ICR-20 and Co-ICR-21.

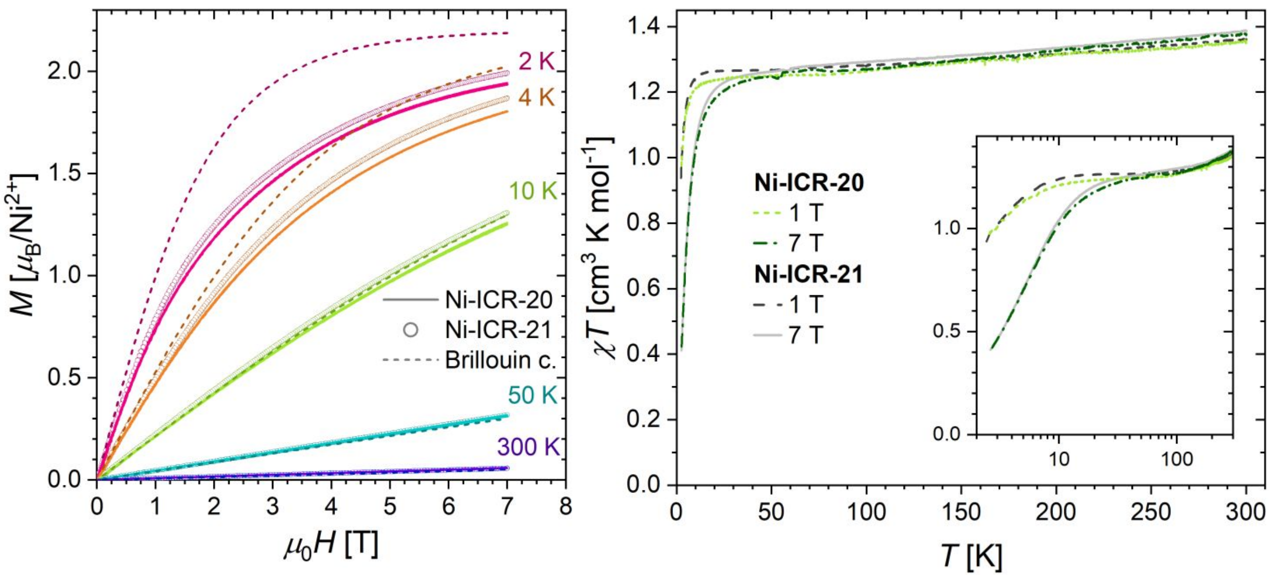

Figure S25. Magnetization curves with simulated Brillouin curves (for $g = 2.20$) at selected temperatures (left) and χT at selected applied fields of Ni-ICR-20 and Ni-ICR-21.

7.1. Curie-Weiss fits

As a first estimate of the strength of the magnetic interaction between metal ions, we fitted the reciprocal DC susceptibility in the temperature range 100–295 K by the Curie-Weiss law

$$\chi^{-1}(T) = \left(\frac{C}{T-\theta_{\mathrm{CW}}} + \chi_{\mathrm{TI}}\right)^{-1}, \qquad (1)$$

where C is the Curie constant, θ_{CW} the Curie-Weiss temperature and χ_{TI} the temperature-independent susceptibility. The effective moment per metal ion, expressed as the number of Bohr magnetons μ_{B}, can be calculated from $\mu_{\mathrm{eff}} = \sqrt{\frac{3k_{\mathrm{B}}C}{N_{\mathrm{A}}\mu_{\mathrm{B}}^2}}$, with k_{B} and N_{A} representing the Boltzmann constant and Avogadro number. Alternatively, the effective g-factor, which points out the departure from the spin-only behavior, can be expressed as $g_{\mathrm{eff}} = \frac{\mu_{\mathrm{eff}}}{\sqrt{S(S+1)}}$, considering the spin S of the ions. Using the mean-field approximation, one can estimate the effective exchange constant between the nearest neighbours as $J_{\mathrm{eff}} = \frac{3k_{\mathrm{B}}\theta_{\mathrm{CW}}}{[zS(S+1)]}$, where $z = 2$ is the

number of nearest neighbors. Even after the diamagnetic correction (see the Methods section in the main text), a residual diamagnetic contribution ranged approx. between $-1 \cdot 10^{-3}$ and $-5 \cdot 10^{-5}$ cm^3 mol^{-1}. The results of the fit, averaged over obtained values in the various applied fields, are summarized in Table S5, together with distances of nearest and next-nearest metal atoms and the angle M–P–M between metal atoms connected by the phosphonate/phosphinate units. The reciprocal susceptibilities with high-field examples of the Curie-Weiss fit are shown in Figures S25-S27.

The negative Curie-Weiss temperatures show predominantly antiferromagnetic interaction between the metal centers for Fe and Co coordination polymers, whereas the fit indicates a possibly ferromagnetic interaction for those containing Ni. Nevertheless, the observed θ_{CW} of Ni-ICR-20 falls within the usual range (e.g., θ_{CW} ranging from -2.6 to -0.7 K for interaction between Ni^{2+} ions doubly bridged by O–P–O).[1]

However, the Curie-Weiss fit of the individual curves leads to a rather large spread of the fitted values for different fields, as it is highly sensitive to experimental aspects and does not consider the effect of the single-ion anisotropy. The variation is most critical in the case of θ_{CW} (and thereby J_{eff}) which should reflect the very weak super-superexchange interaction between the nearest neighbors. In addition, the strong anisotropy of Co^{2+} ions leads to an unrealistic overestimation of θ_{CW} even for a fit at higher temperatures 150–295 K. For this reason, the fit using spin Hamiltonian was attempted in the follow-up step, as described in the main text and in Section 7.3 in more detail.

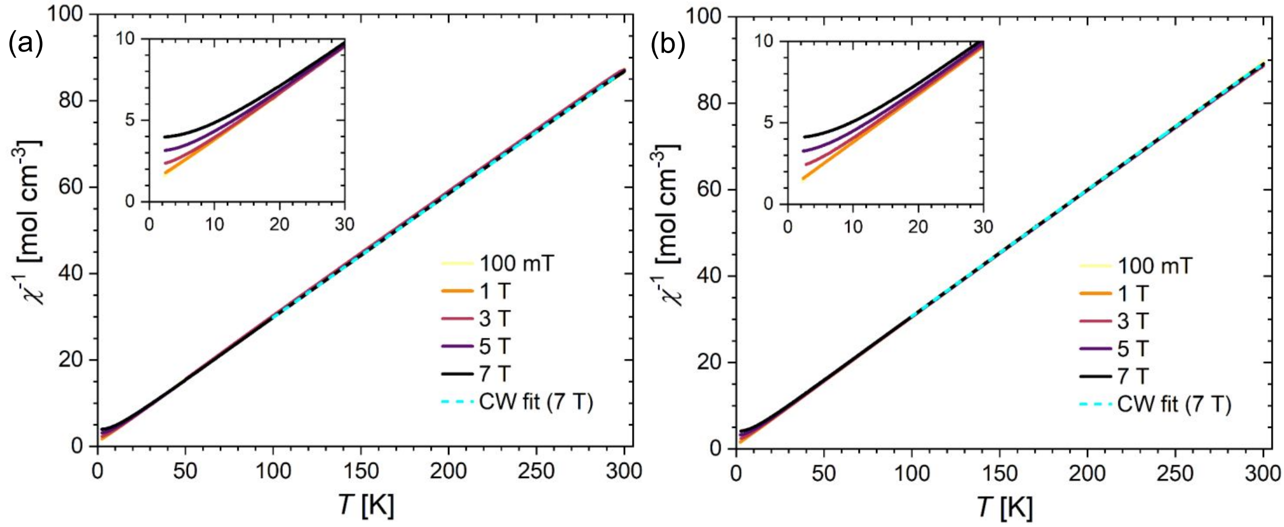

Figure S26. Reciprocal susceptibility of (a) Fe-ICR-20 and (b) Fe-ICR-21 at various applied fields, with a representative Curie-Weiss fit (100–295 K, 7 T, dashed line); detail at low temperatures is shown in the inset.

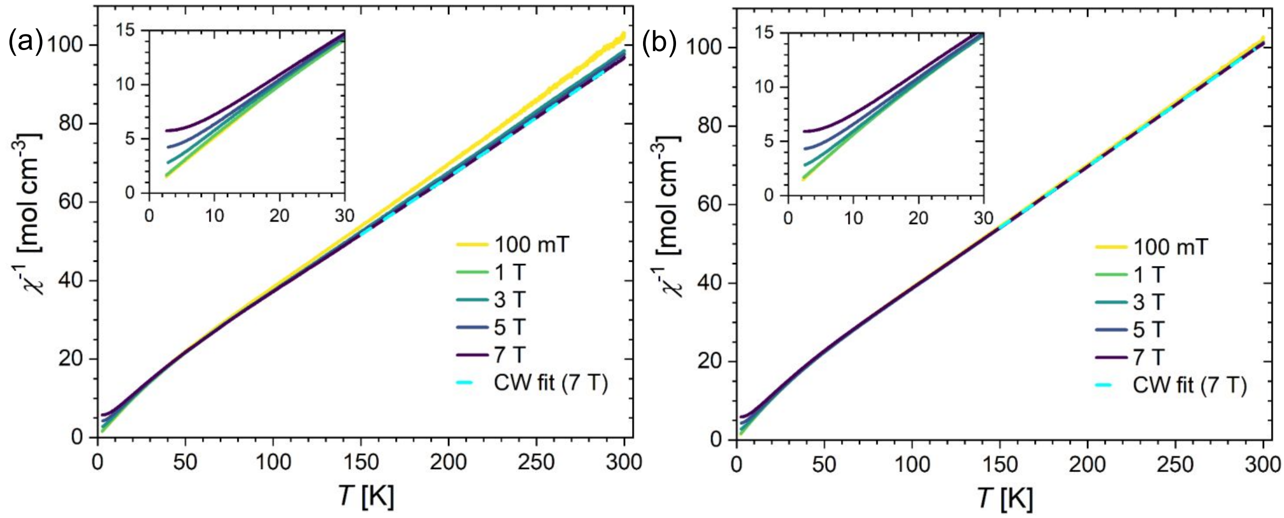

Figure S27. Reciprocal susceptibility of (a) Co-ICR-20 and (b) Co-ICR-21 at various applied fields, with a representative Curie-Weiss fit (150–295 K, 7 T, dashed line); detail at low temperatures is shown in the inset.

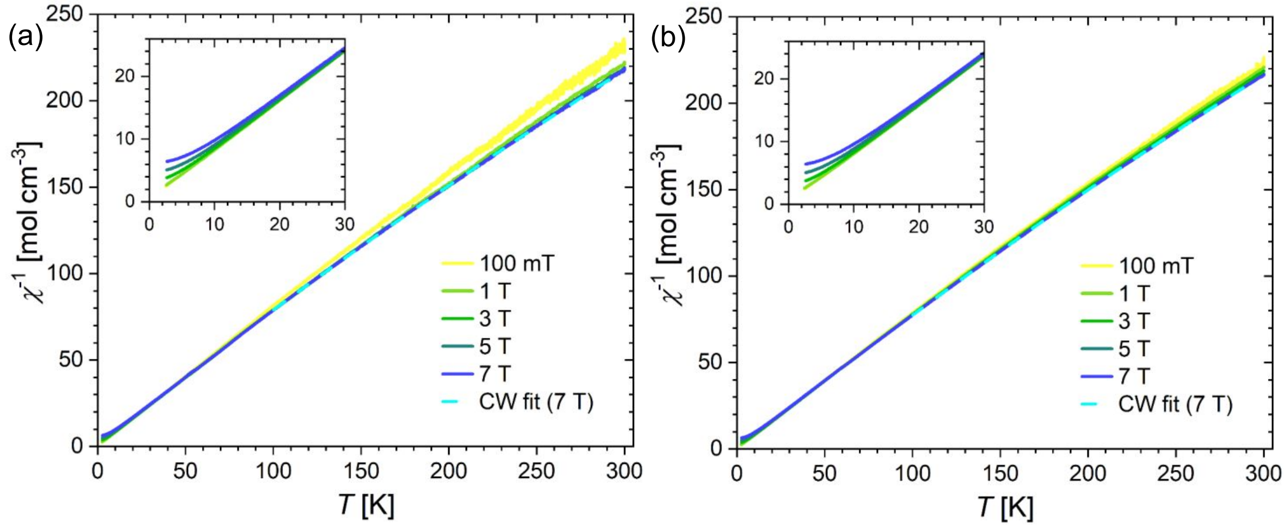

Figure S28. Reciprocal susceptibility of (a) Ni-ICR-20 and (b) Ni-ICR-21 at various applied fields, with a representative Curie-Weiss fit (100–295 K, 7 T, dashed line); detail at low temperatures is shown in the inset.

Table S6: Magnetic parameters of ICR-20 and ICR-21 based on the Curie-Weiss fit in the temperature range 100–295 K (*150–295 K), where $\mu_{s\text{-}o}$ denotes the theoretical spin-only moment per metal atom. The table summarizes average values over fits in applied fields 1–7 T, with the sample standard deviation in the brackets. In addition, the distance between the nearest, $(M\text{-}M)_{nn}$, and second-nearest, $(M\text{-}M)_{nnn}$, metal atoms in the chain and the angle between metal atoms connected by the phosphinate/phosphonate units are shown based on the structures at 100 K.

	$(M\text{-}M)_{nn}$ [Å]	$(M\text{-}M)_{nnn}$ [Å]	∠M-P-M [deg]	$\mu_{s\text{-}o}$ [μ_B]	μ_{eff} [μ_B]	θ_{CW} [K]	J_{eff} [K]	C [cm^3 K mol^{-1}]	g_{eff}
Fe-ICR-20	5.592(1)	9.6700(5)	111.15(5)	*4.90*	5.23(2)	-3.5(8)	-0.9(2)	3.42(3)	2.14(1)
Co-ICR-20*	5.544(1)	9.6169(7)	110.48(5)	*3.87*	5.4(1)	-31(4)	-12(2)	3.6(1)	2.76(6)
Ni-ICR-20	5.4955(5)	9.5592(1)	110.34(3)	*2.83*	3.16(2)	-2(1)	-1.8(8)	1.25(2)	2.24(1)
Fe-ICR-21	5.4549(4)	9.4636(1)	106.07(2)	*4.90*	5.18(1)	-3.3(5)	-0.8(1)	3.36(2)	2.12(1)
Co-ICR-21*	5.4019(6)	9.3583(3)	105.58(3)	*3.87*	5.11(3)	-25(2)	-9.9(6)	3.26(4)	2.64(2)
Ni-ICR-21	-	-	-	*2.83*	3.10(1)	2.6(4)	1.9(3)	1.20(1)	2.19(1)

7.2. From 1D chain to single-ion approximation

In both ICR-20 and ICR-21 structures, the chains form rather isolated 1-dimensional magnetic units, in which each metal ion interacts with its two neighbors by super-superexchange interaction through phosphonate or phosphinate groups. Nevertheless, the temperature-

dependent DC susceptibility data do not show any clear maximum typical for 1D systems with antiferromagnetic intrachain interaction.[2] Not only are the 1D intrachain interactions weak, but their influence on low-temperature susceptibility is also obscured by single-ion anisotropy. Consequently, standard models for extracting the coupling constant from the susceptibility of uniform chains, such as those proposed by Fisher *et al.*[3] or Hiller *et al.*[4] are inapplicable. Similarly, the exchange is too weak to study the difference in the magnetic behavior of antiferromagnetic chains related to the presence or absence of the Haldane gap.

The very weak interaction between the magnetic ions in the chain allows us to use a single-ion approximation and to include the interaction with other magnetic ions through the mean-field approximation. Both magnetization and susceptibility datasets were fitted simultaneously for each sample in PHI.[5] Though reminiscent of an axially distorted octahedral geometry, the highly asymmetrical coordination environment leads to a full lifting of the degeneracy of the energy levels. Therefore, spin-only approximation was employed, where higher states admixing to the ground state result in an anisotropic g-factor deviating from 2. Only the second-order crystal-field parameters were considered in the fit. To obtain a rough estimate of the magnitude of the exchange interaction, we also employed a model with two interacting magnetic centers (adding $\hat{H}_{\mathrm{ex}}$ in Eq. 2; three centres provided similar results). The following Hamiltonian was employed, combining the effects of the crystal field and Zeeman interaction (alternatively also exchange interaction), respectively:

$$\hat{H} = \hat{H}_{\mathrm{cf}} + \hat{H}_{\mathrm{Z}} \left(+ \hat{H}_{\mathrm{ex}} \right), \qquad (2)$$

$$\hat{H}_{\mathrm{cf}} = \sum_{i=1,2} \left(D_i \left[\hat{S}_{i,z}^2 - \frac{1}{3} S(S+1) + \frac{E_i}{D_i} \left(\hat{S}_{i,x}^2 - \hat{S}_{i,y}^2 \right) \right] \right), \qquad (3)$$

$$\hat{H}_Z = \mu_B \sum_{i=1,2} \hat{\boldsymbol{S}}_i \boldsymbol{g}_i \boldsymbol{B}. \quad (4)$$

$$\hat{H}_{ex} = -2J\hat{\boldsymbol{S}}_1\hat{\boldsymbol{S}}_2, \quad (5)$$

Here, D and E ($|D| \geq 3E \geq 0$) are zero-field splitting (ZFS) parameters describing the strength of the axial and rhombic crystal field contributions, respectively; $\boldsymbol{g}$ is the g-tensor (Landé tensor), $\boldsymbol{B}$ the applied magnetic field and J is the isotropic exchange coupling constant.

In addition to the susceptibility of the exchange-coupled pair, χ_{dim}, the resulting susceptibility includes a temperature-independent component, χ_{TI}, and the mean-field interactions with other magnetic neighbors zJ:

$$\chi = \frac{\chi_{dim} + \chi_{TI}}{1 - \left(\frac{zJ}{N_A \mu_B^2}\right)(\chi_{dim} + \chi_{TI})}. \quad (6)$$

In this case, χ_{TI} represents the remaining uncorrected diamagnetism, $\chi_{TI} \approx -(4\text{–}9)\cdot 10^{-4}$ cm^3 mol^{-1}.

The resulting parameters are summarized in Table 2 in the main text. The experimental data measured on powders do not allow the determination of the sign of the rhombic parameter E (although a slightly better fit was obtained with $E < 0$ and these values were used to calculate $|E|/D$). The fits using opposite signs of D than those in Table 2 correspond to permutation of coordinate axes; however, they do not fulfill the condition $|E|/D < 1/3$.[6]

7.4. Isothermal magnetization

All measured isothermal magnetization curves are summarized in Figures S28-S30. No hysteresis was observed down to 2 K.

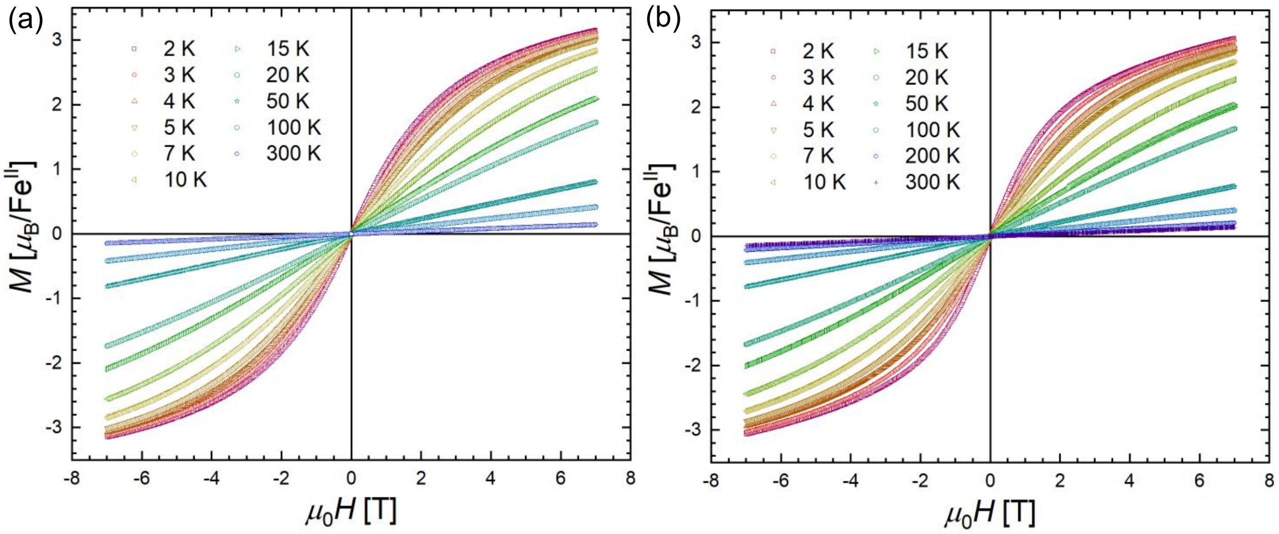

Figure S29. Field-dependent magnetization curves of (a) Fe-ICR-20 and (b) Fe-ICR-21 at various temperatures.

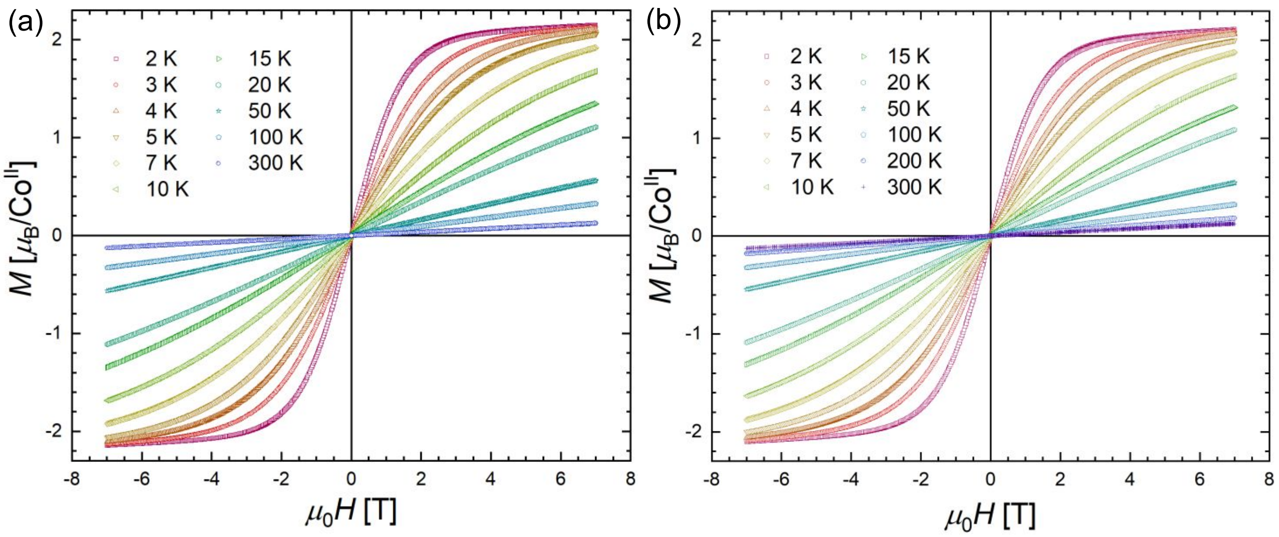

Figure S30. Field-dependent magnetization curves of (a) Co-ICR-20 and (b) Co-ICR-21 at various temperatures.

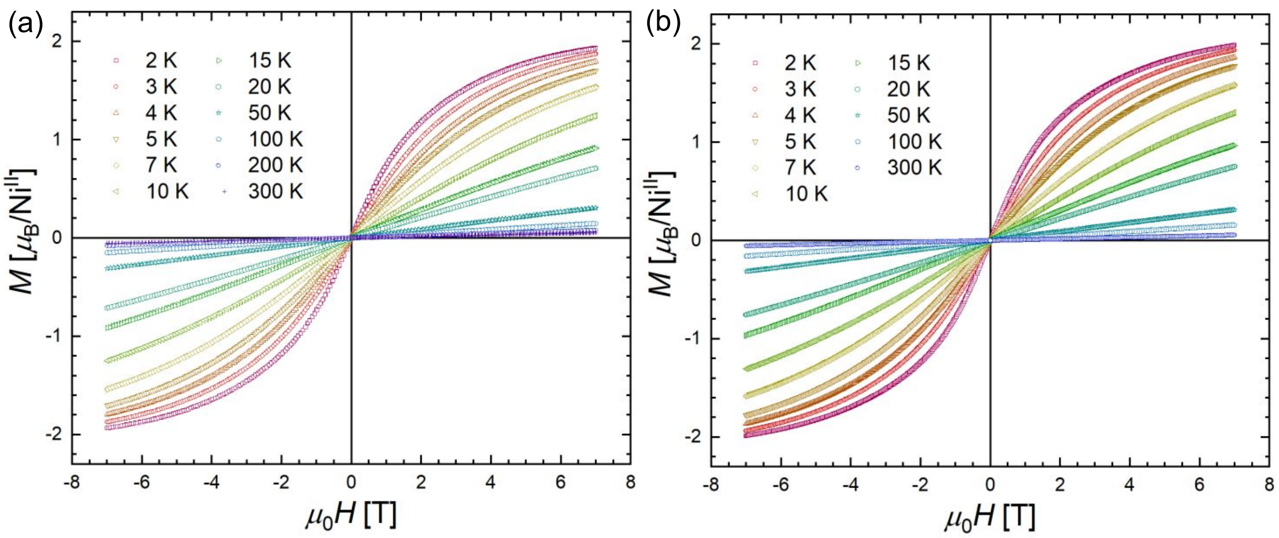

Figure S31. Field-dependent magnetization curves of (a) Ni-ICR-20 and (b) Ni-ICR-21 at various temperatures.

8. Mössbauer spectroscopy

8.1. Stability

The water molecule in the coordination polyhedron of the ICR-20 and ICR-21 compounds (see Figures 1 and 2 in the main text) raises concern about their stability with respect to dehydration. The Mössbauer spectra of Fe-ICR-20 and Fe-ICR-21 were collected in ambient conditions before the temperature-variable measurements, after evacuation at room temperature and again in the air after all measurements (see Figure S31).

In the Mössbauer spectrum of the Fe-ICR-20 sample exposed to vacuum at room temperature, we can observe a substantial increase in the intensity of the D_2 doublet to a value of □31%, due to the sublimation of water molecules from the sample, possibly leaving the coordination sphere of the respective Fe^{2+} ions.[7] After exposure to air, water molecules from the air are re-adsorbed into the voids of the crystal structure and the intensity of the D_2 doublet drops to the usual value of □14%, showing the reversibility of this process. Importantly, the room-temperature spectra

before and after all the measurements are identical, demonstrating the long-term stability of the sample.

The Fe-ICR-21 sample includes an additional doublet D_3 with integral intensity I □ 5%, which probably originates from larger disturbances in the immediate vicinity of the Fe ion, the value of the isomer shift and quadrupole splitting may correspond to Fe^{3+} or Fe^{2+} in the lower spin state $S < 2$. The relative intensity of this doublet slightly increased upon evacuation and did not return to its original content. Nevertheless, the change over the long term was only in the order of units of %.

The evolution of the relative areas of the individual components D_1–D_3 with temperature, based on the spectra acquired in the zero applied magnetic field, is provided in Figures S32 and S33. The relative areas correspond to the relative intensities if we consider identical recoilless fractions (Lamb-Mössbauer factors) of ^{57}Fe nuclei in the corresponding coordination environments.

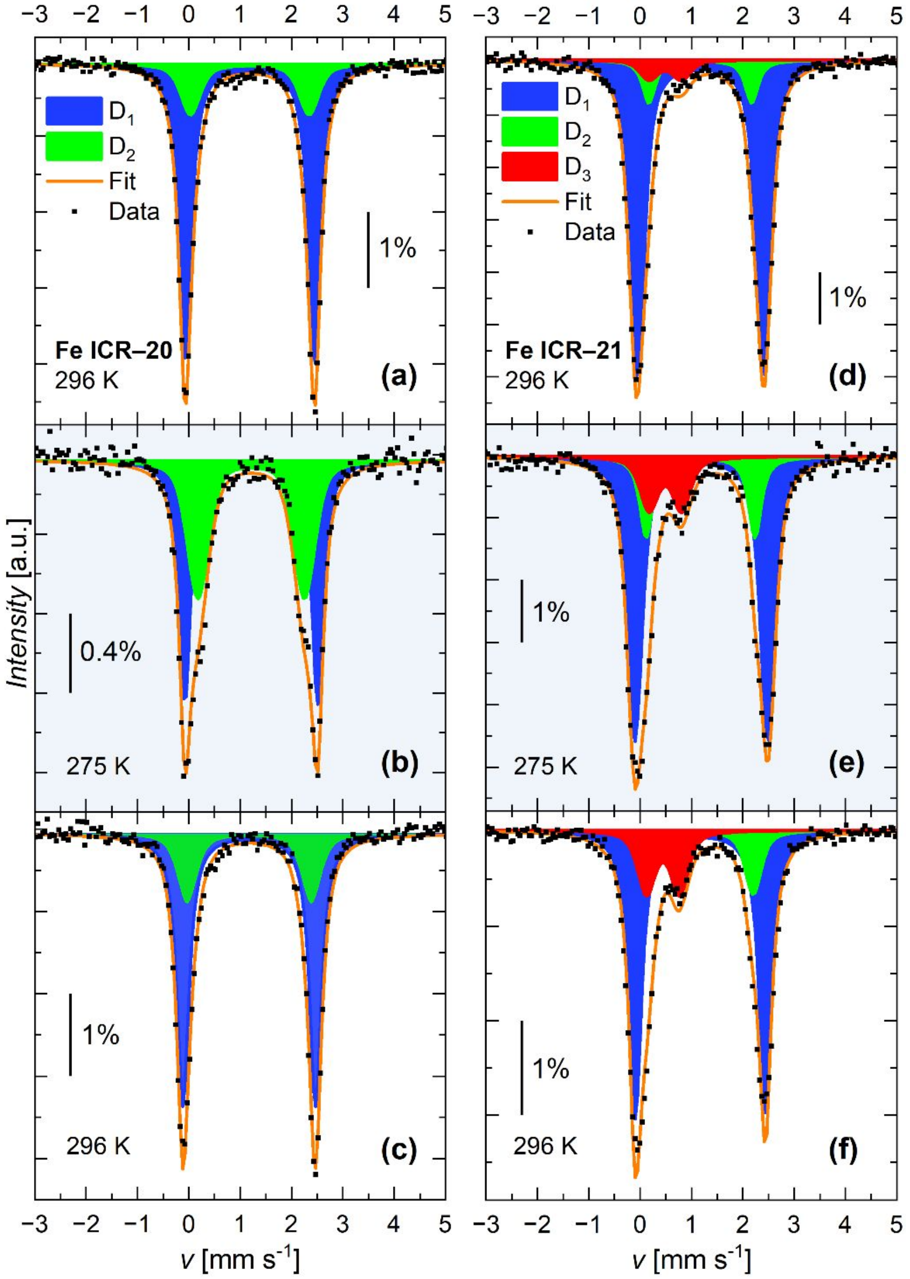

Figure S32. Mössbauer spectra of (a–c) Fe-ICR-20 and (d–f) Fe-ICR-21, measured first at room temperature in air (a,d), at T = 275 K in vacuum after evacuation at room temperature (b,e), and at room temperature again in air, after removing the sample from the vacuum (c,f).

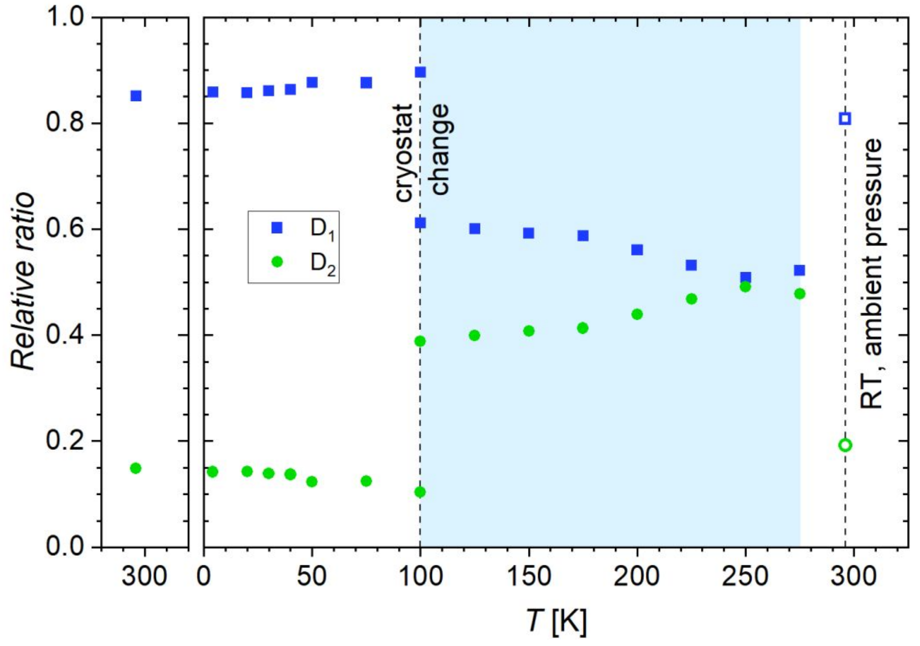

Figure S33. Relative intensity of the components in the Fe-ICR-20, based on the zero-field spectra. At 296 K, the filled points correspond to the initial measurement (left; Figure S26a) and the empty points to the final spectrum at ambient conditions after removal from the LN_2 cryostat, in which the sample was evacuated at room temperature (right, Figure S26c).

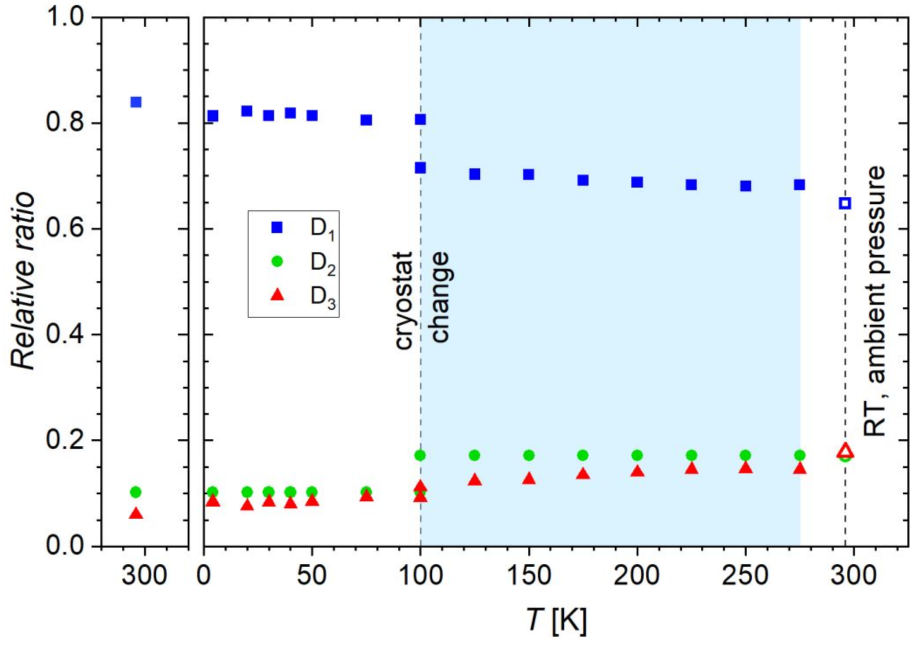

Figure S34. Relative intensity of the components in the Fe-ICR-21, based on the zero-field spectra. At 296 K, the filled points correspond to the initial measurement (left; Figure S26d) and the empty points to the final spectrum at ambient conditions after removal from the LN_2 cryostat, in which the sample was evacuated at room temperature (right, Figure S26f).

8.2. Isomer shift

The temperature dependence of the experimental isomer shift *IS*, see Figure 7b,e in the main text, was fitted by function:

$$IS = \delta + \delta_{\mathrm{SOD}} \quad (7)$$

$$\delta_{\mathrm{SOD}} = -\frac{9k_{\mathrm{B}}E_{\gamma}}{16M_{\mathrm{eff}}\,c^{2}}\left\{\theta_{\mathrm{D}} + 8T\left(\frac{T}{\theta_{\mathrm{D}}}\right)^{3}\int_{0}^{\frac{\theta_{\mathrm{D}}}{T}}\frac{x^{3}}{(e^{x}-1)}\mathrm{d}x\right\}, \quad (8)$$

where δ and δ_{SOD} are the chemical isomer shift (see equation (11) in Sect. 7.4) and second-order Doppler shift, E_{γ} resonant gamma energy (14.41 keV), M_{eff} the effective mass of the resonant nucleus (fixed to 57 atomic mass units), c light velocity and θ_{D} the Debye (Mössbauer) temperature specific for the ^{57}Fe nuclei in their environment.[8]

8.3. Quadrupole splitting

The temperature dependence of the quadrupole splitting, see Figure 7c,f in the main text, was fitted by function:[8,9]

$$QS = \Delta E_{Q}\frac{1 + \exp\left(\frac{-2\Delta E_{1}}{k_{\mathrm{B}}T}\right) + \exp\left(\frac{-2\Delta E_{2}}{k_{\mathrm{B}}T}\right) - \exp\left(\frac{-\Delta E_{1}}{k_{\mathrm{B}}T}\right) - \exp\left(\frac{-\Delta E_{2}}{k_{\mathrm{B}}T}\right) - \exp\left(-\frac{\Delta E_{1}+\Delta E_{2}}{k_{\mathrm{B}}T}\right)}{1+\exp\left(\frac{-\Delta E_{1}}{k_{\mathrm{B}}T}\right)+\exp\left(\frac{-\Delta E_{2}}{k_{\mathrm{B}}T}\right)}$$

(9)

for rhombic crystal field, where ΔE_{Q} is a temperature-independent parameter, and ΔE_{1} and ΔE_{2} are the distances between d_{ε} energy levels split by a rhombic crystal field. For axial crystal field, $\Delta E_{1} = \Delta E_{2} = \Delta E$ and

$$QS = \Delta E_Q \frac{1 - \exp\left(\frac{-\Delta E}{k_\mathrm{B}T}\right)}{1 + 2\exp\left(\frac{-\Delta E}{k_\mathrm{B}T}\right)} \,. \qquad (10)$$

It is important to note that this simplified model does not take into account the spin-orbit coupling, which is usually $\lambda \approx$ 80–100 cm^{-1} for high-spin Fe^{2+}.

Analysis of the temperature dependence of the quadrupole splitting for the Fe-ICR-20 and Fe-ICR-21 samples yielded the following parameters, respectively: ΔE_Q = 3.15(1) mm s^{-1}, ΔE_1 □ 370 cm^{-1} and ΔE_2 □ 1350 cm^{-1}; and ΔE_Q = 3.07(1) mm s^{-1}, ΔE_1 □ 430 cm^{-1} and ΔE_2 □ 1210 cm^{-1}. The reported ΔE_2 values represent lower estimates, above which the fitted curve shape does not change. These experimental estimates are in agreement, for example, with the values ΔE_Q = 3.05(2) mm s^{-1} for $LiFePO_4$[10] and the values ΔE_1 □ 360 cm^{-1}, ΔE_2 □ 1680 cm^{-1} reported by Ingalls for $FeSO_4$.[9] The fit assuming rhombic distortion of the octahedral surrounding Fe^{2+} ion, with four oxygen atoms in equatorial positions and two nitrogen atoms in axial positions, corresponds much better to the experimental data than the fit assuming only axial distortion of the FeO_4N_2 octahedron, see Figure 7c,f in the main text.

8.4. The spin-Hamiltonian formalism

Although the unpaired electrons of a paramagnetic ion usually give rise to a large magnetic hyperfine field at the nucleus, the Mössbauer spectra of most paramagnetic materials consist of doublets or singlets, because the magnetic relaxation time is so short that the nucleus only experiences the average value of the magnetic hyperfine field. In some cases, the relaxation time is on the same order as the time scale of Mössbauer spectroscopy $\tau_\mathrm{M} \sim$ 140 ns, which results

in broadened spectra. In extreme cases, e.g. in low-temperature studies of magnetically dilute samples, the paramagnetic relaxation time may be long compared to τ_M. The magnetic hyperfine interaction then gives rise to magnetically split spectra whose shape crucially depends on the detailed form of the electronic wave functions, which are determined by the spin Hamiltonian:[11,12,13,14,15,16]

$$\hat{H}_S = \hat{H}_{cf} + \hat{H}_{dd} + \hat{H}_{ex} + \hat{H}_Z,$$

(11)

Here, $\hat{H}_{cf}$ is the crystal field spin Hamiltonian, which can be expressed by:

$$\hat{H}_{cf} = D\left[\hat{S}_z^2 - \frac{1}{3}S(S+1) + \frac{E}{D}\left(\hat{S}_x^2 - \hat{S}_y^2\right)\right]$$

$$+ \frac{1}{6}a\left[\hat{S}_\xi^4 + \hat{S}_\eta^4 + \hat{S}_\zeta^4 - \frac{1}{5}S(S+1)(3S^2+3S-1)\right], \qquad (12)$$

where $\hat{\boldsymbol{S}}$ is the ionic spin and a is the ZFS parameter describing the strength of the cubic crystal field contribution. The coordinate system of the cubic crystal field (ξ,η,ζ) may differ from that used to describe the axial and the rhombic crystal field interactions (x, y, z). The dipole and exchange interactions of an ion, i, with the neighbouring magnetic ions, j, can be described by the Hamiltonians $\hat{H}_{dd}$ and $\hat{H}_{ex}$:

$$\hat{H}_{dd} = \frac{\mu_0}{4\pi}\sum_{i\neq j}\frac{g_i g_j \mu_B^2}{r_{ij}^5}\left[\hat{\boldsymbol{S}}_j r_{ij}^2 - 3\boldsymbol{r}_{ij}\left(\hat{\boldsymbol{S}}_j \boldsymbol{r}_{ij}\right)\right]\hat{\boldsymbol{S}}_i \qquad (13)$$

$$\hat{H}_{ex} = -\hat{\boldsymbol{S}}_i \sum_{i\neq j} J_{ij}\hat{\boldsymbol{S}}_j,$$

(14)

where μ_0 is the vacuum permeability, $\boldsymbol{r}_{ij}$ is the vector connecting the *i* and *j* ions, μ_B is the Bohr magneton, and J_{ij} is the exchange coupling constant. If an external magnetic field $\boldsymbol{B}$ is applied, one has to include the Zeeman Hamiltonian:

$$\hat{H}_\mathrm{Z} = \mu_\mathrm{B}\left(g_x B_x \hat{S}_x + g_y B_y \hat{S}_y + g_z B_z \hat{S}_z\right). \tag{15}$$

The free electron value of the *g*-factor corresponds to $g_e = 2.0023$.

For the evaluation of magnetically split Mössbauer spectra within the spin-Hamiltonian formalism, the purely $\hat{\boldsymbol{S}}$-dependent Hamiltonian $\hat{H}_\mathrm{S}$ must be extended by an appropriate nuclear Hamiltonian $\hat{H}_\mathrm{N}$ for the nuclear spin $\hat{\boldsymbol{I}}$:

$$\hat{H}_\mathrm{N} = \hat{H}_\mathrm{hf} + \hat{H}_\mathrm{Q} + \hat{H}_\mathrm{ZN}\,. \tag{16}$$

The leading term in $\hat{H}_\mathrm{N}$ is usually the magnetic hyperfine coupling $\hat{H}_\mathrm{hf}$, which connects the electron spin $\hat{\boldsymbol{S}}$ and the nuclear spin $\hat{\boldsymbol{I}}$:

$$\hat{H}_\mathrm{hf} = \mu_\mathrm{N} g_\mathrm{N} \hat{\boldsymbol{S}} \cdot \bar{\bar{\boldsymbol{A}}} \cdot \hat{\boldsymbol{I}}\,. \tag{17}$$

It is parameterized by the hyperfine coupling tensor $\bar{\bar{\boldsymbol{A}}}$ (which for Fe^{3+} in the high-spin state often can be approximated by a scalar), g_N is the nuclear Landé factor for the ground state or the excited state of the nucleus, and μ_N is the nuclear magneton. The electric quadrupole interaction can be described by the Hamiltonian $\hat{H}_\mathrm{Q}$:

$$\hat{H}_\mathrm{Q} = \frac{eQ}{4I(2I-1)} \sum_{i,j=x,y,z} V_{ij} \left[\hat{I}_i \hat{I}_j + \hat{I}_j \hat{I}_i - \delta_{ij} \frac{2}{3} I(I+1)\right], \tag{18}$$

where Q is the nuclear quadrupole moment, V_{ij} is the ij component of the electric field gradient (EFG) tensor. The Hamiltonian $\hat{H}_\mathrm{Q}$ can be rewritten in the principal (main) axes of the EFG tensor:

$$\hat{H}'_\mathrm{Q} = \frac{eQV_{zz}}{4I(2I-1)}\left[3\hat{I}_z^2 - I(I+1) + \eta\left(\hat{I}_x^2 - \hat{I}_y^2\right)\right], \quad (18a)$$

where $\eta = \left(V_{xx} - V_{yy}\right)/V_{zz}$ is the asymmetry parameter, $0 \leq \eta \leq 1$. For a purely electric quadrupole interaction, the Mössbauer spectrum shows quadrupole splitting ΔE_Q given by:

$$\Delta E_\mathrm{Q} = \frac{1}{2}eQV_{zz}\sqrt{\left(1 + \frac{\eta^2}{3}\right)}\,. \quad (19)$$

Finally, if an external magnetic field $\boldsymbol{B}$ is applied, one has to include the nuclear Zeeman Hamiltonian:

$$\hat{H}_\mathrm{ZN} = \mu_\mathrm{N} g_\mathrm{N}\left(B_x\hat{I}_x + B_y\hat{I}_y + B_z\hat{I}_z\right).$$

(20)

The electric monopole interaction, manifested in the spectra as the isomer shift δ (cf. Eq. 7) can be omitted in the nuclear Hamiltonian since it is independent of the nuclear spin magnetic quantum number M_I. It can subsequently be added as an increment δ to the transition energies between the nuclear ground and excited states, shifting each of the obtained Mössbauer lines:

$$\delta = \frac{Ze^2}{6\varepsilon_0}\left(\langle r_e^2\rangle - \langle r_g^2\rangle\right)\{|\psi_A(0)|^2 - |\psi_S(0)|^2\}, \quad (21)$$

where Ze is the total charge of the nucleus, ε_0 is vacuum permittivity, $\langle r_g^2\rangle$ and $\langle r_e^2\rangle$ are mean square radii of the nucleus in the ground and excited states, $e|\psi_S(0)|$ and $e|\psi_A(0)|$ are

electronic charge density at the nucleus in the source and absorber (i.e., the sample), respectively.

The most straightforward basis for the diagonalization of $\hat{H}_S + \hat{H}_N$ are the product functions $|S,M_S\rangle \times |I,M_I\rangle$, which yield $(2S+1)(2I+1)$ eigenstates and eigenvalues. The interpretation of Mössbauer spectra is facilitated if the nuclear spin is decoupled from the electronic spin. This can be achieved by applying a weak field of >20 mT, which is sufficient to make the electronic Zeeman term much larger than the magnetic hyperfine coupling (recall that the nuclear magneton is about 2000 times smaller than the electron Bohr magneton). Thus, the hyperfine coupling term, which is essential for the nuclear eigenvalues, needs not to be considered in the determination of the purely $\hat{\boldsymbol{S}}$-dependent electronic Hamiltonian $\hat{H}_S$. Instead, it can be treated as a part of the nuclear Hamiltonian, for which the spin operator $\hat{\boldsymbol{S}}$ is replaced by its expectation values $\langle\hat{\boldsymbol{S}}\rangle_i$ for each of the $2S+1$ eigenstates of $\hat{H}_S$. With this approximation, the magnetic hyperfine coupling Hamiltonian $\hat{H}_{\mathrm{hf}}$ can be rewritten as:

$$\hat{H}_{\mathrm{hf},i} = \mu_N g_N \hat{\boldsymbol{I}} \boldsymbol{B}_{\mathrm{hf},i}\ , \qquad \boldsymbol{B}_{\mathrm{hf},i} = \frac{-\bar{\bar{\boldsymbol{A}}}\langle\hat{\boldsymbol{S}}\rangle_i}{\mu_{\mathrm{N}} g_{\mathrm{N}}}, \text{ for } i = 1,\ldots, 2S+1 \qquad (22)$$

where $\boldsymbol{B}_{\mathrm{hf},i}$ is the hyperfine magnetic field at the nucleus for the respective eigenstate i of $\hat{H}_S$. The nuclear Hamiltonian $\hat{H}_N$ can therefore be rewritten in the form:

$$\hat{H}_{\mathrm{N},i} = \mu_{\mathrm{N}} g_{\mathrm{N}} \hat{\boldsymbol{I}}\left(\boldsymbol{B}_{\mathrm{hf},i} + \boldsymbol{B}\right) + \hat{H}_{\mathrm{Q}} = \mu_{\mathrm{N}} g_{\mathrm{N}} \hat{\boldsymbol{I}} \boldsymbol{B}_{\mathrm{eff},i} + \hat{H}_{\mathrm{Q}} \qquad (23)$$

where $\boldsymbol{B}_{\mathrm{eff},i}$ is the effective magnetic field at the nucleus.

By decoupling nuclear and electronic spins, the evaluation of Mössbauer spectra is reduced to the independent diagonalization of the $2S+1$ eigenvalues E_i of $\hat{H}_S$ and the $2I+1$ eigenvalues

for the nuclear ground state ($I_g = 1/2$) and excited state ($I_e = 3/2$). The eigenfunctions of $\hat{H}_S$ yield the electronic spin expectation values $\langle\hat{\boldsymbol{S}}\rangle_i$, which represent the microscopic magnetic moments $\boldsymbol{m}_i$ of the spin states. If the electronic spin states are stationary due to *slow spin relaxation*, separate Mössbauer components have to be evaluated for each of the electronic states, and then these $2S+1$ components are superimposed using appropriate Boltzmann factors $\rho_i = \exp\left(-E_i/k_B T\right)/\sum_j \exp\left(-E_j/k_B T\right)$ for the actual temperature T. If, moreover, the sample is a powder, which is usually the case, it is not sufficient to calculate the Mössbauer spectrum for a single orientation of the applied field, but it is necessary to sum the contributions for all orientations of the magnetic field with respect to the individual crystallites to obtain the final powder spectrum. If the fluctuation rate of electronic spin exceeds the Mössbauer time scale, a *fast spin relaxation* approach can be applied. In this case, the thermal average $\langle\hat{\boldsymbol{S}}\rangle_T$ of the $2S+1$ spin expectation values:

$$\langle\hat{S}\rangle_T = \sum_i \langle\hat{\boldsymbol{S}}\rangle_i \frac{\exp\left(-E_i/k_B T\right)}{\sum_j \exp\left(-E_j/k_B T\right)}$$

(24)

can be substituted in (22):

$$\boldsymbol{B}_{hf} = \frac{-\bar{\bar{\boldsymbol{A}}}\langle\hat{\boldsymbol{S}}\rangle_T}{\mu_N g_N}.$$

(25)

From (23) we get:

$$\hat{H}_N = \mu_N g_N \hat{\boldsymbol{I}}(\boldsymbol{B}_{hf} + \boldsymbol{B}) + \hat{H}_Q = \mu_N g_N \hat{\boldsymbol{I}} \boldsymbol{B}_{eff} + \hat{H}_Q \quad (26)$$

and a single Mössbauer spectrum is obtained from all electronic states together instead of $2S + 1$ separate subspectra. Averaging over an angular grid on the unit sphere yields the powder average.

8.5. The temperature-dependent spectra

The Mössbauer spectra acquired at 6 T, which correspond to the fitted parameters in Table S7, are shown in Figures S34 and S35. The spectra up to 20 K were fitted with the Paramagnetic Hyperfine Structure (PHS) model, those between 30 and 100 K by the static Hamiltonian with dominating electric quadrupole interaction and a distribution of magnetic hyperfine fields.

In addition to the parameters provided in Table S7, the PHS model also allows the determination of the Euler angles – a rotation that brings the crystal field system into the eigensystem of the $\bar{\bar{\boldsymbol{V}}}$ or $\bar{\bar{\boldsymbol{A}}}$ tensors. For the EFG tensor, the Euler angles are $\theta_V = 46°$, $\gamma_V = 180°$, $\varphi_V = 180°$ for Fe-ICR-20 and $\theta_V = 162°$, $\gamma_V = –51°$, $\varphi_V = –101°$ for Fe-ICR-21. In the case of the hyperfine coupling tensor, the angles were refined to $\theta_A = 92°$, $\gamma_A = 66°$, $\varphi_A = –57°$ for Fe-ICR-20 and $\theta_A = 12°$, $\gamma_A = –132°$, $\varphi_A = –100°$ for Fe-ICR-21. However, although the calculated error is small (~1°), the real error can reach units of degrees for θ and tens of degrees for γ and φ.

Table S7. The hyperfine parameters of the main component C_1 determined from the ^{57}Fe Mössbauer spectra of Fe-ICR-20 and Fe-ICR-21 collected at B_{ext} = 6 T.

T [K]	IS [mm s^{-1}]	V_{zz} [10^{21}V m^{-2}]	η	A_{xx} [T]	A_{yy} [T]	A_{zz} [T]	$\langle B_{eff} \rangle$ [T]
**Fe-ICR-20**							
4.2	1.30(2)	18.7(3)	0.30(1)	-10.2(2)	-25.5(5)	-17.3(3)	*17.6(1)*
10	1.30(2)	18.7(3)	0.30(1)	-9.5(1)	-15.8(4)	-13.8(4)	*13.0(2)*
20	1.29(2)	18.7(3)	0.30(1)	-6.7(1)	-9.9(1)	-7.8(1)	*8.1(1)*
30	1.29(2)	18.8(2)	0.22(1)				5.2(1)
40	1.29(2)	18.8(2)	0.22(1)				3.1(1)

50	1.29(2)	18.8(2)	0.22(1)				2.6(1)
60	1.29(2)	18.7(2)	0.22(1)				2.8(1)
80	1.29(2)	18.7(2)	0.22(1)				3.9(1)
100	1.28(2)	18.4(2)	0.22(1)				4.4(1)
**Fe-ICR-21**							
4.2	1.31(2)	18.3(2)	0.26(1)	-10.8(1)	-19.5(1)	-30.4(1)	*20.2(1)*
10	1.30(2)	18.3(2)	0.26(1)	-3.5(5)	-16.5(1)	-26.9(2)	*15.7(2)*
20	1.29(2)	18.3(2)	0.26(1)	-2.1(3)	-9.1(1)	-16.2(1)	*9.2(1)*
30	1.29(2)	18.2(2)	0.25(1)				4.5(1)
40	1.29(2)	18.3(2)	0.25(1)				2.5(1)
50	1.29(2)	18.3(2)	0.25(1)				2.3(1)
60	1.28(2)	18.3(2)	0.25(1)				2.5(1)
80	1.28(2)	17.9(2)	0.25(1)				3.4(1)
100	1.27(2)	17.6(2)	0.25(1)				3.9(1)

The parameter designations: *IS* – experimental isomer shift, V_{zz} – the main component of the EFG tensor, $\eta = (V_{xx} - V_{yy})/V_{zz}$ – asymmetry parameter, $0 \leq \eta \leq 1$, $\langle B_{\mathrm{eff}} \rangle$ – the mean value of the effective hyperfine magnetic field at ^{57}Fe nuclei, $B_{\mathrm{eff}} = |\boldsymbol{B}_{\mathbf{hf}} - \boldsymbol{B}_{\mathbf{ext}}|$. The linewidth was fixed to 0.29 mm s^{-1}.

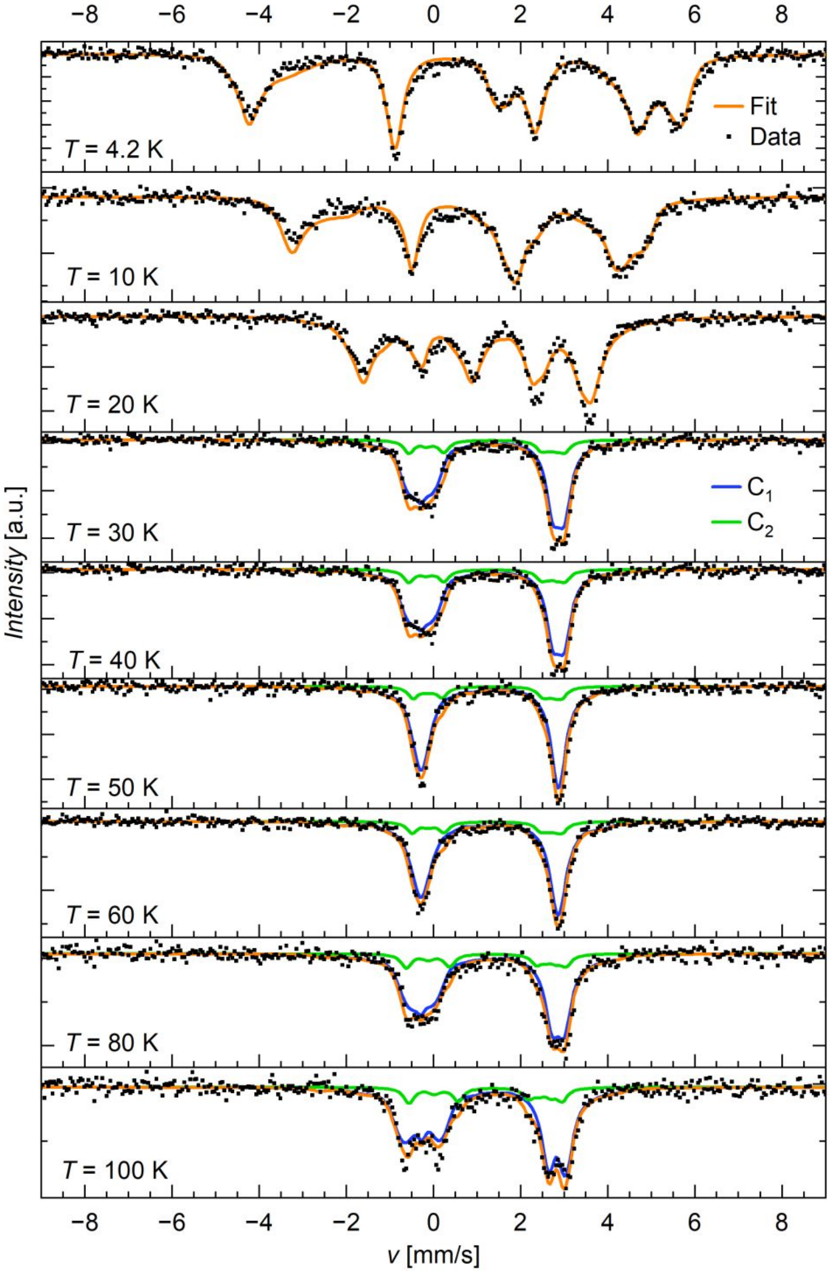

Figure S35. Mössbauer spectra of Fe-ICR-20 acquired at B_{ext} = 6 T. The main ticks on the y-axis correspond to 1% at 4.2–20 K and 2% at 30–100 K.

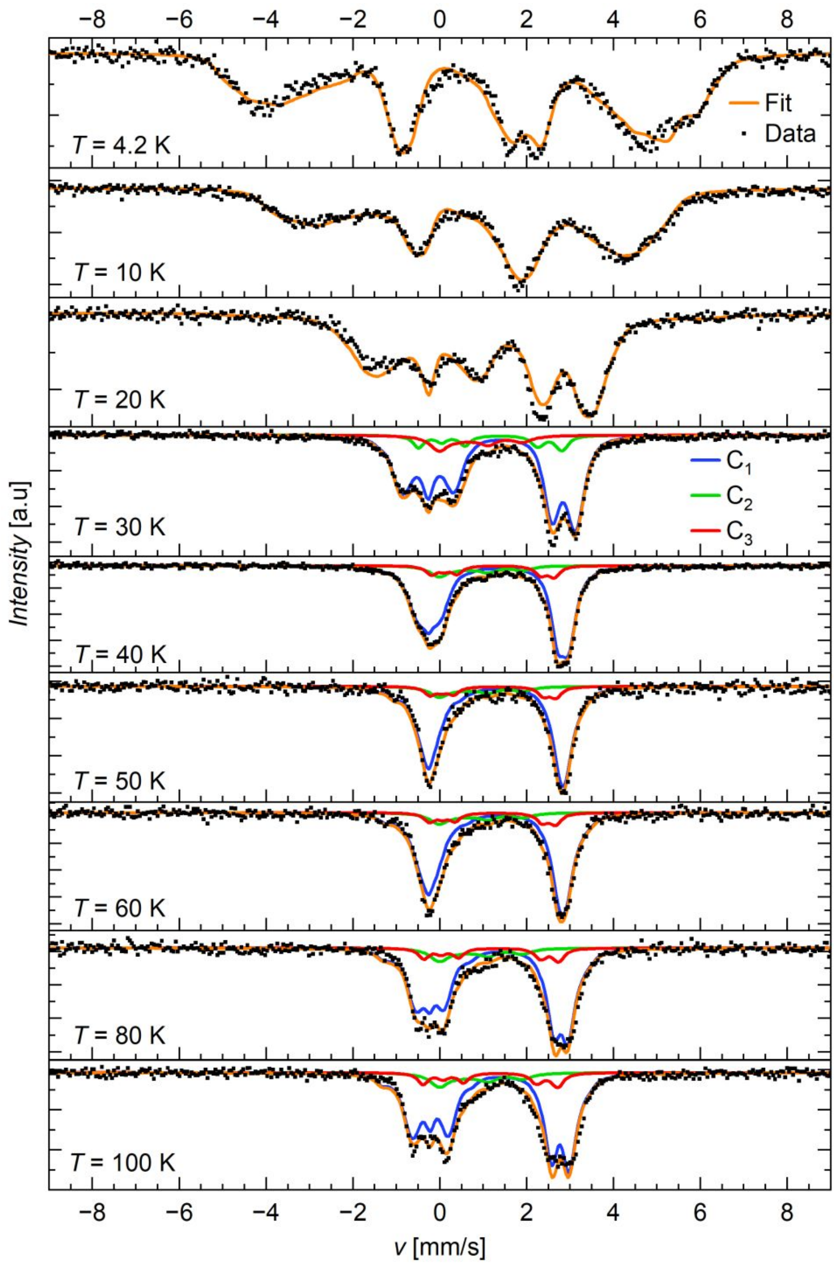

Figure S36. Mössbauer spectra of Fe-ICR-21 acquired at B_{ext} = 6 T. The main ticks on the y-axis correspond to 2%.

8.6. The field-dependent spectra

In addition to the temperature dependence, we also probed the variation of the spectra with the applied magnetic field at 4.2 K, the spectra of Fe-ICR-20 and Fe-ICR-21 are shown in Figures S36 and S37.

We attempted to fit the in-field spectra simultaneously using the PHS model; however, PHS allows only fitting with a single component, disregarding the (unknown) shape of the subspectra corresponding to the C_2 and C_3 components. Moreover, the large relative stress on the low-field spectra leads to deviations of the fitted parameters from the model used in the temperature dependence of spectra acquired at 6 T.

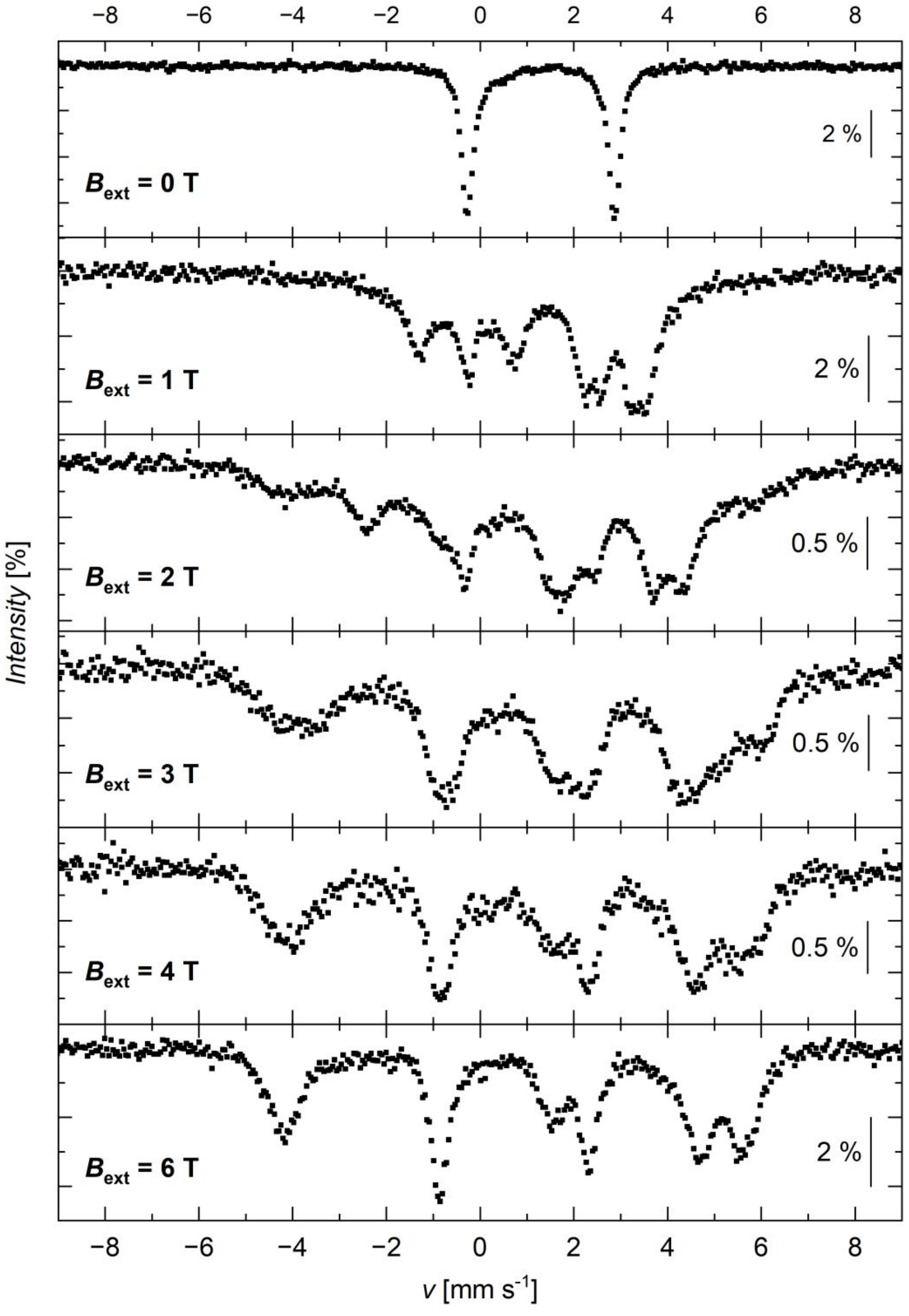

Figure S37. Mössbauer spectra of Fe-ICR-20 acquired at 4.2 K at various applied fields. The vertical lines show the effect of the spectra.

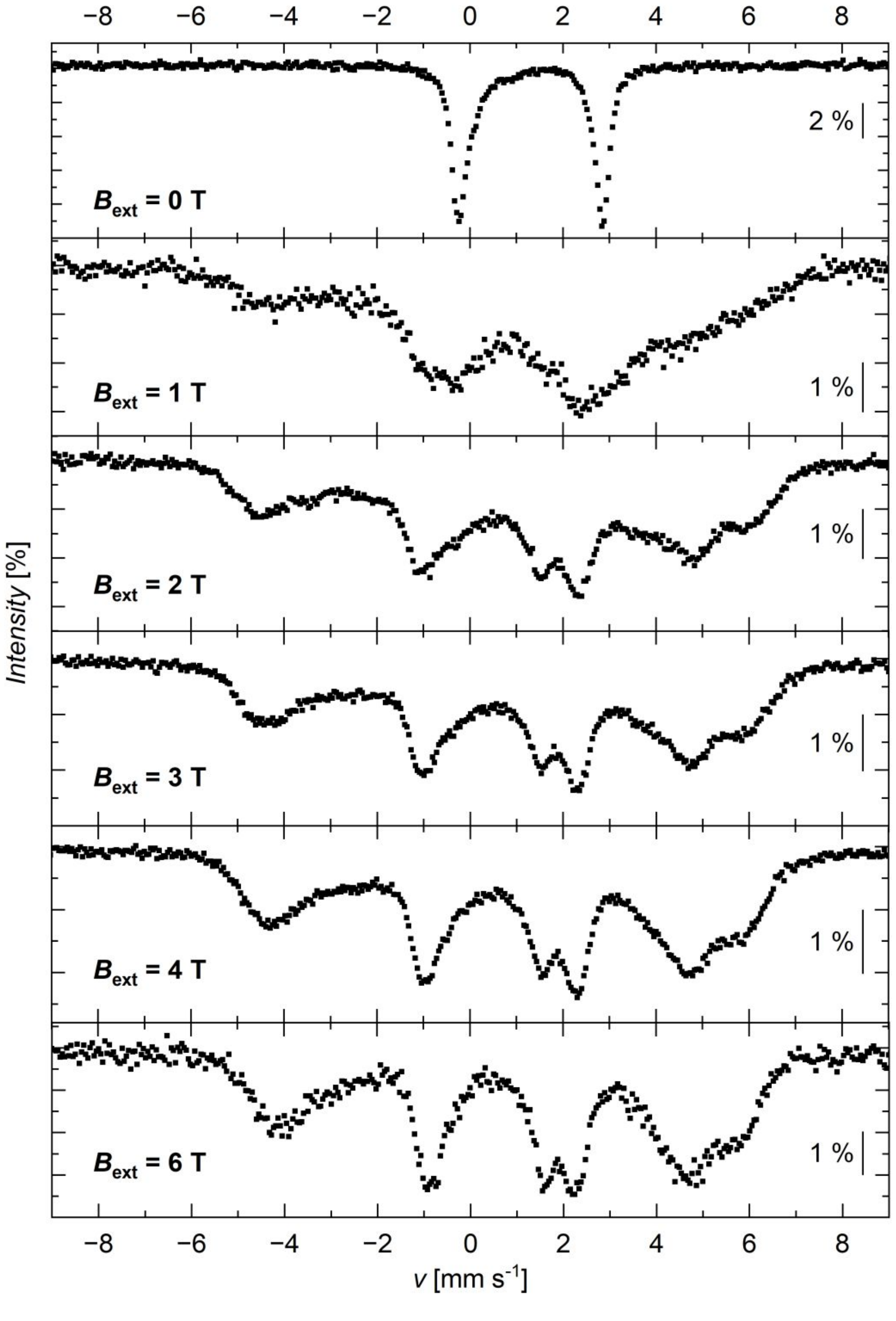

Figure S38. Mössbauer spectra of Fe-ICR-20 acquired at 4.2 K at various applied fields. The vertical lines show the effect of the spectra.

9. Proton conductivity

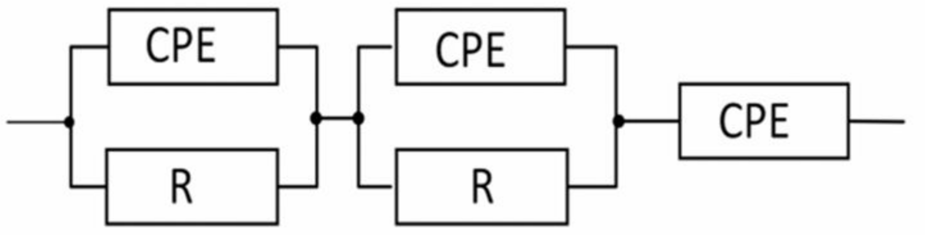

Figure S39. The equivalent circuit used for the fitting of Nyquist plots. R represents resistance and CPE represents constant phase element.

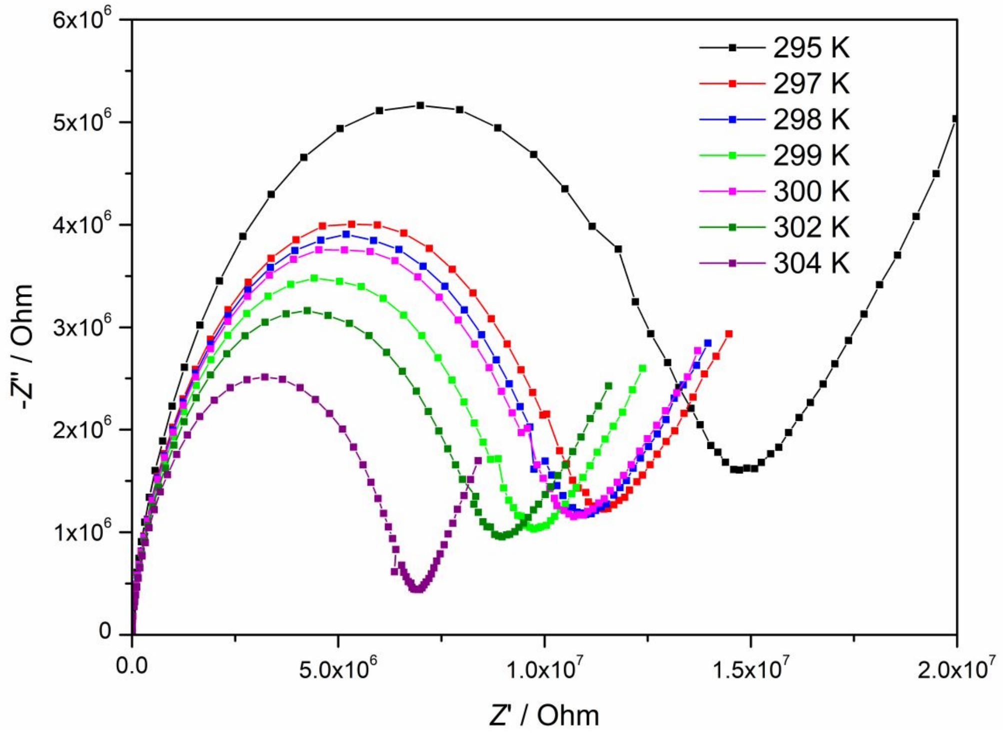

Figure S40. The Nyquist plots for Co-ICR-20 measured at different temperature and at the relative humidity of 75%.

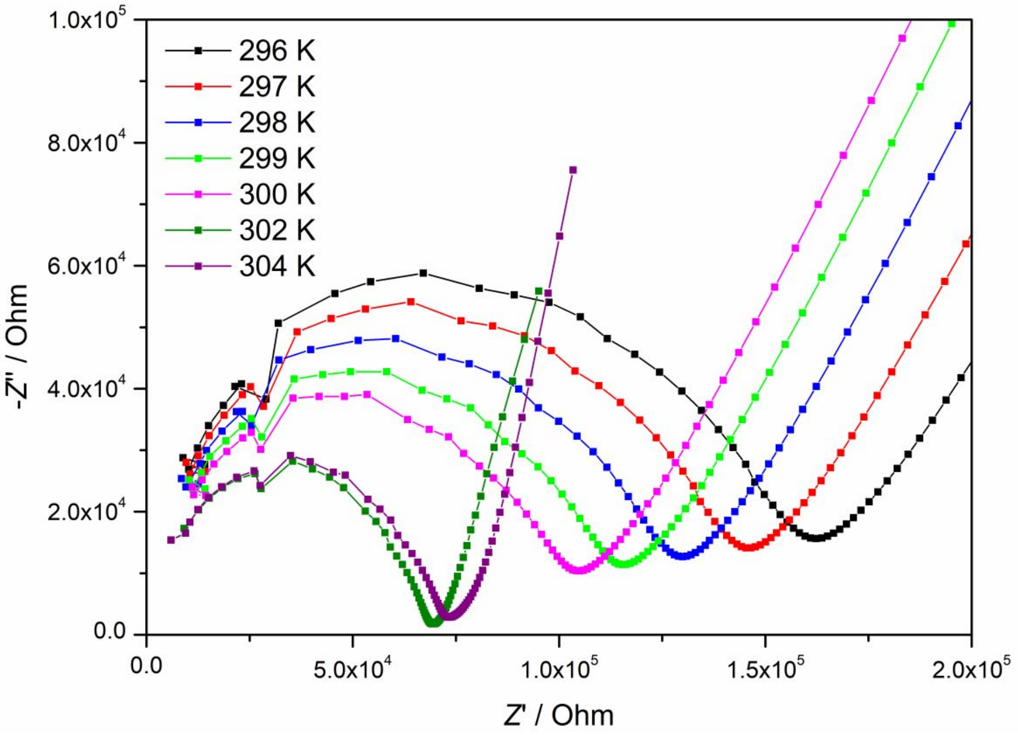

Figure S41. The Nyquist plots for Co-ICR-20 measured at different temperature and at the relative humidity of 92%.

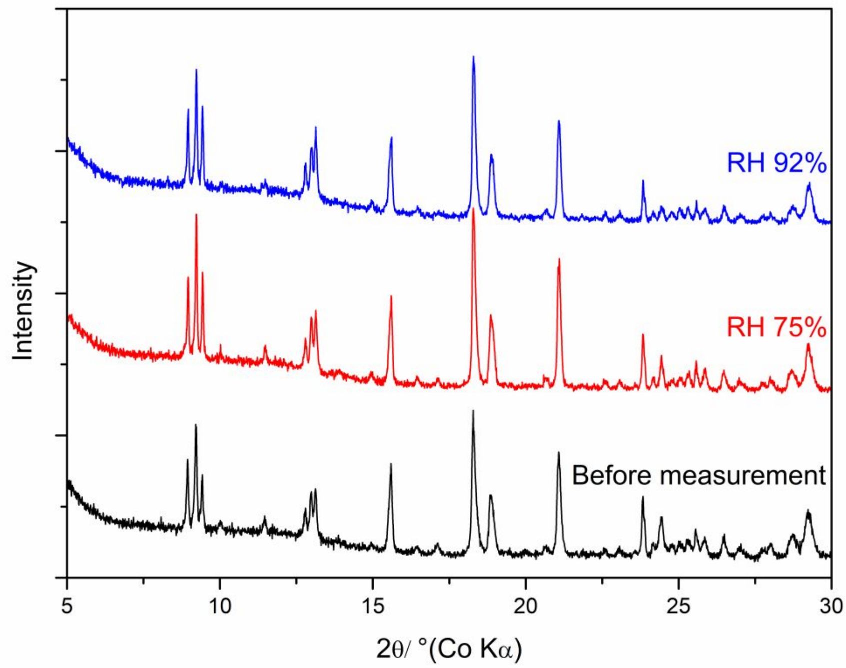

Figure S42. Comparison of PXRD patterns of Co-ICR-20 pellets measured before the measurement of proton conductivity (black) and after the measurement at 75% relative humidity (red) and 92% relative humidity (blue).

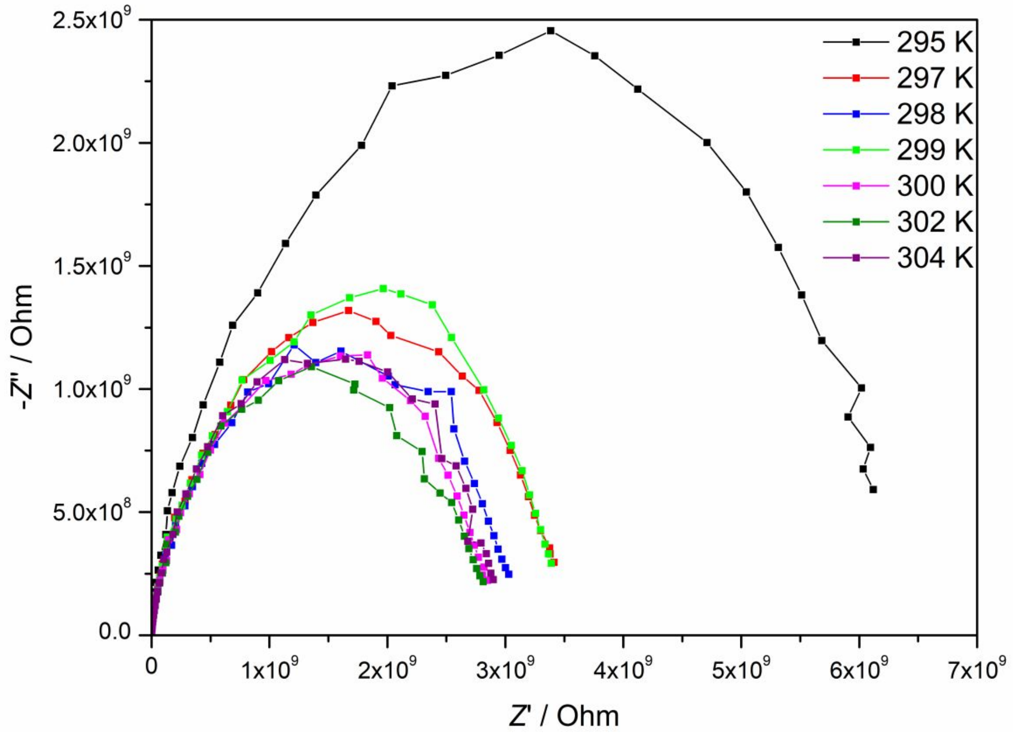

Figure 43. The Nyquist plots for Co-ICR-21 measured at different temperature and at the relative humidity of 75%.

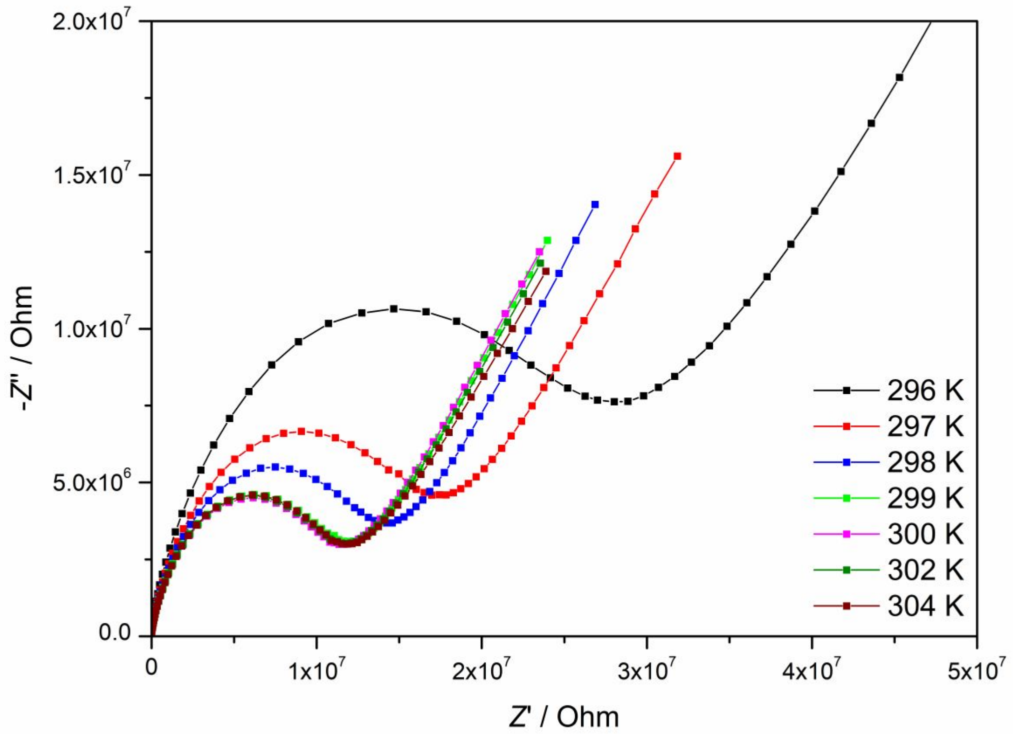

Figure S44. The Nyquist plots for Co-ICR-21 measured at different temperature and at the relative humidity of 92%.

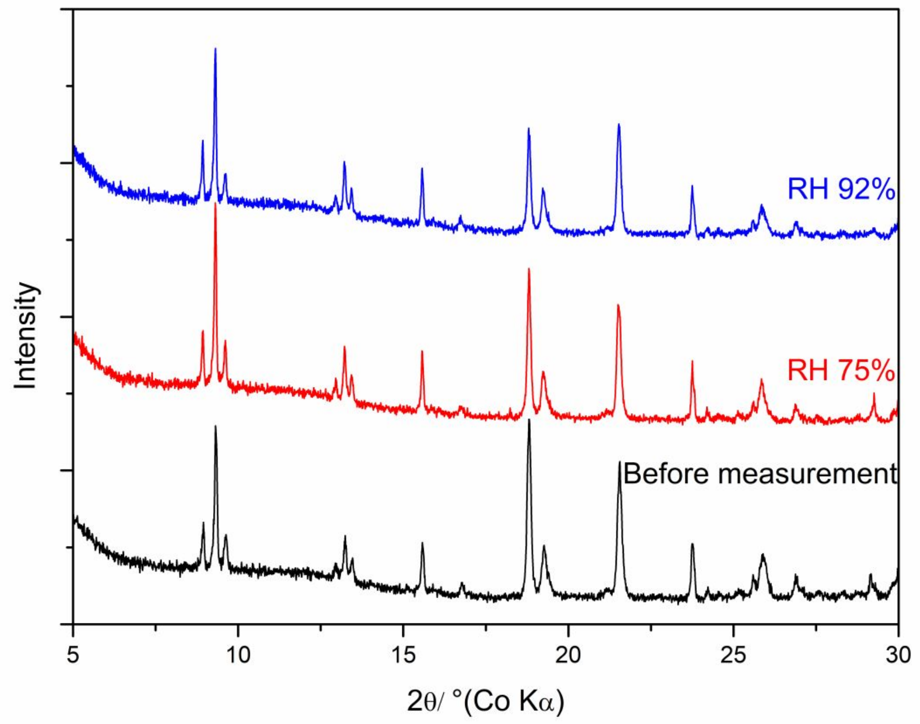

Figure S45. Comparison of PXRD patterns of Co-ICR-21 pellets measured before the measurement of proton conductivity (black) and after the measurement at 75% relative humidity (red) and 92% relative humidity (blue).

REFERENCES:

[1] Santana, M. D.; García, G.; Lozano, A. A.; López, G.; Tudela, J.; Pérez, J.; García, L.; Lezama, L.; Rojo, T. Pentacoordinate Nickel^{II} Complexes Double Bridged by Phosphate Ester

or Phosphinate Ligands: Spectroscopic, Structural, Kinetic, and Magnetic Studies. Chemistry – A European Journal, 2004, 10, 1738–1746. https://doi.org/10.1002/chem.200305367.

[2] de Jongh, L. J.; Miedema, A. R. Experiments on Simple Magnetic Model Systems. Advances in Physics, 1974, 23, 1–260. https://doi.org/10.1080/00018739700101558.

[3] Fisher, M. E. Magnetism in One-Dimensional Systems—The Heisenberg Model for Infinite Spin. American Journal of Physics, 1964, 32, 343–346. https://doi.org/10.1119/1.1970340.

[4] Hiller, W.; Straehle, J.; Datz, A.; Hanack, M.; Hatfield, W. E.; Ter Haar, L. W.; Guetlich, P. Synthesis, Structure, and Magnetic Properties of Catena-(.Mu.-Oxo)(Hemiporphyrazinato)Iron(IV), the First Polymeric .Mu.-Oxo-Bridged Complex of Iron. Journal of the American Chemical Society, 1984, 106, 329–335. https://doi.org/10.1021/ja00314a013.

[5] Chilton, N. F.; Anderson, R. P.; Turner, L. D.; Soncini, A.; Murray, K. S. PHI: A Powerful New Program for the Analysis of Anisotropic Monomeric and Exchange-coupled Polynuclear d- and f-block Complexes. Journal of Computational Chemistry, 2013, 34, 1164–1175. https://doi.org/10.1002/jcc.23234.

[6] Blackmore, W. J. A.; Brambleby, J.; Lancaster, T.; Clark, S. J.; Johnson, R. D.; Singleton, J.; Ozarowski, A.; Schlueter, J. A.; Chen, Y.-S.; Arif, A. M.; Lapidus, S.; Xiao, F.; Williams, R. C.; Blundell, S. J.; Pearce, M. J.; Lees, M. R.; Manuel, P.; Villa, D. Y.; Villa, J. A.; Manson, J. L.; Goddard, P. A. Determining the Anisotropy and Exchange Parameters of Polycrystalline Spin-1 Magnets. New Journal of Physics, 2019, 21, 093025. https://doi.org/10.1088/1367-2630/ab3dba.

7 Sanselme, M.; Grenèche, J.-M.; Riou-Cavellec, M.; Férey, G. The First Ferric Carboxylate with a Three-Dimensional Hydrid Open-Framework (MIL-82): Its Synthesis, Structure, Magnetic Behavior and Study of Its Dehydration by Mössbauer Spectroscopy. Solid State Sciences, 2004, 6, 853–858. https://doi.org/10.1016/j.solidstatesciences.2004.04.001.

8 Gütlich, P.; Bill, E.; Trautwein, A. X. Mössbauer Spectroscopy and Transition Metal Chemistry; Springer Berlin Heidelberg, 2011. https://doi.org/10.1007/978-3-540-88428-6.

9 Ingalls, R. Electric-Field Gradient Tensor in Ferrous Compounds. Physical Review, 1964, 133, A787–A795. https://doi.org/10.1103/physrev.133.a787.

10 Kmječ, T.; Kohout, J.; Dopita, M.; Veverka, M.; Kuriplach, J. Mössbauer Spectroscopy of Triphylite (LiFePO4) at Low Temperatures. Condensed Matter, 2019, 4, 86. https://doi.org/10.3390/condmat4040086.

11 Abragam, A., Bleaney, B.: Electron Paramagnetic Resonance of Transition Ions. Dover, New York (1986), ISBN: 978-0486651064

12 Klencsár, Z.; Kuzmann, E.; Vértes, A. User-Friendly Software for Mössbauer Spectrum Analysis. Journal of Radioanalytical and Nuclear Chemistry Articles, 1996, 210, 105–118. https://doi.org/10.1007/bf02055410.

13 Slichter, C. P. Principles of Magnetic Resonance; Springer Berlin Heidelberg, 1990. https://doi.org/10.1007/978-3-662-09441-9.

14 Boča, R. Mean and Differential Magnetic Susceptibilities in Metal Complexes. Coordination Chemistry Reviews, 1998, 173, 167–283. https://doi.org/10.1016/s0010-8545(98)00139-8.

15 Boča, R. Zero-Field Splitting in Metal Complexes. Coordination Chemistry Reviews, 2004, 248, 757–815. https://doi.org/10.1016/j.ccr.2004.03.001.

16 O. Kahn, Molecular Magnetism, VCH Publishers, Inc., Cambridge (1993), ISBN: 1-56081-566-3
